# Quantum algorithmic measurement

**DOI:** 10.1038/s41467-021-27922-0

**Published:** 2022-02-16

**Authors:** Dorit Aharonov, Jordan Cotler, Xiao-Liang Qi

**Affiliations:** 1 School of Computer Science and Engineering, The Hebrew University of Jerusalem, The Edmond J. Safra Campus, 9190416 Jerusalem, Israel; 2 Society of Fellows, Harvard University, Cambridge, MA 02138 USA; 3 Stanford Institute for Theoretical Physics, Stanford University, Stanford, CA 94305 USA

**Keywords:** Computer science, Information theory and computation, Quantum information

## Abstract

There has been recent promising experimental and theoretical evidence that quantum computational tools might enhance the precision and efficiency of physical experiments. However, a systematic treatment and comprehensive framework are missing. Here we initiate the systematic study of experimental quantum physics from the perspective of computational complexity. To this end, we define the framework of quantum algorithmic measurements (QUALMs), a hybrid of black box quantum algorithms and interactive protocols. We use the QUALM framework to study two important experimental problems in quantum many-body physics: determining whether a system’s Hamiltonian is time-independent or time-dependent, and determining the symmetry class of the dynamics of the system. We study abstractions of these problems and show for both cases that if the experimentalist can use her experimental samples coherently (in both space and time), a provable exponential speedup is achieved compared to the standard situation in which each experimental sample is accessed separately. Our work suggests that quantum computers can provide a new type of exponential advantage: exponential savings in resources in quantum experiments.

## Abstract (web summary)

Applying the language of computational complexity to study real-world experiments requires a rigorous framework. Here, the authors provide such a framework and establish that there can be an exponential savings in resources if an experimentalist can entangle apparatuses with experimental samples.

## Introduction

Since the early days of physics, innovative methods have been invented to interrogate physical systems via *experiments*. By example, some experiments measure constants of nature, such as the speed of light or the charge the electron; others quantify dynamical properties of systems, such as rates of chemical reactions; yet others infer structural properties, like the symmetry group of a crystal. Often experiments seek to learn more abstract information, such as the chain of chemical reactions that comprise photosynthesis, or whether Yang–Mills theory describes the strong force. We can ask: what exactly is an experiment, in its full scope of generality?

Over the past two decades, we have witnessed a new era in this respect, in which ingredients, ideas and concepts originating from the world of quantum computation are being incorporated into the experimental physics toolbox^1–25^. This body of work constitutes strong evidence that leveraging quantum computational resources to manipulate and measure physical systems may dramatically enhance experimental capabilities. However, what is the extent of these improvements? And what is the right model in which one can study the possibilities and limitations of these more general quantum experiments? A theoretical framework for the systematic study of general quantum experiments, and the resources they require, does not exist yet. The *quantum Church Turing thesis*^26^ suggests that any physical process can be efficiently (i.e., with only polynomial overhead) simulated by a *quantum* algorithm applying local quantum gates. This observation not only constitutes the pillar on which the entire theory of quantum algorithms and quantum computational complexity stands, but it has also had a profound impact on our understanding of quantum physics in the past two decades (see, e.g. ref. ^27^). We take this insight one step further to the setting of quantum experiments.

In this work we provide a computational complexity framework for quantum experiments. We argue that (i) Experiments should be viewed as generalizations of quantum algorithms. They can be studied and designed abstractly, using quantum gates and circuits. (ii) One can study the *computational complexity* of quantum experiments, as an extension of the way the computational complexity of quantum algorithms is studied. We thus use the language of *computational complexity* to define an abstract model of general experiments, which we call *quantum algorithmic measurements*, or QUALMs, which we hypothesize is universal for quantum experiments. Initial seeds for our approach were given in^24,28^.

## Results

### The quantum algorithmic measurement framework

Our starting point is the postulate that the goal of any physical experiment is to compute a function from an input physical system to a classical outcome. The value of the function holds the information that the experimentalist wishes to extract about that physical system. In contrast to standard (quantum or classical) algorithms, the input for a physical experiment is a physical system; the experimentalist is not given a full classical description of it. Instead, access to the physical input system is mediated by quantum operations and measurements, which in general provide only limited information.

A first natural attempt is to model experiments as “black box” quantum algorithms: queries to the physical system (namely applications of the unknown superoperator describing the system) are interlaced with controlled quantum computations applied by the experimentalist. However, it turns out that this model is not general enough to describe all quantum experiments; in particular, it does not allow the physical system being studied to maintain its own inaccessible (or private) quantum memory.

**Fig. 1:**
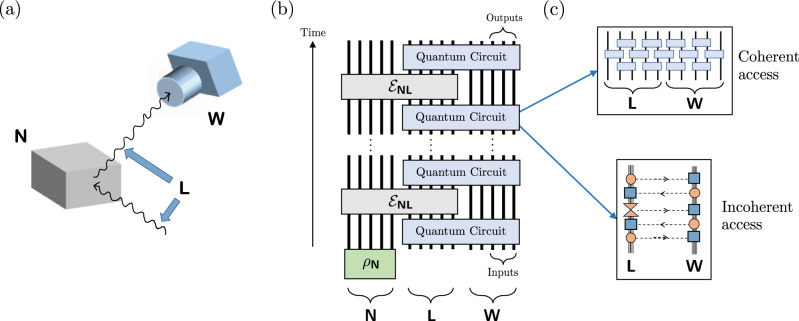
Schematic of a quantum algorithmic measurement. **a** Illustration of a QUALM for X-ray diffraction, where **N** is the crystal sample, **L** consists of the X-ray photons (including the incoming and outgoing ones), and **W** contains the camera and other lab equipment for taking and processing the image. **b** Schematic illustrating the structure of a QUALM as an interaction between Nature and the experimentalist’s controlled degrees of freedom. Here **N** represents the ‘Nature’ register, **L** is the ‘lab’ register, and **W** is the ‘working space’ register. The experimentalist does not have direct measurement access to the **N** register, which should be thought of as the “hidden” degrees of freedom of the physical system on which the experiment is conducted. The initial state on **N** is *ρ*_**N**_, and the input and output subsets of **W** are specified. **c** Illustration of the coherent and incoherent access QUALMs. Coherent access QUALMs allow for general unitary dynamics on the lab and working spaces. Incoherent access QUALMs only permit classical communication between the lab and working spaces; each orange solid circle is a completely positive (CP) map and each blue box is a completely positive trace-preserving (CPTP) map. At least one of the CP maps between each application of the lab oracle is a complete measurement, indicated by a double triangle. The direction of the arrows in the horizontal dashed lines indicates the direction of classical information flow.

Towards defining a universal model of experiments, consider the concrete example of an X-ray diffraction experiment, performed to determine the crystal structure of a material. The experiment involves a crystal sample; X-ray photons, which exhibit an electromagnetic interaction with the crystal; and a camera as well as other lab equipment, which only interact with the photons (see Fig. 1(a)). This is a very general situation: in a physical experiment, the experimentalist usually cannot fully interact with all degrees of freedom of the physical system she desires to measure. We thus model a general experimental system as consisting of three subsystems (registers). The first is called “Nature”, denoted by **N**, which we view as the system that Nature holds secretly, and to which the experimentalist has no direct access in the experiment (this is the crystal in the above example). Our apparatus in the lab is denoted by **W** for “work space” (e.g. the camera and data processors in the X-ray example). The degrees of freedom that the experimentalist does have access to, but which couple to the hidden degrees of freedom of **N**, comprise the “lab” register **L** (e.g., the X-ray photons).

In the X-ray example, the *input physical system*, which the experimentalist would like to measure or learn about, can be described by the combination of the (unknown) state and structure of the crystal, together with the (unknown) interaction between the crystal and the photons (it is unknown since it is a function of the unknown properties of the crystal). More generally, we model an input physical system by a *lab oracle*. The description of the lab oracle contains the initial state of the hidden degrees of freedom **N**, its dynamics, and the interaction between **N** and **L**.

**Definition 1** (Roughly) A lab oracle is described by a pair ${\sf{LO}}({{{{{\bf{N}}}}}},{{{{{\bf{L}}}}}})=({{{{{{\mathscr{E}}}}}}}_{{{{{{\bf{NL}}}}}}},{\rho }_{{{{{{\bf{N}}}}}}})$, where ${{{{{{\mathscr{E}}}}}}}_{{{{{{\bf{NL}}}}}}}$ is a superoperator acting jointly on **N** and **L**, and *ρ*_**N**_ is the initial state of the **N** system.

Our general model of a physical experiment is described in Fig. 1(b). We model a physical experiment as an interactive protocol applied between the work space **W** and Nature **N**; these two registers communicate using the lab register **L**, which serves as a “message” register. The superoperator ${{{{{{\mathscr{E}}}}}}}_{{{{{{\bf{NL}}}}}}}$ describing the interaction between **L** and **N**, given by the physical system to be measured or probed, is unknown and is viewed as a *black box*, which can be “queried"; namely, it can be applied at will by the experimentalist. The addition of the Nature register **N** allows us to arrive at a rather simple hybrid model, which combines two basic models in computer science: interactive protocols, and black box algorithms.

We next introduce a notion paralleling that of a *computational problem* in the algorithmic world. It is called a Task, and it encapsulates the experimental goal that the experimentalist wishes to achieve. The Task consists of the information that the experimentalist wishes to extract, expressed as a function from lab oracles (capturing physical systems) to classical outputs. It also includes the constraints on the experiment due to various limitations in the lab, specified by the *admissible gate set*. These gates are constrained to not act on **N**, and they can also express additional constraints in the labratory such as geometric restrictions on the interactions.

**Definition 2** (Roughly) A task is a tuple ${\sf{Task}}=({{{{{{\bf{S}}}}}}}_{{{\mathrm{in}}}},{{{{{{\bf{S}}}}}}}_{{{\mathrm{out}}}},f,{{{{{\mathscr{G}}}}}})$, associated with a given system **N** ⊗ **L** ⊗ **W**. Here, **S**_in_, **S**_out_ ⊆ **W** consist of *p* and *q* qubits respectively; *f* is a function

1 $$f:\{{{\sf{LO}}}_{0},{{\sf{LO}}}_{1},{{\sf{LO}}}_{2},...\}\times {\{0,1\}}^{p}\longrightarrow {\{0,1\}}^{q}\ ,$$

and ${{{{{\mathscr{G}}}}}}$ is a set of admissible gates on **L** ⊗ **W**. In the domain of *f*, {LO_0_, LO_1_, LO_2_, . . . } is a set of lab oracles.

Note that the function *f* in the above definition has as its inputs not only the lab oracle, but also a classical bit string. The latter should be thought of as additional parameters that describe the desired data, e.g. the specification of the temperature at which the experimentalist may want to perform a certain experiment. While in the above definition the output of *f* is deterministic, a very natural generalization is for the output to be a *probability distribution* over classical output strings; this allows expressing approximated tasks or taking into account finiteness of precision (see discussion in Example 1 in the Supplementary Information).

Finally we can define a QUALM; this specifies an experimental protocol, i.e., a way to implement the experiment such that it achieves the desired task.

**Definition 3** (Roughly) A QUALM(**N**, **L**, **W**) is a specification of a sequence of admissable gates from a set ${{{{{\mathscr{G}}}}}}$ on the subsystems **L, W**, interlaced with applications of a black box operator □ acting on **N**, **L**; some of the qubits (i.e., those in **S**_in_) in the register **W** are marked as inputs and some (i.e., those in **S**_out_) as outputs.

We note that this definition is tightly related to the definitions of quantum strategies^29^ and quantum combs^30^ introduced in the context of quantum interactive protocols or games. A QUALM is designed in order to achieve a specific experimental Task. To see which Task is achieved by the QUALM, we can view the QUALM naturally as a map from the input lab oracle $({{{{{{\mathscr{E}}}}}}}_{{{{{{\bf{NL}}}}}}},{\rho }_{{{{{{\bf{N}}}}}}})$ to a standard quantum circuit, acting on **N** ⊗ **L** ⊗ **W**, whose input bits are in **S**_in_ and output bits are in **S**_out_. **N** is initialized to *ρ*_**N**_, all qubits in **W** and **L** except for **S**_in_ are initialized to 0, and the input to the circuit is given in **S**_in_. The circuit applies to this initial state the gate sequence of the QUALM, where whenever □ appears, ${{{{{{\mathscr{E}}}}}}}_{{{{{{\bf{NL}}}}}}}$ is applied. The output of the circuit is given by measuring **S**_out_ in the computational basis. We say that a QUALM *achieves* a given Task if (i) the sets ${{{{{\mathscr{G}}}}}},{{{{{{\bf{S}}}}}}}_{{{\mathrm{in}}}},{{{{{{\bf{S}}}}}}}_{{{\mathrm{out}}}}$ are the same for the QUALM and the Task, and (ii) for every lab oracle LO and input string *x* to **S**_in_, the result of the measurement of the output qubits **S**_out_ (after the application of the corresponding circuit) is equal (or close, in cases of approximations) to *f*(LO, *x*), with *f* being the Task function.

The computational complexity of a QUALM is the number of gates plus the number of lab oracle applications; the QUALM complexity of the Task is that of the most efficient QUALM that achieves it. We propose that QUALM complexity is a standardized way to quantify and study the (asymptotic behavior of the) cost of achieving an experimental task in the laboratory.

In the Supplementary Information, we provide a versatile set of examples for how different experimental tasks can be viewed as Tasks and be realized by QUALMs. We hypothesis that the QUALM framework is a *universal* model for quantum experiments, in the sense that any physical process realizing an experimental task can be simulated efficiently (i.e., with at most polynomial overhead in all resources) by a QUALM; in other words, we speculate that the quantum Church Turing thesis can be extended from computational problems to experiments, by generalizing quantum algorithms to QUALMs. We stress that all the ingredients included in the QUALM framework seem to be necessary for its universality; in particular, the register **N** is necessary in order to describe some of the more sophisticated physical experiments (see, e.g., the verification example in Supplementary Note B in the Supplementary Information). We thus arrive at a framework that allows us to initiate a rigorous study of the resources required in order to perform physical experimental tasks.

### Exponential advantage of coherent QUALMs

An overwhelming majority of quantum experiments performed in contemporary laboratories are of a much more restricted type than general QUALMs. In those more restricted experiments, which we call *incoherent* QUALMs, the physical system is usually probed by preparing it in some state, possibly letting it evolve, applying a measurement, and then post-processing the measurement’s outcome classically. This may be repeated many times, and the initial state and the basis of measurement may even depend adaptively on previous outcomes. The key point is that these experiments do not utilize coherence between different accesses of the lab oracle, or between the lab space and the working space. We formally define incoherent QUALMs using the notion of LOCC (local operations and classical communication) protocols; see Fig. 1(c).

The first question we choose to address in the QUALM framework is whether generalizing quantum experiments beyond incoherent QUALMs, could lead to significant savings in resources in physics experiments. To this end, we compare incoherent QUALMs to the most general possible QUALMs, which are allowed to leverage a full-fledged quantum computer, apply quantum gates on the lab register during the experiment, and so on. We refer to these general, unrestricted experiments as *coherent* QUALMs.

We study this question in the context of two basic experimental tasks. The first is the task of distinguishing a Floquet system from a random quantum evolution. Roughly speaking, we want to design an experiment that distinguishes a fixed Hamiltonian from a time-dependent, random Hamiltonian. We consider the following toy version of this problem:

**Definition 4** (The fixed unitary problem) (Roughly) Consider two lab oracles LO_0_ and LO_1_, corresponding to two physical systems. The first lab oracle LO_0_ picks a Haar-random unitary, remembers it (forever), and then subsequently applies that *same* unitary to **L** each time the oracle is called. By contrast, the second lab oracle LO_1_ applies a new Haar-random unitary to **L** each time the oracle is called. The goal is to distinguish between LO_0_ and LO_1_ with non-negligible success probability.

There is a very simple coherent QUALM that distinguishes between LO_0_ and LO_1_: just call the lab oracle twice and perform a SWAP test on the two output states. Indeed, variants of the fixed unitary problem and the SWAP protocol have been previously studied (see e.g.^31,32^). However, it turns out that an incoherent QUALM would require exponential resources. Here we provide such an exponential complexity separation. This is implied by the following lower bound:

**Theorem 1** (*Exponential lower bound for incoherent adaptive QUALMs for the fixed unitary problem*) (*Roughly*) *For any incoherent QUALM for the “fixed unitary problem” on ℓ qubits* (*i.e.,* ***L*** *has ℓ qubits*)*, its QUALM complexity is lower bounded by an exponential in ℓ, namely Ω*(*2*^*2ℓ/7*^).

The proof is not too difficult if the incoherent QUALM is non-adaptive, however the argument becomes far more complicated in the adaptive setting. We sketch here the two key points of the proof (See the Methods section and the Supplementary Information for more details).

The first is to reduce incoherent QUALMs to simple measurement QUALMs. In a generic incoherent QUALM, there can be multiple rounds of classical communications between **L** and **W**, before and after each application of the lab oracle (see Fig. 1(c)). However, we show that a generic incoherent QUALM is equivalent to a probabilistic average over a family of “simple measurement QUALMs", which refers to special incoherent QUALMs that simply (i) prepare a state, (ii) apply the lab oracle to that state, (iii) measure the result, and then repeat (i), (ii), (iii) with different settings. The state preparations and measurement bases can depend adaptively on the measurement results of previous steps.

The other key part of the proof is our lower bound for the simple measurement QUALMs required to perform the task. Let *P*_*k*_ be the probability distribution of the *k* measurement results in the case of the lab oracle applying a newly-chosen random unitary each time. *P*_*k*_ is uniformly random. *Q*_*k*_ is the distribution over all *k* intermediate measurement results for the fixed unitary oracle, and we show it is exponentially close to *P*_*k*_. This is achieved using the Weingarten functions *W*(*τ*, *D*); we write:

2 $${Q}_{k}(s)=\mathop{\sum}\limits_{\sigma ,\tau \in {S}^{k}}\,{{{{\mathrm{tr}}}}}\,({A}_{s}\sigma )\,{{{{\mathrm{tr}}}}}\,({B}_{s}{\tau }^{-1})W(\tau {\sigma }^{-1},D)$$

with *A*_*s*_ and *B*_*s*_ operators corresponding to the adaptive choice of preparation state and basis of measurement for each of the oracle calls. *σ*, *τ* are elements of the permutation group. The difficulty here is that in computing the 1-norm distance between *P*_*k*_ and *Q*_*k*_, the sum over *s* cannot be carried over straightforwardly, due to the dependence of the input states and bases of measurement on previous measurement results. A key ingredient in the proof is to show that in the above sum, the term corresponding to each permutation *τ* on the *k* oracle calls can be partitioned to two, such that the sum associated with each part is done one by one over parameters, which are independent from the remaining parameters in the sum.

Our second physically motivated task is to determine the symmetry class of the dynamics of a quantum many-body system. The symmetries of a many-body system are essential to its physical properties, and are the core of all analytic treatments. It is generally difficult to ascertain the symmetries of an uncharacterized quantum system; however, we might intuit that a quantum computer could aid in this endeavor. We study the following toy version of the problem:

**Definition 5** (The Symmetry Distinction Problem) Distinguish, with non-negligible success probability, between three classes of lab oracles: (i) a lab oracle LO_*U*_, which applies a fixed Haar-random unitary to the **L** system; (ii) a lab oracle LO_*O,*_ which applies a fixed Haar-random orthogonal matrix to the system; (iii) a lab oracle LO_Sp,_ which applies a fixed Haar-random symplectic matrix to the system. (Suppose that **L** contains an even number of qubits).

If one is allowed coherent access, then one can use a generalization of the SWAP test (this time on a maximally entangled state and with a little more sophistication) to determine the symmetry type of the lab oracle. However, extending the techniques used in the proof of Theorem 1, we can prove our second main theorem, stating that any incoherent (even adaptive) QUALM for the symmetry distinction problem will have QUALM complexity at least of order Ω(2^2*ℓ*/7^). The idea of the proof is that LO_*U*_, LO_*O*_ and LO_Sp_ are each indistinguishable from the lab oracle LO_1_ that generates a new Haar-random unitary each time it is queries, and so are indistinguishable from one another.

## Discussion

Our motivation in this work is Physics. We argued that recent developments involving computational elements in quantum experiments, suggest a general model for quantum experiments, which clarifies the paradigm of experimentation itself. We have demonstrated an exponential advantage in QUALM complexity of coherent over incoherent access QUALMs (even when the latter are adaptive), for two physically motivated problems. Moreover, this exponential advantage is achieved using an very simple coherent QUALM, based solely on the SWAP test.

At first glance, it might *seem* that early quantum black box algorithms such as Simon’s algorithm^33^ already provide an exponential advantage of coherent over incoherent QUALMs, even if not for physically motivated tasks. However, when viewed as a QUALM, Simon’s algorithm in fact falls within the incoherent QUALM framework. Indeed, Simon’s algorithm accesses each sample separately, uses product state preparations, and only utilizes measurements in a tensor product basis. However, recently, Simon’s algorithm was upgraded to a recursive version^34^, which was used to provide an example of an exponential separation between the computational power of quantum circuits (with access to a black box) of different depths. Another example of such a separation of the depth hierarchy was given in^35^. Interestingly, these results can be interpreted (with a little bit of translation work) into two other examples of exponential separations between coherent and incoherent QUALMs, albeit for tasks, which are quite contrived from a physics perspective.

It is also interesting to view related and previous works in the language of QUALMs, and compare to our work. Some previous results imply a quadratic advantage in QUALM complexity, and only under the strong assumption of non-adaptive access in the incoherent setting^36,37^. For other results an exponential lower bound is only conjectured (e.g., refs. ^38,39^). An example more closely related to our work is ref. ^40^, which provides a proven exponential advantage of coherent QUALMs over what we call “single register access” (which is a restricted case of incoherent QUALMs) for a quantum state distinction problem emerging from the dihedral hidden subgroup problem. However, importantly, the coherent protocol suggested has exponential gate complexity, and so the related experiment is not known to be efficient even in the coherent access setting (also, once again, the lower bound holds only under the strong non-adaptive assumption). The recent independent work of ref. ^41^ studied a closely related setting, and provided an exponential query complexity separation between incoherent and coherent QUALMs for a state tomography task, using quantum machine learning; however, as in ref. ^40^, the focus of ref. ^41^ is on query complexity; both their coherent and incoherent experiments have exponential gate complexity.

We gave the first evidence that entanglement and coherence could be truly exponentially advantageous (in terms of physical resources) when performing experiments in the lab, for physically motivated tasks. Our work suggests that coherence could be an immense resource in quantum experiments, and highlights the fact that quantum computers have a huge potential not only in speeding up the solution for computational problems, but also in providing dramatic savings in performing experimental tasks. In particular, important savings in resources may be achievable by using more sophisticated quantum algorithmic ideas, much beyond the SWAP test, which is used here.

Looking forward, we hope that the QUALM framework will be helpful in the study and development of new experimental techniques leveraging quantum computational components and ideas. Numerous interesting open questions are raised; for example, does adaptiveness help in the incoherent setting (see refs. ^42,43^)? How can more sophisticated quantum input states and measurement bases help in various experiments? How much does a larger work space buy us? Importantly, the examples we provided here lose their exponential advantage in the presence of noise. Can exponential advantages in QUALM complexity be exhibited in the NISQ era? More generally, can the advantages be achieved in settings closer to reality, e.g., where the lab oracles are efficient? It would be very interesting to experimentally demonstrate advantages of coherent quantum experiments.

## Methods

### Notations

We consider a total Hilbert space **H** = **N** ⊗ **L** ⊗ **W** (we use the same notation to denote the subsystems and their associated Hilbert spaces). **N** stands for the “Nature” Hilbert space of *n* qubits, to which the experimentalist has no direct access; The “lab” Hilbert space is denoted **L** and consists of *ℓ* qubits, corresponding to the degrees of freedom of the physical system, which the experimentalist can access, and **W**, a subsystem of *w* qubits, can be thought of as the “working space” of the experimentalist. The set of states (density matrices) on each subsystem will be denoted by ${{{{{\mathscr{D}}}}}}({{{{{\bf{N}}}}}}),{{{{{\mathscr{D}}}}}}({{{{{\bf{L}}}}}}),{{{{{\mathscr{D}}}}}}({{{{{\bf{W}}}}}}),...$; we similarly denote classical probability distributions on {0, 1}^*k*^ by ${{{{{\mathscr{D}}}}}}({\{0,1\}}^{k})$. We denote the Hilbert space of the union of two subsystems **L**, **W** (as well as the set of qubits) by **LW**, or **L** ⊗ **W**. Operators, superoperators, as well as sets thereof, will be denoted using a calligraphic font: ${{{{{\mathscr{E}}}}}},{{{{{\mathscr{Q}}}}}},{{{{{\mathscr{O}}}}}}$ etc. We will often abuse notation, and refer to an ordered sequence of superoperators ${{{{{\mathscr{Q}}}}}}=({{{{{{\mathscr{Q}}}}}}}_{1},{{{{{{\mathscr{Q}}}}}}}_{2},...,{{{{{{\mathscr{Q}}}}}}}_{k})$ as equal to the operator ${{{{{\mathscr{Q}}}}}}={{{{{{\mathscr{Q}}}}}}}_{k}\circ \cdots \circ {{{{{{\mathscr{Q}}}}}}}_{2}\circ {{{{{{\mathscr{Q}}}}}}}_{1}$, which is the result of applying the superoperators in the sequence in the given order.

### The QUALM framework

We now provide the definitions required for the QUALM framework; The setup is summarized in Fig. 1. Our first definition is that of the *lab oracle*; it models the input physical system on which the experiment is performed.

**Definition 6** (*Lab Oracle*). A lab oracle is specified by a pair ${\sf{LO}}=({{{{{{\mathscr{E}}}}}}}_{{{{{{\bf{NL}}}}}}},{\rho }_{{{{{{\bf{N}}}}}}})$ where ${{{{{{\mathscr{E}}}}}}}_{{{{{{\bf{NL}}}}}}}$ is a quantum superoperator (i.e. a completely positive trace-preserving map) on **N** ⊗ **L** and *ρ*_**N**_ is state on **N**. The set of lab oracles is denoted by ${{{{{\mathscr{L}}}}}}{{{{{\mathscr{O}}}}}}({{{{{\bf{N}}}}}},{{{{{\bf{L}}}}}})$.

Now we define the notion of a *task*, which corresponds to the “experimental problem” to be solved: it describes what it is that the experimentalist wants to measure. The experimentalist must achieve the task only by using the lab oracle superoperator, together with the operations at her disposal in her laboratory (i.e., the admissible gates on **L** ⊗ **W**).

**Definition 7** (*Admissible gates*). We denote by ${{{{{\mathscr{G}}}}}}$ a set of “admissible gates”, namely a set of quantum superoperators acting on **L** ⊗ **W**. (Note that superoperators include measurements).

**Definition 8** (*Task*). A ‘task’ is a tuple ${\sf{Task}}=({{{{{{\bf{S}}}}}}}_{{{\mbox{in}}}},{{{{{{\bf{S}}}}}}}_{{{\mbox{out}}}},f,{{{{{\mathscr{G}}}}}})$, associated with a given system **N** ⊗ **L** ⊗ **W** (which is usually implicit). Here, **S**_in_ is a *p*-qubit subsystem of **W**, **S**_out_ is a *q*-qubit subsystem of **W**, *f* is a function

3 $$f:\{{{\sf{LO}}}_{0},{{\sf{LO}}}_{1},{{\sf{LO}}}_{2},...\}\times {\{0,1\}}^{p}\longrightarrow {\{0,1\}}^{q}\ ,$$

and ${{{{{\mathscr{G}}}}}}$ is a set of admissible gates on **L** ⊗ **W**. In the domain of *f*, {LO_0_, LO_1_, LO_2_, . . . } is a set of lab oracles (here we denoted this set as discrete, but of course one can also consider a continuous set of lab oracles as input), i.e. a subset of ${{{{{\mathscr{L}}}}}}{{{{{\mathscr{O}}}}}}({{{{{\bf{N}}}}}},{{{{{\bf{L}}}}}})$.

This definition can be thought of as follows. Given a set of lab oracles that represent possible input physical systems, the task is to compute the function *f,* which takes as input a lab oracle, some classical lab settings (i.e., bit strings in {0, 1}^*p*^), and outputs a classical ‘experimental result’ (i.e., bit strings {0, 1}^*q*^). The task is to be achieved by constructing a circuit from admissible gates in ${{{{{\mathscr{G}}}}}}$, in conjunction with interspersed calls to the lab oracle superoperator. In many situations it is more natural to consider output *probability distributions* and define *f* to be

4 $$f:\{{{\sf{LO}}}_{0},{{\sf{LO}}}_{1},{{\sf{LO}}}_{2},...\}\times {\{0,1\}}^{p}\longrightarrow {{{{{\mathscr{D}}}}}}({\{0,1\}}^{q})\ .$$

Such is the case for classical sampling tasks, for continuous sets of input lab oracles (see example 2 in subsection 2.5 in Supplementary Information), as well as in the context of the task of distinguishing between lab oracles, which is the main focus of this paper; in this case, this generalized notion of a task in fact reduces to Definition 8.

We can now define a QUALM, which can be viewed as a specific choice of *protocol* for the execution of a Task.

**Definition 9** (QUALM) A QUALM over the set of admissible gates ${{{{{\mathscr{G}}}}}}$ acting on registers **L, W**, is an ordered sequence of symbols ${{{{{\mathscr{Q}}}}}}=({{{{{{\mathscr{Q}}}}}}}_{1},{{{{{{\mathscr{Q}}}}}}}_{2},...,{{{{{{\mathscr{Q}}}}}}}_{{{\mbox{final}}}})$ from the alphabet ${{{{{\mathscr{G}}}}}}\cup \{\square \}$, together with a specification of input and output subsystems **S**_in_, **S**_out_ ⊆ **W**. Here, we are treating ${{{{{\mathscr{G}}}}}}$ as a set of symbols (i.e., each gate labels a distinct symbol) and likewise □ is a symbol. Each QUALM has an associated map

5 $${\sf{QUALM}}:{{{{{\mathscr{L}}}}}}{{{{{\mathscr{O}}}}}}({{{{{\bf{N}}}}}},{{{{{\bf{L}}}}}})\longrightarrow {\sf{QuantumCircuits}}({{{{{\bf{N}}}}}}\otimes {{{{{\bf{L}}}}}}\otimes {{{{{\bf{W}}}}}})\ .$$

This function takes in a lab oracle LO, and outputs a quantum circuit on **N** ⊗ **L** ⊗ **W**. Specifically, QUALM(LO) ‘compiles’ a quantum circuit ${{{{{\mathscr{Q}}}}}}=({{{{{{\mathscr{Q}}}}}}}_{1},{{{{{{\mathscr{Q}}}}}}}_{2},...,{{{{{{\mathscr{Q}}}}}}}_{{{\mbox{final}}}})$ where each symbol in ${{{{{\mathscr{G}}}}}}$ is replaced by its corresponding gate, and each □ is replaced by the superoperator ${{{{{{\mathscr{E}}}}}}}_{{{{{{\bf{NL}}}}}}}$ corresponding to LO. **S**_in_, **S**_out_ correspond to the input and output subsystems of the resulting circuit, respectively.

In less formal terms, a QUALM is a quantum circuit built out of an admissible gate set, where the circuit has designated spots for a lab oracle superoperator to be inserted, and specified input and output qubits.

Now we explain what it means for a QUALM to achieve a particular Task. We first define the output density matrix of a QUALM for a given lab oracle LO. The idea is to compile the quantum circuit QUALM(LO) for the lab oracle LO, and then to use it to evaluate *f*(LO, *x*). To do so, we construct the initial state of the circuit to be (i) ${\rho }_{{{{{{\bf{N}}}}}}}^{{\sf{LO}}}$ (i.e. the state corresponding to the lab oracle LO) on **N**, (ii) $\left|x\right\rangle \left\langle x\right|$ on **S**_in_, and (iii) the zero state elsewhere. The full initial state will be denoted as ${\rho }_{{{{{{\bf{N}}}}}}}^{{\sf{LO}}}\otimes \left|x\right\rangle \langle x{| }_{{{{{{{\bf{S}}}}}}}_{{{\mbox{in}}}}}\otimes | {{{{{\bf{0}}}}}}\rangle \left\langle {{{{{\bf{0}}}}}}\right|$. We will run the initial state through the circuit QUALM(LO), and then trace out everything not in **S**_out_:

6 $${\rho }_{{{{{{{\bf{S}}}}}}}_{{{\mbox{out}}}}}({\sf{QUALM}}({\sf{LO}},x))={{{\mbox{tr}}}}_{{\overline{{{{{{\bf{S}}}}}}}}_{{{\mbox{out}}}}}\left\{{\sf{QUALM}}({\sf{LO}})\left[{\rho }_{{{{{{\bf{N}}}}}}}^{{\sf{LO}}}\otimes \left|x\right\rangle \langle x{| }_{{{{{{{\bf{S}}}}}}}_{{{\mbox{in}}}}}\otimes | {{{{{\bf{0}}}}}}\rangle \left\langle {{{{{\bf{0}}}}}}\right|\right]\right\}.$$

We note that a Task specifies a function *f*: {LO_0_, LO_1_, LO_2_, . . . } × {0, 1}^*p*^ ⟶ {0, 1}^*q*^. We say that the QUALM implements the Task with error at most *ϵ* if for each input (LO_*i*_, *x*), we have

7 $${\left\Vert {\rho }_{{{{{{{\bf{S}}}}}}}_{{{\mbox{out}}}}}({\sf{QUALM}}({{\sf{LO}}}_{i},x))-\left|f({{\sf{LO}}}_{i},x)\right\rangle \left\langle f({{\sf{LO}}}_{i},x)\right|\right\Vert }_{1}\le \epsilon .$$

Note that this definition reduces to stadard quantum algorithms, by letting the set of lab oracles in the domain be the empty set.

**Remark 1** (*Subroutines, Error reduction by repetition, Approximation*). We remark that standard manipulations from the theory of algorithms carry over to the QUALM setting in a natural way. In particular, a QUALM can be used as a subroutine by another QUALM, as long as they both act on the same lab register **L** (like in standard subroutines, we can decide which qubit “wires” to glue from the original QUALM to the input qubits of the subroutine QUALM, and similarly for the outputs).

Using such subroutine QUALMs, one can achieve error reduction (also known as amplification); this is a standard primitive in probabilistic algorithms^44^, which is also needed in this work. This is done by a straightforward generalization of the way it is done for algorithms. For example, suppose we want to reduce the error probability of a given QUALM, which achieves a certain deterministic task with error 1/3; and further suppose that the image of the function *f* has a single output bit, measured at the end of the QUALM in the computational basis. One can construct a new ${\sf{QUALM}}^{\prime}$, which first copies the *p*-bit input string *xm* times (for some desired amplification parameter *m*). It then applies QUALM as a subroutine *m* times, each time with a new set of qubits initialized to 0 (which together with the *p* qubits containing the appropriate copy of *x*, constituting the working register **W** of the particular subroutine). ${\sf{QUALM}}^{\prime}$ then applies a majority calculation on all outputs of the *m* subroutines; this majority is the output bit of the ${\sf{QUALM}}^{\prime}$.

In the same manner, we can consider amplifications for QUALMs, which compute probabilistic functions. If for two different lab oracles the output distributions are *δ* apart in total variation distance, one can amplify the distance by repetition and classical postprocessing.

### QUALM complexity

Having defined tasks and QUALMs, we now turn to defining QUALM complexity.

**Definition 10** (*Gate complexity, query complexity, and QUALM complexity*). The gate complexity of a given QUALM over the admissible set of gates ${{{{{\mathscr{G}}}}}}$ is the length (i.e. the number of symbols from ${{{{{\mathscr{G}}}}}}\cup \{\square \}$) of the sequence ${{{{{\mathscr{Q}}}}}}$, minus the number of □ symbols. We denote this by GateComplexity[QUALM], and call this the QUALM gate complexity. Similarly, the query complexity is the number of □’s appearing in ${{{{{\mathscr{Q}}}}}}$, and this is denoted by QueryComplexity[QUALM]. This is called the QUALM query complexity. We call the sum

8 $${\sf{GateComplexity}}[{\sf{QUALM}}]+{\sf{QueryComplexity}}[{\sf{QUALM}}]=| {{{{{\mathscr{Q}}}}}}| $$

the QUALM complexity.

The exact (respectively, approximate) QUALM complexity of a task is given by the QUALM with least QUALM complexity, which achieves the task exactly (respectively, approximately).

We also note that it might be relevant, in various situations, to weight gates versus query calls differently, namely to consider the QUALM complexity to be

9 $${\sf{GateComplexity}}[{\sf{QUALM}}]+\lambda \ {\sf{QueryComplexity}}[{\sf{QUALM}}]$$

for some suitable penalty factor *λ*.

As usual in computational complexity^45^, one is interested in *families* of tasks and QUALMs, where some parameter dictating the *size* of the problem grows to infinity, and we ask how the complexity grows as a function of that parameter.

### Different types of access to a lab oracle

Towards clarifying the power of different QUALMs in terms of their computational abilities, we consider different types of *accesses* of QUALMs to a lab oracle. The most natural (though presently least realistic) one is the access of a full quantum computer. By this we mean a general QUALM, without the restrictions to be specified shortly. In particular, the set of admissible gates ${{{{{\mathscr{G}}}}}}$ can be a universal set, and there is no restriction on which gates can be applied at any given time.

**Definition 11** (*Coherent access*). We will refer to a general QUALM (i.e., with a universal gate set) defined in Definition 9 as a QUALM with *coherent access* to the lab oracle.

In realistic physics experiments, access is often far more limited. The experimental setup may not be able to introduce quantum entanglement between the physical system **N** ⊗ **L** and the rest of the lab **W**. Measurements to the system may destroy the quantum coherence in the physical system or even completely destroy the quantum state. In the QUALM framework this is captured by restrictions on the admissible gate sets and the allowed sequences of gates. To make concrete progress we consider a model of many contemporary experiments, which we call *incoherent access*. To this end, we need to recall the definition of a one-round LOCC protocol between two parties (see, e.g., ^46^). First, we recall that a map ${{{{{{\mathscr{N}}}}}}}^{{{{{{\bf{R}}}}}}}$ on density matrices on the register **R** is completely positive (CP for short) if it can be associated with a set of Kraus operators ${\{{{{{{{\mathscr{A}}}}}}}_{\alpha }\}}_{\alpha }$ such that for all *ρ* on **R**

10 $${{{{{{\mathscr{N}}}}}}}^{{{{{{\bf{R}}}}}}}(\rho )=\mathop{\sum}\limits_{\alpha }{{{{{{\mathscr{A}}}}}}}_{\alpha }\rho {{{{{{\mathscr{A}}}}}}}_{\alpha }^{{{\dagger}} }\ .$$

Furthermore, ${{{{{{\mathscr{N}}}}}}}^{{{{{{\bf{R}}}}}}}$ is trace-preserving if

11 $$\mathop{\sum}\limits_{\alpha }{{{{{{\mathscr{A}}}}}}}_{\alpha }^{{{\dagger}} }{{{{{{\mathscr{A}}}}}}}_{\alpha }={\mathbb{1}}\ .$$

A completely positive trace-preserving (CPTP) map is often called, simply, a quantum channel.

Now we specify a quantum channel that implements a certain kind of communication between subsystems **A** and **B** called a “one-round LOCC” (see^46^).

**Definition 12** (*One-round LOCC*. A one-round LOCC operator from subsystems **A** to **B** is a quantum channel (i.e. a CPTP map) ${{{{{{\mathscr{E}}}}}}}^{{{{{{\bf{AB}}}}}}}$ acting on ${{{{{\mathscr{D}}}}}}({{{{{\bf{A}}}}}}\otimes {{{{{\bf{B}}}}}})$, which is of the form

12 $${{{{{{\mathscr{E}}}}}}}^{{{{{{\bf{AB}}}}}}}(\cdot )=\mathop{\sum}\limits_{\alpha }{{{{{{\mathscr{M}}}}}}}_{\alpha }^{{{{{{\bf{A}}}}}}}(\cdot )\otimes {{{{{{\mathscr{N}}}}}}}_{\alpha }^{{{{{{\bf{B}}}}}}}(\cdot )$$

with ${{{{{{\mathscr{M}}}}}}}_{\alpha }^{{{{{{\bf{A}}}}}}}$ a completely positive (CP) map acting on the **A** subsystem, and ${{{{{{\mathscr{N}}}}}}}_{\alpha }^{{{{{{\bf{B}}}}}}}$ a completely positive and trace-preserving (CPTP) map acting on the **B** subsystem.

It should be noted that this definition in fact requires ${{{{{{\mathscr{E}}}}}}}^{{{{{{\bf{A}}}}}}}\equiv {\sum }_{\alpha }{{{{{{\mathscr{M}}}}}}}_{\alpha }^{{{{{{\bf{A}}}}}}}$ to be a CPTP map (instead of merely a CP map), since ${{{{{{\mathscr{E}}}}}}}^{{{{{{\bf{A}}}}}}}$ is the reduced channel obtained by tracing over **B** in ${{{{{{\mathscr{E}}}}}}}^{{{{{{\bf{AB}}}}}}}$.

With the above definition in mind, we can now define incoherent access QUALMs:

**Definition 13** (*Incoherent access*). We say a QUALM uses *incoherent access* to the lab oracle if the following holds for the sequence of symbols ${{{{{\mathscr{Q}}}}}}$. Let *k* be the number of times the symbol □ appears in ${{{{{\mathscr{Q}}}}}}=({{{{{{\mathscr{Q}}}}}}}_{1},{{{{{{\mathscr{Q}}}}}}}_{2},...,{{{{{{\mathscr{Q}}}}}}}_{{{\mbox{final}}}})$. As usual, we associate ${{{{{\mathscr{Q}}}}}}$ with the channel ${{{{{\mathscr{Q}}}}}}={{{{{{\mathscr{Q}}}}}}}_{{{\mbox{final}}}}\circ \cdots \circ {{{{{{\mathscr{Q}}}}}}}_{2}\circ {{{{{{\mathscr{Q}}}}}}}_{1}$, and regroup terms as

13 $${{{{{\mathscr{Q}}}}}}={{{{{{\mathscr{C}}}}}}}_{k}\circ \square \circ {{{{{{\mathscr{C}}}}}}}_{k-1}\circ \square \circ \cdots \circ \square \circ {{{{{{\mathscr{C}}}}}}}_{0}$$

where ${{{{{{\mathscr{C}}}}}}}_{i}$ is a the sequence of gates applied after the *i*th call to the lab oracle, and before the (*i* + 1)st one. We require that

- For each *i* ∈ {0, . . . , *k*}, the sequence of admissible gates ${{{{{{\mathscr{C}}}}}}}_{i}$ can be written as an *r*_*i*_-round LOCC protocol, for some finite number of rounds *r*_*i*_. In other words, ${{{{{{\mathscr{C}}}}}}}_{i}$ can be written as a composition ${{{{{{\mathscr{C}}}}}}}_{i}={{{{{{\mathscr{C}}}}}}}_{i,{r}_{i}}\circ \cdots \circ {{{{{{\mathscr{C}}}}}}}_{i,2}\circ {{{{{{\mathscr{C}}}}}}}_{i,1}$ such that for every *j*, ${{{{{{\mathscr{C}}}}}}}_{i,j}$ is a one-round LOCC channel. Without losing generality, we can assume ${{{{{{\mathscr{C}}}}}}}_{i,j}$ alternates between **L**-to-**W** and **W**-to-**L** one-round LOCC channels, since the composition of two **L**-to-**W** one-round LOCC’s is still a one-round LOCC.
- Moreover we also require that for each *i* there exists at least one index *j* ∈ {1, . . . , *r*_*i*_} such that ${{{{{{\mathscr{C}}}}}}}_{i,j}$ is a complete measurement, which is a special one-round LOCC operator from **L** to **W**, as follows:
  14 $${{{{{{\mathscr{C}}}}}}}_{i,j}(\cdot )=\mathop{\sum}\limits_{\alpha \in {\{0,1\}}^{\ell }}{{{{{{\mathscr{M}}}}}}}_{\alpha }^{{{{{{\bf{L}}}}}}}(\cdot )\otimes {{{{{{\mathscr{N}}}}}}}_{\alpha }^{{{{{{\bf{W}}}}}}}(\cdot ),$$
  such that ${{{{{{\mathscr{M}}}}}}}_{\alpha }^{{{{{{\bf{L}}}}}}}$ is a rank one projection for each *α*, i.e., ${{{{{{\mathscr{M}}}}}}}_{\alpha }^{{{{{{\bf{L}}}}}}}(\rho )=\left|{\psi }_{\alpha }\right\rangle \left\langle {\psi }_{\alpha }\right|\left\langle {\psi }_{\alpha }\right|\rho \left|{\psi }_{\alpha }\right\rangle$ for some pure state $\left|{\psi }_{\alpha }\right\rangle$, and ${\{\left|{\psi }_{\alpha }\right\rangle \}}_{\alpha \in {\{0,1\}}^{\ell }}$ is an orthonormal basis for **L**.

The above definition roughly means that the interaction between the **L** and **W** registers is an LOCC throughout the QUALM protocol; moreover, between any two applications of the lab oracle, the lab register **L** is measured using a complete measurement. No coherence can be generated between the state generated by a given single call to the lab oracle, and any other register used by the QUALM – this is the source for the term “incoherent access QUALM”.

We note that the above definition allows *adaptive* access to the lab oracle; namely, the state of the register **L** before an application of the lab oracle may depend on previous measurement results both of **L** and of **W**, and those in turn may depend on the lab oracle.

### Sketch of the proof of Theorem 1

The proof of Theorem 1 consists of two steps. The first step is to study a special case, which we call a ‘simple measurement’ (SM) QUALM, and show that it cannot distinguish the two lab oracles LO_1_(*ℓ*), LO_0_(*ℓ*) . The second step is to show that the output of a general incoherent access QUALM can be related to a probabilistic average of those SM QUALMs, so that a general incoherent access QUALM cannot do better than a SM QUALM. In the following we will first define the SM QUALM and sketch the proof for this special case, and then discuss how the general incoherent QUALM is reduced to SM QUALM.

**Fig. 2:**
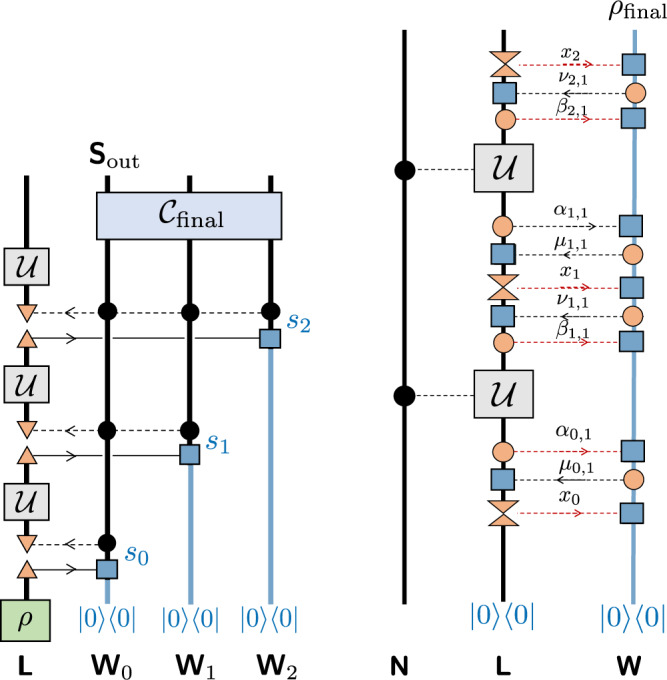
Circuit for a simple measurement QUALM. (Left) Illustration of the simple measurement QUALM defined in Definition 14. The upward pointing triangles indicate the weighted projection ${\rho }_{{{{{{\bf{L}}}}}}}\to \langle {y}_{{s}_{0},...{s}_{i}}|{\rho }_{{{{{{\bf{L}}}}}}}|{y}_{{s}_{0},...,{s}_{i}}\rangle {\lambda }_{{s}_{0},...,{s}_{i}}$. The horizontal solid lines indicate the recording of POVM measurement results in **W**_*i*_. The horizontal dashed lines connected to downward pointing triangles indicate the preparation of initial state ${\sigma }_{{s}_{0},...,{s}_{i}}$ controlled by previous measurement results *s*_0_, . . . , *s*_*i*_. (Right) Illustration of the incoherent access QUALM defined in Definition 13. Each orange solid circle is a CP map and each blue box is a CPTP map. At least one of the CP maps between each application of the lab oracle is a complete measurement, indicated by the double triangle. The direction of the arrow in each horizontal dashed line indicates the direction of classical information flow. Only the LOCC’s corresponding to *β*_*i*,*j*_ and *x*_*i*_ lead to conditional probabilities that depend on the lab oracle, which are indicated by red dashed lines.

**Definition 14** (Roughly) Single measurement QUALM. The SM QUALM is illustrated in Fig. 2. It describes a QUALM where there is only one measurement carried out after each application of lab oracle. The measurements are of a particular form: each is a POVM in which each element is of rank one. In the *i*th round, the measurement output *s*_*i*_ is recorded in a new tensor factor of **W**, denoted by **W**_*i*_ . The *i*th POVM is thus described by

15 $${\{{\lambda }_{{s}_{0}{s}_{1}...{s}_{i}}^{i}\left|{y}_{{s}_{0}{s}_{1}...{s}_{i}}^{i}\right\rangle \left\langle {y}_{{s}_{0}{s}_{1}...{s}_{i}}^{i}\right|\}}_{{s}_{i}},\ \,{{\mbox{where}}}\,\ \mathop{\sum}\limits_{{s}_{i}}{\lambda }_{{s}_{0}{s}_{1}...{s}_{i}}^{i}\left|{y}_{{s}_{0}{s}_{1}...{s}_{i}}^{i}\right\rangle \left\langle {y}_{{s}_{0}{s}_{1}...{s}_{i}}^{i}\right|={\mathbb{1}},\ \ \,0 \, < \, {\lambda }_{{s}_{0}{s}_{1}...{s}_{i}}^{i}\le 1\ .$$

We note that both $\left|{y}_{{s}_{0}{s}_{1}...{s}_{i}}^{i}\right\rangle$ and ${\lambda }_{{s}_{0}{s}_{1}...{s}_{i}}^{i}$ depend not only on *s*_*i*_ but also on all previous measurement results *s*_0_, . . . , *s*_*i*−1_ or in short, *s*_*j*<*i*_ . After the measurement, **L** is prepared into a mixed state ${\sigma }_{{s}_{0}{s}_{1}...{s}_{i}}^{i}$, which again can depend on the previous measurement results *s*_1_, . . . , *s*_*i*−1_. After the last measurement, a readout channel ${{{{{{\mathscr{C}}}}}}}_{{{\mbox{out}}}}$ is applied to **W**, which maps the diagonal density operator of **W** to a single qubit output state.

For the SM QUALM, the output density operator is determined by applying the readout channel ${{{{{{\mathscr{C}}}}}}}_{{{\mbox{final}}}}$ to the diagonal density operator of **W**, which encodes the probability distribution of the measurement results *s*_0_, *s*_1_, . . . , *s*_*k*_. For the fixed random unitary lab oracle LO_0_(*ℓ*) (Definition 4), the probability distribution is

16 $${Q}_{k}\left({s}_{0},{s}_{1},...,{s}_{k}\right)=\,{{\mbox{Pr}}}\,({s}_{0})\cdot \left(\int _{{{{{{\rm{Haar}}}}}}}dU\mathop{\prod }\limits_{i=1}^{k}\left\langle {y}_{{s}_{0}{s}_{1}...{s}_{i}}^{i}\right|U{\sigma }_{{s}_{0}{s}_{1}...{s}_{i-1}}^{i-1}{U}^{{{\dagger}} }\left|{y}_{{s}_{0}{s}_{1}...{s}_{i}}^{i}\right\rangle {\lambda }_{{s}_{0}{s}_{1}...{s}_{i}}^{i}\right)$$

with $\,{{\mbox{Pr}}}\,({s}_{0})=\left|\left\langle {y}_{{s}_{0}}^{0}\right|{0}^{{{{{{\bf{L}}}}}}}\right\rangle {| }^{2}{\lambda }_{{s}_{0}}^{0}$. The probability distribution for the other lab oracle LO_1_(*ℓ*) (Definition 4) is

17 $${P}_{k}\left({s}_{0},{s}_{1},...,{s}_{k}\right)=	\,{{\mbox{Pr}}}\,({s}_{0})\left(\mathop{\prod }\limits_{i=1}^{k}\int _{{{{{{\rm{Haar}}}}}}}d{U}_{i}\left\langle {y}_{{s}_{0}{s}_{1}...{s}_{i}}^{i}\right|U{\sigma }_{{s}_{0}{s}_{1}...{s}_{i-1}}^{i-1}{U}^{{{\dagger}} }\left|{y}_{{s}_{0}{s}_{1}...{s}_{i}}^{i}\right\rangle {\lambda }_{{s}_{0}{s}_{1}...{s}_{i}}^{i}\right)\\ =	\,{{\mbox{Pr}}}\,({s}_{0}){D}^{-k}\mathop{\prod }\limits_{i=1}^{k}{\lambda }_{{s}_{i}}^{i}.$$

In the following we will often denote the ordered sequence *s*_0_, *s*_1_, . . . , *s*_*k*_ by *s* for simplicity.

Our conclusion is that these two probability distributions *Q*_*k*_ and *P*_*k*_ are difficult to distinguish. More precisely, for $k \, < \, {\left({2}^{\ell }/\sqrt{6}\right)}^{4/7}$, we will prove

18 $$\parallel {P}_{k}-{Q}_{k}{\parallel }_{1}=\mathop{\sum}\limits_{s}\left|{P}_{k}\left(s\right)-{Q}_{k}\left(s\right)\right|\le {{{{{\mathscr{O}}}}}}\left(\frac{{k}^{3}}{{2}^{\ell }}\right)\ .$$

The key mathematical tool we use is the Weingarten functions of the unitary group:

19 $$\int _{{{{{{\rm{Haar}}}}}}}dU{\left[{U}^{\otimes k}\right]}_{IJ}{\left[{U}^{* \otimes k}\right]}_{KL}=\mathop{\sum}\limits_{\sigma ,\tau \in {S}^{k}}{\tau }_{KI}{\sigma }_{LJ}W(\tau {\sigma }^{-1},D)\ .$$

Here *I*, *J*, *K*, *L* label an orthonormal basis in the *k*-copied Hilbert space. The action of the permutation group elements *σ* and *τ* corresponds to the permutation of different Hilbert space copies. Using Eqn. (19), *Q*_*k*_ in Eq. (16) can be rewritten as

20 $${Q}_{k}(s)=\mathop{\sum}\limits_{\sigma ,\tau \in {S}^{k}}\,{{{{\mathrm{tr}}}}}\,({A}_{s}\sigma )\,{{{{\mathrm{tr}}}}}\,({B}_{s}{\tau }^{-1})W(\tau {\sigma }^{-1},D)\ .$$

with ${A}_{s}{ = \bigotimes }_{i = 1}^{k}{\sigma }_{{s}_{0}{s}_{1}...{s}_{i-1}}^{i-1}, {B}_{s}{ = \bigotimes }_{i = 1}^{k}|{y}_{{s}_{0}{s}_{1}...{s}_{i}}^{i}\rangle \langle {y}_{{s}_{0}{s}_{1}...{s}_{i}}^{i}|{\lambda }_{{s}_{0}{s}_{1}...{s}_{i}}^{i}$. The sum in Eqn. (20) consists of three kinds of terms: (i) $\tau =\sigma ={\mathbb{1}}$; (ii) $\tau ={\mathbb{1}},\,\sigma \ne {\mathbb{1}}$; (iii) $\tau \ne {\mathbb{1}}$. This leads to the following inequality:

21 $$\left|{Q}_{k}(s)-{P}_{k}(s)\right|\le 	\, \left|W({\mathbb{1}},D)-{D}^{-k}\right|{{{{{\mathrm{tr}}}}}}({B}_{s})+\mathop{\sum}\limits_{\sigma \ne {\mathbb{1}}}\left|W({\sigma }^{-1},D)\right|\left|{{{{{\rm{tr}}}}}}({A}_{s}\sigma )\right|{{{{{\rm{tr}}}}}}({B}_{s})\\ 	\, +\mathop{\sum}\limits_{\tau \ne {\mathbb{1}}}\mathop{\sum}\limits_{\sigma }\left|W(\tau {\sigma }^{-1},D)\right|\left|{{{{{\rm{tr}}}}}}({A}_{s}\sigma )\right|\left|{{{{{\rm{tr}}}}}}({B}_{s}{\tau }^{-1})\right|$$

22 $$\le \left[\left|W({\mathbb{1}},D)-{D}^{-k}\right|+\mathop{\sum}\limits_{\nu \ne {\mathbb{1}}}\left|W(\nu ,D)\right|\right]{{{{{\rm{tr}}}}}}({B}_{s})+\mathop{\sum}\limits_{\nu }\left|W(\nu ,D)\right|\mathop{\sum}\limits_{\tau \ne {\mathbb{1}}}\left|{{{{{\rm{tr}}}}}}\left({B}_{s}{\tau }^{-1}\right)\right|\ .$$

In the second step we have used $\left|{{{{{\rm{tr}}}}}}({A}_{s}\sigma )\right|\le 1$. Carrying the sum over *s* and using known properties of the Weingarten function^47^, for $k \, < \, {\left({2}^{\ell }/\sqrt{6}\right)}^{4/7}$ we obtain

23 $$\delta \left({P}_{k},{Q}_{k}\right)\equiv \mathop{\sum}\limits_{s}\left|{Q}_{k}(s)-{P}_{k}(s)\right|\le {c}_{1}+{c}_{2}T$$

with

24 $$T=\frac{1}{{D}^{k}}\mathop{\sum}\limits_{\tau \ne {\mathbb{1}}}\mathop{\sum}\limits_{s}\left|{{{{{\rm{tr}}}}}}\left({B}_{s}{\tau }^{-1}\right)\right|$$

and coefficients ${c}_{1}={{{{{\mathscr{O}}}}}}\,\left(\frac{{k}^{7/2}}{{D}^{2}}\right)$, ${c}_{2}=1+{{{{{\mathscr{O}}}}}}\,\left(\frac{{k}^{2}}{D}\right)$. The remaining task is to bound *T*. *T* contains a product of matrix elements of the form

25 $${M}_{ji}=\left\langle \left.{y}_{{s}_{0}{s}_{1}...{s}_{j}}^{j}\right|{y}_{{s}_{0}{s}_{1}...{s}_{i}}^{i}\right\rangle $$

For example, for *k* = 8, *τ* = (175462)(3)(8) (which maps 175462 cyclically to 754621 and preserves 3, 8), we have $\left|{{{{{\rm{tr}}}}}}\left({B}_{s}{\tau }^{-1}\right)\right|=\left|{M}_{71}{M}_{57}{M}_{45}{M}_{64}{M}_{26}{M}_{12}\right|\mathop{\prod }\nolimits_{i = 1}^{8}{\lambda }_{{s}_{0}{s}_{1}...{s}_{i}}$. Because of the absolute value, we cannot directly carry the summation over *s*. However, we can decompose this string into two segments and use the simple inequality 2∣*ab*∣ ≤ ∣*a*∣^2^ + ∣*b*∣^2^. By carefully choosing the segments, we prove that the sum over *s* can now be carried using the completeness condition, and the adaptiveness does not cause a problem because one can always start the sum from the latest index *s*_*k*_. Using this method we obtain

26 $$T\le \frac{{k}^{3}}{D}+\frac{{k}^{2}}{D}+{{{{{\mathscr{O}}}}}}\,\left(\frac{{k}^{5}}{{D}^{2}}\right)\ .$$

Using Eq. (23) this proves the bound (18) with *c*_2_*T* the dominant term.

The remaining task is to prove that a general incoherent QUALM (Fig. 2(b)) cannot do better than SM QUALM. Here we will only provide an intuitive explanation of the idea, leaving more details in the Supplementary Information. In a general incoherent QUALM, multiple rounds of classical communication occur between **L** and **W** between two applications of the lab oracle. If we consider a case when the communication is only one way from **L** to **W**, then it is equivalent to a sequence of weak measurements, followed by a projective measurement in a complete basis. One can always consolidate all these measurements into a single POVM that is in the form of the one we have in SM QUALM. The problem becomes more nontrivial because there are communication from **W** to **L**, which measures the state of **W**, and tells **L** to apply a quantum channel determined by the measurement output. More precisely, the measurement corresponds to a family of CP maps ${{{{{{\mathscr{M}}}}}}}_{\nu }^{{{{{{\bf{W}}}}}}}$. For a state *ρ*_**W**_, the measurement output *ν* has probability ${p}_{\nu }={{{{{\rm{tr}}}}}}\left({{{{{{\mathscr{M}}}}}}}_{\nu }^{{{{{{\bf{W}}}}}}}\!\left({\rho }_{{{{{{\bf{W}}}}}}}\right)\right)$. When the measurement output is *ν*, a quantum channel ${{{{{{\mathscr{N}}}}}}}_{\nu }^{{{{{{\bf{L}}}}}}}$ is applied to **L**. The key idea is that this procedure can always be replaced by a deterministic (i.e., classical) computation based on previous measurement results and some random numbers. In other words, even if **W** is a quantum computer, since it is only allowed to send classical information to **L**, it can be simulated by a classical computer with random number generators. Consequently, we can replace all **W** to **L** one-way LOCC by deterministic instructions with random number inputs. Then for fixed values of these random numbers, what happens to **L** is just some quantum channels applied between weak measurements. In this case the consolidation can be done to reduce the QUALM to an SM QUALM. This establishes that a general incoherent QUALM is equivalent to a classical probabilistic average over SM QUALMs. Since the probabilistic average cannot perform better than the best SM QUALM, we have proved Theorem 1.

## Supplementary information

Supplementary Information

## Data Availability

No data was collected for this work.
