# Supplementary material for: Quantum algorithmic measurement
Source: Nat Commun. 2022 Feb 16;13:887. doi: 10.1038/s41467-021-27922-0 (PMC8850572; doi:10.1038/s41467-021-27922-0)
Supplement: Supplementary file 1 — Supplementary Information [file 41467_2021_27922_MOESM1_ESM.pdf]

Supplementary Information

Supplementary Note 1: Overview

Supplementary Note 1.1: A computational complexity point of view on quantum experiments

Our starting point is this: we can view a physical experiment, very roughly, as a generalization of an algorithm. An algorithm (classical or quantum) computes a function whose argument is a *classical* input string (e.g. computing the permanent for a matrix given as a bit string). In a quantum experiment, we can view the *input* to the process as a *quantum system* (a state, a quantum process, or both) to which we have some form of oracle access in the lab. The goal of a quantum experiment is to implement a function mapping (very roughly) $\{\text{physical quantum system}\} \rightarrow \{\text{classical bit string}\}$. Thus to define a quantum experiment, we turn to the insights underlying the theory of quantum algorithms.

Underlying the theory of quantum computation is the *quantum Church-Turing thesis*[15]. The thesis states that *any quantum physical process* can be viewed as a quantum information process, or more precisely, can be simulated efficiently by a quantum algorithm consisting of local gates and local measurements[15]. The thesis is widely conjectured to be true, and has been verified in many contexts, see e.g.[60]. We thus first make a simple but crucial proposition: In the most general quantum experiments, the experimentalist can in principle apply arbitrarily complicated quantum computations (by applying quantum gates) during the experiment. For example, she can prepare many copies of the physical system and entangle them with one another, pre- and post-process results of intermediate measurements using a quantum computing register on the side, and so on. We would thus like to model the physical experimental process as some sort of a quantum *algorithm*, composed of *quantum gates*. By the quantum Church-Turing thesis, this will indeed capture the most general possible quantum processes that the experimentalist controls, putting aside the details of implementation.

Of course, full-fledged quantum computers are not yet available, but our goal is to develop a *theory* of the most general quantum experiments which are in principle possible in the quantum world, so that we can study their limitations and possibilities. As we will see later in the paper, starting from such a general model, we can then rigorously define and study various interesting restrictions. We can choose these restricted variants to be close to the physical reality we want to understand.

The computational point of view on quantum experiments naturally enables us to define the *computational complexity of an experiment*: this will be the number of elementary, local quantum operations (e.g. gates or measurements) required to implement the quantum experiment. Thus we will arrive at a characterization of the complexity of quantum experiments in a manner *independent* of the physical systems and apparatuses by which they are instantiated (in direct parallel to the independence of the complexity of quantum algorithms from the exact details of implementation). This approach enables the study of quantum experiments from an abstract, mathematical point of view, independent of exact physical details. This is exactly our purpose. We will next define these abstract experimental protocols in detail, and call them *quantum algorithmic measurements*, or QUALMs.

Supplementary Note 1.2: QUALMs and lab oracles

To provide a definition of this model, let us consider first the example of an X-ray diffraction experiment, which is the standard way to determine the crystal structure of a material (see Figure 1; our diagrammatic notation is explained in detail in A). The experiment consists of (i) the crystal sample, (ii) the X-ray photons which exhibit electromagnetic interactions with the crystal, and (iii) the camera and other equipment used to record the data, which only interact with the X-ray photons (and not the material being imaged).

This is a very general situation: the experimentalist never has access to all degrees of freedom in the physical system she wants to measure. This is reminiscent of yet another model from theoretical computer science – that of interactive protocols[7]. A physical experiment is a kind of interaction between Nature and the experimental apparatus, but this interaction is only allowed access to some subset of the degrees of freedom characterizing the physical system.

To model such an experimental system, we will make use of three subsystems (registers). We will call the first subsystem "Nature" and denote it by N; we view it as the register that Nature holds secretly, and we have no direct access to it. This system has an associated Hilbert space which we denote by the same notation N. In the above example, N contains the crystal. Our experimental probe which couples to N is contained in what we call the lab system L; in the

above example, this contains the X-ray photons. We additionally have access to a working space system $\mathbf{W}$ which we can leverage to perform processing of our quantum data; in the above example, this contains the camera and all other instruments which process the date in the lab. In totality, the full Hilbert space is $\mathbf{H} = \mathbf{N} \otimes \mathbf{L} \otimes \mathbf{W}$ corresponding to the decomposition into subsystems $\mathbf{N}, \mathbf{L}, \mathbf{W}$. The basic idea is that we can make measurements to read out information from $\mathbf{L}$ and $\mathbf{W}$, but not directly from $\mathbf{N}$.

With this picture in mind, we delve into informal definitions for the QUALM framework:

Definition 1. (Roughly) A lab oracle is described by a pair $\mathsf{LO}(\mathbf{N}, \mathbf{L}) = (\mathscr{E}_{\mathbf{NL}}, \rho_{\mathbf{N}})$, where $\mathscr{E}_{\mathbf{NL}}$ is a superoperator acting jointly on $\mathbf{N}$ and $\mathbf{L}$, and $\rho_{\mathbf{N}}$ is the initial state of the $\mathbf{N}$ system. The idea is that $\mathscr{E}_{\mathbf{NL}}$ implements a coupling between our system $\mathbf{N}$ of study, and our lab $\mathbf{L}$. In other words, $\mathscr{E}_{\mathbf{NL}}$ implements the action of apparatus which allows the lab $\mathbf{L}$ to interact with the system $\mathbf{N}$ being studied.

The lab oracle models both the memory of the physical system, stored in $\mathbf{N}$, as well as the (generally, unknown) superoperator which the physical system applies.

Definition 2. (Roughly) A $\mathsf{QUALM}(\mathbf{N},\mathbf{L},\mathbf{W})$ is a specification of a quantum algorithm on the subsystems $\mathbf{N}, \mathbf{L}, \mathbf{W}$, involving the lab oracle superoperator. The algorithm consists of a sequence of gates (taken from the repertoire available in the lab) acting on the registers $\mathbf{L}, \mathbf{W}$, as well as a special step which corresponds to a query to the "lab oracle superoperator" acting on the registers $\mathbf{N}, \mathbf{L}$. Some of the qubits in the register $\mathbf{W}$ are marked as "inputs" and others are marked as "outputs".

Notice that in addition to the lab oracle, to which the QUALM is only given access as a black box and which can be thought of as a kind of quantum "input" to the QUALM, we also give the QUALM an additional bit string which is its classical input. This bit string can include, for example, a specification of certain parameters characterizing the experiment to be performed. We are also free to choose there to be no classical inputs (i.e., the input subset of $\mathbf{W}$ is the empty set). Note that the physical system $\mathbf{N}$ is treated as a black box, since we are not allowed to access the register $\mathbf{N}$ directly. A QUALM can thus be thought of as a quantum circuit that maps (i) a quantum system to which it has only indirect access, as well as (ii) an additional classical "input" bit string on a subset of $\mathbf{W}$, to a classical "output" bit string on $\mathbf{W}$, which is the outcome of the experiment. The purpose of applying a QUALM is to gain knowledge about the lab oracle that was previously unknown to the experimentalist. We will formalize an experimental problem as a 'Task', which is what a QUALM is designed to achieve.

The resulting model is a hybrid of two important models in the theory of quantum computation – that of quantum algorithms with (quantum) black boxes[76], and that of quantum interactive protocols[64] (see Subsection 1.5 for further discussion). Note that the sequential interactions between $\mathbf{L}$ and $\mathbf{W}$ can leverage entanglement, and be adaptive; we have no (direct) control over the interaction between $\mathbf{L}$ and $\mathbf{N}$. Thus an experiment is modeled in our framework as an interactive protocol with Nature, about which we want to learn something (see Figure 1). The complexity of a QUALM is naturally the number of calls to the lab oracle, plus the number of gates applied on $\mathbf{L}$, $\mathbf{W}$. This is a combination of both query complexity and gate complexity, and we will discuss this further below.

The many examples of quantum experiments mentioned above can indeed be described in the QUALM model; we demonstrate this for some examples in Subsection 2.5.

Supplementary Note 1.3: Exponential advantage from quantum coherence

In physics experiments, it is clear that the complexity/difficulty of a task depends on the experimental setup. For example, measuring the size of a vortex in a two-dimensional superconductor is much easier for a scanning tunneling microscope (STM) than for a standard charge transport experiment, because the former has a much higher spatial resolution. The framework of QUALMs allows us to precisely define the capability of an experiment, and study the complexity of a task with respect to that.

In this work, we are interested in particular in the role of quantum entanglement in the complexity of QUALMs. In many physics experiments, the experimental setup is not capable of introducing or manipulating quantum entanglement between the lab system $\mathbf{L}$ and the working space $\mathbf{W}$. For example, in a standard X-ray diffraction experiment, the photon source does not have the ability to create EPR pairs of photons. Even if an outgoing photon might in general be in a quantum entangled state with the crystal sample in $\mathbf{N}$, the outgoing photons are measured by a camera, after which we gain classical information about the photons' locations, and all other information about their quantum coherence is

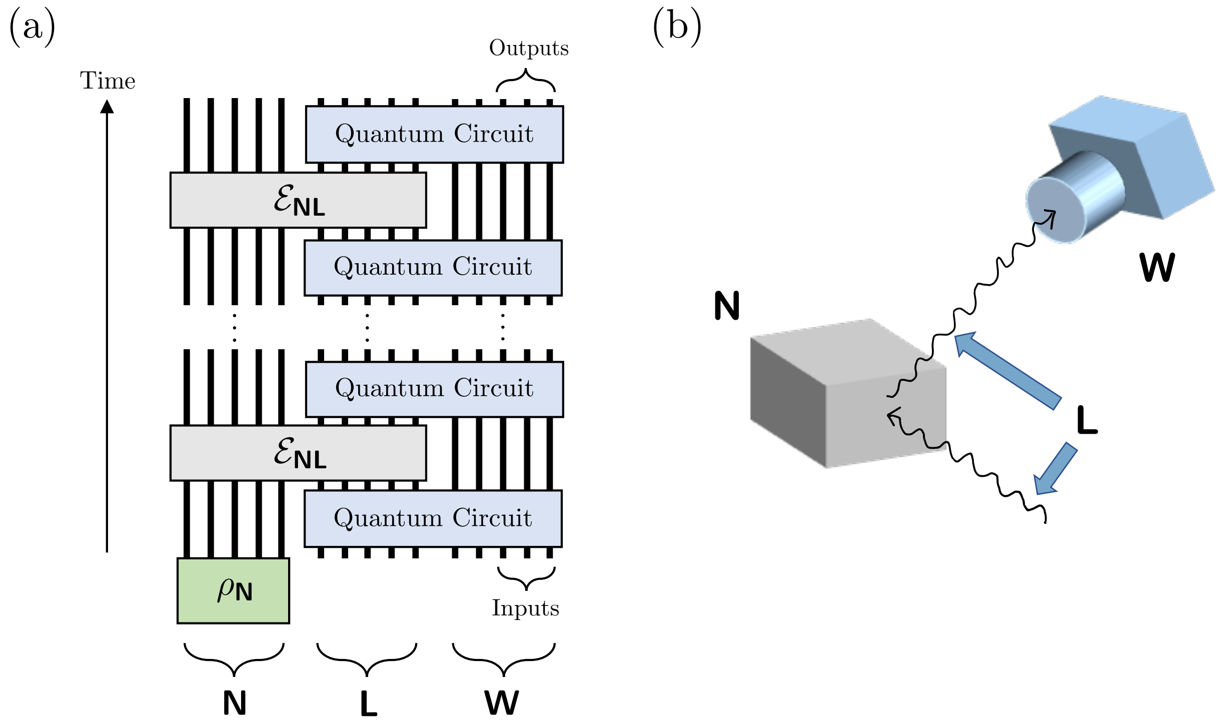

Supplementary Figure 1. *Circuit structure of a QUALM.* (a) Schematic illustrating the structure of a QUALM as an interaction between Nature and the experimentalist's controlled degrees of freedom. Here $\mathbf{N}$ represents the 'Nature' register, $\mathbf{L}$ is the 'lab' register, and $\mathbf{W}$ is the 'working space' register. The experimentalist does not have direct measurement access to the $\mathbf{N}$ register which should be thought of as the "hidden" degrees of freedom of the physical system on which the experiment is conducted. The initial state on $\mathbf{N}$ is $\rho_{\mathbf{N}}$, and the input and output subsets of $\mathbf{W}$ are specified. Each thick wire corresponds to a 'doubled wire' representing both a Hilbert space and its dual, since the input is a density matrix. For further explanation see A. (b) Illustration of a QUALM for X-ray diffraction, where $\mathbf{N}$ is the crystal sample, $\mathbf{L}$ consists of the X-ray photons (including the incoming and outgoing ones), and $\mathbf{W}$ contains the camera and other lab equipment for taking and processing the image.

gone. By contrast, one can consider a more advanced experimental setup in which photons can be prepared in EPR pairs, and after one member of a pair interacts with the sample in **N**, further quantum gates can be applied jointly to the pair. Perhaps the photons can be sent back to interact with the sample again, without losing coherence. One would expect that the second setup has an advantage over the first one, at least for some tasks. Most traditional experiments in condensed matter and atomic physics are like the first case, where only classical information is extracted from the physical system being studied, and measurements may destroy the quantum state. Modern experimental techniques have enabled more and more examples of the second kind.

Motivated by these physics considerations, in this work we compare the following two types of QUALMs, describing experimental setups with different access capabilities. The first type allows the application of arbitrary quantum circuits to **L** and **W**, which will therefore be able to create and manipulate quantum entanglement between them. We refer to such QUALMs as *coherent access* QUALMs. The second type only allows local operations and classical communication (LOCC) between the lab system **L** and the working space **W**. In addition, each time after applying the lab oracle, the state of **L** is measured such that no *coherent* quantum information can survive and be acted upon by the lab oracle again. We refer to these QUALMs as *incoherent access* QUALMs.

It seems to be very natural to ask: how advantageous is the second type of experiments (coherent QUALMs) over the first (incoherent) type? What do we gain by allowing coherent access to the physical system we are trying to study?

Our main technical contribution is to provide two physically motivated problems, for which coherent access QUALMs can be exponentially more efficient than their incoherent counterparts, even if the incoherent access QUALM is allowed to employ *adaptive* strategies.

We start by considering the following problem. We informally formulate the problem below, to be made precise later:

Definition 3 (*The fixed unitary problem).* (Roughly) Consider two lab oracles LOQ_ℓ and LOP_ℓ, corresponding to two physical systems. The first lab oracle LOQ_ℓ picks a random unitary, remembers it (forever), and then subsequently applies that *same* unitary to **L** each time the oracle is called. (ℓ labels the number of qubits in **L**.) By contrast, the second lab oracle LOP_ℓ applies a new random unitary to **L** each time the oracle is called. The goal is to distinguish between LOQ_ℓ and LOP_ℓ with success probability at least $0.5+\varepsilon$ for some constant $\varepsilon > 0$.

We formally describe the aforementioned lab oracles in Supplementary Note 3. Physically, this problem is a toy model for distinguishing a Floquet system from a random quantum circuit. The time evolution operator in the former has a discrete time translation symmetry, while the time evolution operator of the latter is stochastic, with randomness that is uncorrelated in time. We have that LOQ_ℓ implements evolution by a Floquet system with period T, which is only accessed every time T. On the other hand, LOP_ℓ implements evolution by a random quantum circuit where each time evolution step is enacted by an independent Haar random unitary. Though the respective superoperators of LOQ_ℓ and LOP_ℓ only seem to apply unitaries on **L**, the Nature system **N** is needed in order to 'remember' the unitary applied, or pick a new (random) unitary each time.

There is a very simple coherent access QUALM that distinguishes between LOQ_ℓ and LOP_ℓ. We first prepare an entangled state between **L** and **W**, namely the maximally entangled state[1]

$$|\Psi\rangle = \frac{1}{\sqrt{2^\ell}} \sum_{x\in\{0,1\}^\ell} |x\rangle_{\mathbf{L}} \otimes |x\rangle_{\mathbf{W}}\,. \tag{1}$$

We only need to utilize ℓ of the qubits in the **W** subsystem here. We first call the lab oracle to act on the **L** part of $|\Psi\rangle$, giving us $(U_1\otimes\mathbb{1})|\Psi\rangle$. Then we swap wires so that the **L** part of the state is now in **W**, and the **W** part of the state is now in **L**. We subsequently call the lab oracle again, and it acts on the **L** subsystem, giving us

$$\frac{1}{\sqrt{2^\ell}} \sum_{x\in\{0,1\}^\ell} U_2|x\rangle_{\mathbf{L}} \otimes U_1|x\rangle_{\mathbf{W}}\,. \tag{2}$$

Then we implement a SWAP test to essentially calculate $|\mathrm{tr}(U_1^\dagger U_2)|^2$. If $U_1 = U_2$, then the lab oracle must have been

[1] In fact, the QUALM we present for the task (in the proof of Theorem 3), does not require the preparation of a maximally entangled state but starts simply with the all 0 state (and hence is even simpler to realize in the lab); however one of our follow-up results, Theorem 5, does require it.

LOQ_ℓ. On the other hand, if $|\mathrm{tr}(U_1^\dagger U_2)|^2$ is near zero, then $U_1 \neq U_2$ and the lab oracle was LOP_ℓ. Thus, the QUALM complexity in the entangled access model is $\mathscr{O}(\ell)$ – see Theorem 3.

Our first main technical result is the following:

Theorem 1 (*Exponential lower bound for incoherent adaptive QUALMs for the fixed unitary problem).* (Roughly) For any incoherent access QUALM for the "fixed unitary problem" on ℓ qubits (i.e., L has ℓ qubits), its QUALM complexity is lower bounded by an exponential in ℓ. This holds even if the QUALM is allowed to employ adaptive strategies.

We note that the upper bound on the QUALM complexity in the coherent access case is very simple; it is the lower bound in the incoherent access case that requires the technical effort. Moreover, we find a fairly straightforward proof of an exponential lower bound for the incoherent access case if we do not insist on allowing the protocol to be adaptive. Once adaptive protocols are considered, the argument becomes far more complicated. We use Weingarten functions and a substantial dose of combinatorics to arrive at our desired result. Physically, our result is consistent with the intuition that chaotic Hamiltonian evolution or Floquet dynamics (for which LOQ_ℓ is a toy example) can emulate stochastic dynamics (for which LOP_ℓ is a toy example), even if the former is deterministic and time-translation invariant. Details of the setup of this problem, and the proof, are presented in Supplementary Note 3.

The above bounds constitute the first example of a coherent measurement protocol for a physically motivated experiment, which is provably exponentially more efficient than any incoherent experiment. Furthermore, this turns out to be true even if the coherent access QUALM is extremely simple, such as the SWAP test; and even if the incoherent QUALM is allowed to be adaptive.

Two immediate corollaries of the above Theorem 1 are as follows. One can consider the *fixed state problem*, in which one is asked to distinguish between the following two lab oracles: the first picks a Haar random ℓ qubit state and generates that same state on the L subsystem each time the oracle is called, while the other oracle generates on L a newly picked Haar random state each time the oracle is called. We have:

Corollary 1 (*Exponential lower bound for the fixed state problem).* The QUALM complexity of the fixed state problem is exponential in ℓ in the unentangled adaptive access setting. (The QUALM complexity of this problem in the entangled access case is at most linear in ℓ.)

A related problem, which we call the *non-unitarity problem*, is along the following lines. One is asked to distinguish between two lab oracles: the first applies a (fixed) Haar random ℓ qubit unitary to the L system, while the other applies a completely depolarizing channel to L. We have the corollary:

Corollary 2 (*Exponential lower bound for the non-unitarity problem).* The QUALM complexity of the non-unitarity problem is exponential in ℓ in the incoherent adaptive access setting. (The QUALM complexity of this problem in the coherent access is at most linear in ℓ.)

We next turn to another physically-motivated task:

Definition 4 (The symmetry distinction problem). Let our quantum lab oracle be one of the following: either, it applies a Haar random **unitary** to the L system every time it is called, it applies a Haar random **orthogonal** matrix to L every time it is called, or it applies a Haar random **symplectic** matrix to L every time it is called. The problem is to distinguish which of the three oracles we have, with a non-negligible probability of success.

If one is allowed coherent access, then one can use the SWAP test, with a little more sophistication, to determine the oracle; this requires at most a linear number of gates. Extending the techniques used in the proof of Theorem 1, we prove that for the symmetry distinction problem the incoherent access QUALM complexity is at least exponential, even if adaptive strategies are allowed.

Theorem 2 (*Exponential lower bound for incoherent adaptive QUALMs for the symmetry distinction problem).* (Roughly) For any incoherent access QUALM for the symmetry distinction problem on ℓ qubits, its QUALM complexity is lower bounded by an exponential in ℓ. This holds even if the QUALM is allowed to be adaptive.

The above result is of potential experimental interest. Indeed, symmetries are essential features of quantum many-body systems, and their measurement is a central desiderata of experimental physics. Our result establishes, in a prototype setting, that in some cases such symmetries may be discovered with the aid of entanglement using exponentially fewer resources than conventional methods allow in contemporary experiments.

Remark 1 (*Unitary designs*). We note that in all the above results, we consider Haar random unitaries and states for ℓ qubits. These are in general non-physical for large ℓ. To bring our results somewhat closer to physics, we note that our results also apply if we consider, instead of Haar random unitaries, random circuits of sufficiently large polynomial depth. The idea is that such random quantum circuits on ℓ qubits, of depth t, become what is known as approximate unitary k-designs for t polynomial in ℓ, k[54]. Such distributions over unitaries can replace the Haar measure to approximate, with arbitrarily small inverse exponential error, integrals of the form

$$\int_{\text{Haar}} dU U^{\otimes k} A (U^{\dagger})^{\otimes k} \tag{3}$$

which is precisely the form of the equations on which our proofs rely (see, e.g., Supplementary Equation (44)). If our unitaries are drawn from a unitary k-designs, for k the number of lab oracle calls (which we assume to be polynomial), then all our results follow, since all calculations only incur an exponentially small error. This brings our results one step closer to physical reality; see Subsection 1.6 for more on this point.

Supplementary Note 1.4: Comparison with related results

The advantage of coherent access over incoherent access is an example of the hierarchy of capabilities of QUALMs. Other classes of access models can also be defined. For example, the most restricted type is classical access, for which we are only allowed to apply the lab oracle superoperator to classical states. Further, we are only allowed to measure the outcome of such applications of the superoperator in a classical basis. QUALMs can be classified by different access types, ranging from the most restricted (classical access) to the least restricted (coherent access). Such a classification is also helpful for putting our contributions in the context of other results, as is shown in Supplementary Table 1. Between the classical case (the column labeled by **Comp. basis** in the table) and the coherent access case, we can consider the following access types:

- **Local-local**. **L** consists of ℓ qubits, and the only states we are allowed to prepare for the lab oracle to act on are product states. Once a lab oracle acts on a product state, we are only allowed to measure the result in a product basis. Note that pre- and post-processing can entail full quantum computation.
- **Single register**. The QUALM allows generic gates to be applied to **L** so that the qubits in **L** can become mutually entangled, but **L** and **W** only interact via LOCC's and so they cannot become entangled with one another. In addition, the applied LOCC's may not depend on previous measurement results; in other words, the QUALM is non-adaptive. In Supplementary Table 1 we consider examples of single register access in which the **N** register is empty. In this case, one can think of the single register model as if the lab oracles queries are all applied in parallel, and the resulting information is processed thereafter.
- **Incoherent access**. We have discussed this type in the previous subsection. Compared with the single register setting, the main difference is that adaptive strategy is allowed. There is still no quantum coherence between different applications of the lab oracle, but the LOCC applied can depend on all previous measurement results.

Simon's algorithm[91] is an example of *local-local* access QUALM, which achieves an exponential advantage over any classical algorithm with access to the same oracle. This also clarifies that the exponential advantage we prove is fundamentally different from that discussed in Simon's algorithm.

In the *coherent* column we placed the important protocol which solves the Hidden subgroup problem[35]; this protocol uses coherent access to the registers, and requires only polynomially many queries. However, the exponential advantage

**Oracle/ access**	**Binary**	**Local-local**	**Single register**	**Incoherent access**	**Coherent Access**
**Classical**		Simon's algorithm*[91]	Dihedral, Affine and Heisenberg HSP[84]<br>Conjectured exponential query advantage over binary	?	HSP for general groups[35]<br>Conjectured exponential query advantage over binary<br><br>Exponential advantage over (*adaptive*) incoherent access from generalized Simon's task[19] and Welded Tree problem[30]
**Quantum**		?	?	?	State tomography: quadratic query advantage over single register[50,77]<br><br>Exponential query advantage in distinguishing coset states for the dihedral group over (*non-adapative*) single register[11]<br><br>Exponential query advantage for tomographic protocols[57]<br><br>**This work***: Exponential advantage over (*adaptive*) incoherent access for experimental tasks

Supplementary Table 1. Comparison to known results with different access types to the lab oracles. The meaning of the rows and columns is explained in the text.

in terms of query complexity is only conjectured, since no known lower bounds on more restricted access types are known. Moreover, and very importantly, the quantum protocols suggested are not known to be efficient in terms of gate complexity and thus also not in terms of QUALM complexity; realizing them by efficient circuits would imply an efficient algorithm for these problems, resolving a quarter-century-old open problem. For context, we also have a *single register* column where we placed the result of[84] which provides a protocol that does not require coherence between the registers, but works only for a restricted set of groups. This protocol is likewise not known to be gate efficient and it has not been proven to have an exponential query complexity advantage over classical algorithms.

In the quantum row there is the quadratic sampling advantage of state tomography protocols when using entanglement[50,77]; we note that this improvement is merely quadratic in query complexity. Importantly, the advantage is not known to be achievable using a gate-efficient protocol (also, the lower bound proof does not hold for adaptive single register access; in fact, it seems that adaptive protocols do perform better than non-adaptive ones in this case[50]).

Two examples related to our contribution are[11] and[57]. The former provides a proven exponential advantage of the coherent versus the single register access, for a quantum state distinction problem emerging from the dihedral HSP. However, very importantly, the entangled protocol suggested has *exponential* gate complexity, and so the exponential advantage is restricted to query complexity and is not known to hold in QUALM complexity. In addition, the advantage is again known to hold only in the restricted case in which the single register access is non-adaptive. The work of[57] presents a tomography task in which is there an exponential query advantage of coherent versus (adaptive) incoherent access. However, the coherent protocol of[57] has exponential gate complexity, and thus its advantage is restricted to query complexity.

It is natural to ask whether it is possible to massage the well-known exponential advantages[12,18,40,45,85,86] of quantum communication complexity[93,94] into an exponential separation between coherent and incoherent access QUALMs, as the LOCC restriction on incoherent access QUALMs seems to capture exactly the difference between quantum and classical communication. Indeed, communication complexity can be embedded in the QUALM framework (see Subsection 2.5). However, communication complexity only counts the number of qubits sent from one party to another; by contrast, the full QUALM complexity additionally takes into account the complexity of pre- and post-processing those qubits, and performing computation on them (as needed to achieve the communication task). More concretely, exponential advantages in the communication complexity setting occur when a classical communication task that requires k communicated bits only requires $\mathscr{O}(\log(k))$ bits (or qubits) in the quantum setting. In the QUALM framework, such a savings will have little effect on the QUALM complexity, since the gate complexity is already at least the size of the input k. Thus, to the best of our knowledge, the exponential quantum advantages in the communication complexity model do not provide examples of exponential separations in QUALM complexity between coherent and incoherent access.

Interestingly, two recent works[19] and[30] from a seemingly unrelated area of quantum computation, and a different motivation, do provide exponential separations between coherent and incoherent, adaptive access in terms of both gate and query complexity. These results are presented in a very different language, as their original goal was to prove a separation in *depth* hierarchies. Some work is required to reformulate the results within the QUALM framework, and derive their implication of such an exponential separation between the two access types. The problems at hand are respectively a recursive generalization of Simon's problem, and the welded tree problem; these problems are somewhat contrived, and are not natural from the point of view of applications in experimental physics.

Our work is the first to demonstrate an exponential advantage in QUALM complexity between coherent and incoherent access QUALMs for experimentally motivated tasks; this holds even when the incoherent QUALM is allowed to be adaptive. Moreover, this exponential advantage is achieved using an extremely simple coherent access QUALM, based solely on the SWAP test, and thus gives rise to the hope that it, or a variant thereof, can be implemented experimentally.

Supplementary Note 1.5: Related models

QUALMs are a hybrid between quantum circuits with access to classical (or quantum) oracles[76], and quantum interactive protocols[64]. The additional Nature register **N** enables modeling certain essential characteristics of physical experiments: in particular, the indirect access to the "input" physical system to be measured, their hidden "memory" between different applications, and the nature of the interaction between the experimental apparatus and the physical system. Yet, formally, QUALMs are tightly related to quantum oracle algorithms, as well as to quantum interactive protocols (and variants thereoff such as quantum strategies[49] and quantum combs[21]), and it is very likely that results from these areas will

be useful for the design and analysis of QUALMs. It is likely that methods and techniques from the area of quantum communication complexity will also be useful, due to the relations mentioned in Subsection 1.4.

The notion of QUALMs we introduce here is related to another notion which has been recently studied fairly intensively. In our framework, we are interested in modeling a *physical experiment* resulting in a *classical* outcome of a measurement. This can be viewed as a function, computed by the QUALM, whose input is the lab oracle and a bit string of classical data from the lab, and whose output is a bit string (see Definition 7). In fact, as we describe in Subsection 2.2, both input and output registers can be generalized to be quantum. Motivated by quantum algorithmic questions, recently researchers took interest in problems which can be cast in a tightly related model: a quantum circuit which makes use of a quantum oracle by black box access. The circuit gets as an input a *quantum state*, and outputs another *quantum state*. Such a process generates a *quantum channel* which depends on (or is a function of) the quantum oracle being used as a black box (the work of[41] formally defined this notion as a Task). This is similar to the notion of task we use in our QUALM framework, except without the interactive ingredient; thus, the Nature register $\mathbf{N}$ is missing. Several works investigated in this model how oracle access to a unitary U can be used to implement various channels which are functions of U; for instance, applying controlled-U[6,33,41,92], U^*[75], U^T and U^{-1}[82,83], raising U to some fractional power[46,90], and more. Ideas from this direction could be relevant for QUALM purposes.

We mention that QUALMs were in part inspired by the work[27], which developed quantum information-theoretic measures of spacetime correlations. (See also related work on quantum combs[21] and quantum process tensors[80].) For a given physical system, if we are only allowed to probe a spatial region A at a given time, the results of all possible measurements are determined by the reduced density operator on A at that time. If we are instead allowed to access the physical system at several different spacetime regions, which may be timelike or spacelike separated, the correlation functions cannot be characterized by an ordinary density operator. Instead,[27] considered ancilla systems which couple to the physical system at the specified spacetime regions. By an appropriate choice of the ancillas and their couplings to the physical system, all correlation functions for the specified spacetime regions can be determined by the density operator of the *ancilla system*, which is called the "superdensity operator" of the original system for the specified spacetime regions. From the QUALM point of view, the superdensity operator can be viewed as the output of a particular type of QUALM. The physical system being studied corresponds to $\mathbf{N}\otimes\mathbf{L}$, with its dynamics specified by the lab oracle superoperator. The lab system $\mathbf{L}$ contains all spacetime regions that are accessible to the experimentalist. The ancillas correspond to $\mathbf{W}$, which is coupled to $\mathbf{L}$. Each coupling between $\mathbf{L}$ and $\mathbf{W}$ utilizes a new (i.e., previously unused) subsystem of $\mathbf{W}$ and otherwise there are no gates coupling different subsystems of $\mathbf{W}$. The superdensity operator of the $\mathbf{N}\otimes\mathbf{L}$ system is simply the final density operator of $\mathbf{W}$. The superdensity operator formalism can be usefully applied to analyze a variety of different tasks. For example, Ref.[26] studied the causal influence between different space-time regions with superdensity operators, which can be viewed as a task achieved by this particular type of QUALM.

Supplementary Note 1.6: Comments and open questions

Our motivation for this study is physics. We believe that QUALMs lay the groundwork for a theory of general quantum experiments and measurements, and provide a framework to study their complexity. Further, an understanding of QUALMs enables the design of novel and more efficient experiments; this is of particular importance in the present era of quantum computation in which many new experimental tools are about to become available. Conceptually, we hope the tools we introduce in this paper aid in the understanding of various types of QUALMs, and in the development of more sophisticated QUALMs to enable better precision, more savings in resources, and new insights into the design of physical quantum experiments.

In this work, we provide evidence that entanglement and coherence can be truly *exponentially* advantageous when performing *experiments in the lab*. We note that this exponential advantage is fundamentally different than the usual exponential *algorithmic* advantage commonly associated with quantum speedups. We believe this provides a second, very important motivation for the physical realization of quantum algorithmic components.

Our results may have experimental applications; in particular, the problem of distinguishing a time-dependent random Hamiltonian from a fixed Hamiltonian, detecting non-unitarity, as well as the problem of detecting the symmetry of the physical system, are physically motivated questions; it would be very interesting to see the coherent experimental setups suggested here demonstrated experimentally.

A very important question that this work raises is the following. The exponential advantage of coherent access

demonstrated in this work is achieved in a toy setting which is not easy to realize: the U's considered are highly non-local (even if we consider the unitary design version as discussed at the end of Subsection 1.3), and the operations are assumed to be noise-free. Can our results be generalized to the noisy setting, and thus be brought closer to physical reality?

In particular, it is not hard to see that our protocols, which rely on the SWAP test, lose their quantum advantage in the presence of local independent noise with any constant probability $p > 0$ per qubit. One might ask if an example providing exponential advantage for coherent access QUALMs can be designed even for noisy systems, using quantum fault tolerance[2,63,65]. Indeed, if our entire protocol as well as the Haar random unitaries in the lab oracles(!) are encoded fault tolerantly, and the error rate is below the fault tolerance threshold, then one arrives at an example in which an exponential advantage of coherent access QUALMs persists even in the presence of noise. However, this leads to an extremely non-physical choice of the lab oracles; moreover, the QUALMs themselves cannot be assumed to be fault tolerant in the NISQ era[81]. It is an important open question to clarify whether similar advantages can be achieved in the NISQ era.

Another natural attempt to make the results more realizable is to study QUALMs with access to lab oracles of much smaller computational complexity. We note that the natural first attempt, namely characterizing k-local time-independent Hamiltonians for $k = \mathscr{O}(1)$ to some given accuracy ε, is already fairly well-understood: given access to such a Hamiltonian as a lab oracle, the description of the Hamiltonian can be measured to within arbitrary accuracy very efficiently by local-local QUALMs (by applying Pauli measurements) using polynomially many samples and local measurements (see, e.g.[71]). In this case, there does not seem to be a significant advantage to using QUALMs of more coherent access types. A natural next step concerns the question of lab oracles which apply shallow (i.e. constant depth) quantum circuits. It is unclear whether this is possible to achieve efficiently by a QUALM (see the impossibility results of[9], as well as the sample-efficient entangled *and* adaptive protocols for a related task[23]).

A third approach involves overcoming a technical problem. The replacement of the Haar random unitaries in our examples by unitary designs, as suggested in the remark in Subsection 1.3, indeed makes the setting more realistic. However, it has a drawback: once we fix the distribution over unitaries to be a unitary k-design for a particular k, we can always increase the number of queries k' to be larger than k (but still polynomial), in which case the approximation of the Haar measure breaks down and our impossibility proofs falls apart. Thus, unitary designs do not offer an example of an exponential separation between coherent and incoherent access QUALMs.

One could hope to instead use *pseudo-random unitaries*, defined in[59]. Pseudo-random unitaries are both practical to construct, and under certain computational assumptions are indistinguishable from Haar random unitaries by any polynomial circuit. Thus, pseudo-random unitaries could replace Haar random unitaries in the fixed unitary problem and the symmetry distinction problem while still maintaining an exponential separation between coherent and incoherent access QUALMs. We note that any proofs in this setting would rely on the computational assumptions inherent in pseudo-randomness, and further would have to additionally impose the restriction that the gate complexity of the QUALM is polynomial (in our proofs, we only lower bound the query complexity). In any case, the constructions of efficient pseudo-random unitaries, suggested in[59], are not yet proven to be secure. Finding a realistic and physically-motivated setting for which an exponential advantage between coherent and incoherent access QUALMs holds thus remains an important open problem.

Another important problem is of course to come up with further advantages of different QUALMs, for problems of physical interest. We could for example ask a more sophisticated question like[2]: could there exist an efficient QUALM which detects the topological phase of a system? For instance, is it possible to define an ensemble of "random" topological phases which are hard to distinguish by adaptive, sequential single register measurements, but which can be distinguished efficiently using a QUALM (presumably, once which is allowed coherent access)?

It would be very interesting to come up with coherent access QUALMs which achieve exponential (or even polynomial) advantage, by leveraging coherence in more sophisticated ways than just the SWAP test and its variants. What other types of quantum computation tools are available in this context? Likely, inspiration from quantum algorithms will be helpful.

It is interesting to clarify the relations between the other types of access for QUALMs (i.e., comparing between other columns of Supplementary Table 1). Is there a provable exponential (or even polynomial) advantage of single register access over local-local access? Of particular interest is whether there is an exponential advantage of incoherent access

[2] We thank Subir Sachdev for suggesting this.

over single register access; this captures the question of the power of adaptiveness in incoherent experiments; In other words we ask: does adaptiveness help in the incoherent setting (see[43,52])? Relatedly, a problem which we leave open is: what is the power of the incoherent access model when we remove the requirement of complete measurements in between lab queries? Is the resulting, modified model still exponentially weaker than the coherent access model?

We hope that this work deepens our understanding of how quantum computing methods can enable more sophisticated and precise quantum experiments. There are many problems left open; much more work is needed in order to improve our understanding of the limitations and possibilities of quantum algorithmic measurements.

Supplementary Note 1.7: Organization

The rest of the supplementary information is organized as follows. In Supplementary Note 2 we provide the definitions of the main players: tasks, lab oracles, QUALMs, and QUALM complexity. We also provide a few examples of how various familiar tasks can be cast in the QUALM framework. In Supplementary Note 3 we introduce the fixed unitary problem and provide both a coherent QUALM for it (Theorem 3) as well as a proof that the QUALM complexity for any incoherent QUALM that solves it is exponential (Theorem 4); Supplementary Note 4 provides several corollaries and extensions of the previous section. In Supplementary Note 5 we introduce the symmetry distinction problem, provide the coherent QUALM for it (Theorem 5) and prove an exponential lower bound for incoherent QUALMs for the task (Theorem 6). The Appendices contain some basic introduction to the diagrams we use throughout, as well as the necessary background on Haar integrals.

Supplementary Note 2: QUALMs and Lab Oracles: Definitions

Supplementary Note 2.1: Notation

Throughout the paper, we denote subsystems (which will all consist of finitely many qubits) by $\mathbf{N},\mathbf{L},\mathbf{W},\ldots$, and we use the same notation to denote their associated Hilbert spaces. The number of qubits in each subsystem will be denoted by lowercase letters $n,\ell,w,\ldots$ and the set of states (density matrices) on each subsystem will be denoted by $\mathscr{D}(\mathbf{N}),\mathscr{D}(\mathbf{L}),\mathscr{D}(\mathbf{W}),\ldots$; we similarly denote classical probability distributions on $\{0,1\}^k$ by $\mathscr{D}(\{0,1\}^k)$. We will often refer to the union of two or three non-intersecting registers; in this case we will denote the resulting Hilbert space (as well as the set of qubits) by concatenating the letters corresponding to the registers, e.g. $\mathbf{LW}$ (although sometimees we will write $\mathbf{L}\otimes\mathbf{W}$).

Operators, superoperators, as well as sets thereof, will be denoted using a calligraphic font: $\mathscr{E},\mathscr{Q},\mathscr{O}$ etc. We will often abuse notation, and refer to an ordered sequence of superoperators $\mathscr{Q}=(\mathscr{Q}_1,\mathscr{Q}_2,\ldots,\mathscr{Q}_k)$ as equal to the operator which is the result of applying the superoperators in the sequence in the given order; that is, we will refer to $\mathscr{Q}$ as the superoperator $\mathscr{Q}=\mathscr{Q}_k\circ\cdots\circ\mathscr{Q}_2\circ\mathscr{Q}_1$.

As we will see below, lab oracles, which are not simply operators but a pair of a superoperator and a state, will be denoted by $\mathsf{LO}=(\mathscr{E}_{\mathbf{NL}},\rho_{\mathbf{N}})$.

Supplementary Note 2.2: Quantum Algorithmic Measurements

Throughout this subsection, we will consider three Hilbert spaces:

- The "Nature" Hilbert space $\mathbf{N}$ of n qubits, to which the experimentalist has no direct access;
- The "lab" Hilbert space $\mathbf{L}$ of ℓ qubits, which consists of the degrees of freedom of the physical system which the experimentalist *can* access,
- A third Hilbert space $\mathbf{W}$ of w qubits can be thought of as the "working space" of the experimentalist – it corresponds to the additional degrees of freedom which the experimentalist uses during the experiment.

We imagine these three Hilbert spaces as being subsystems of a total Hilbert space $\mathbf{H}$; that is, $\mathbf{H}=\mathbf{N}\otimes\mathbf{L}\otimes\mathbf{W}$.

Our first definition is that of the *lab oracle*; it is an attempt to capture the allowed or available interactions between the experimentalist and the *physical system* on which the experiment is performed. The setup is summarized in Supplementary Figure 2.

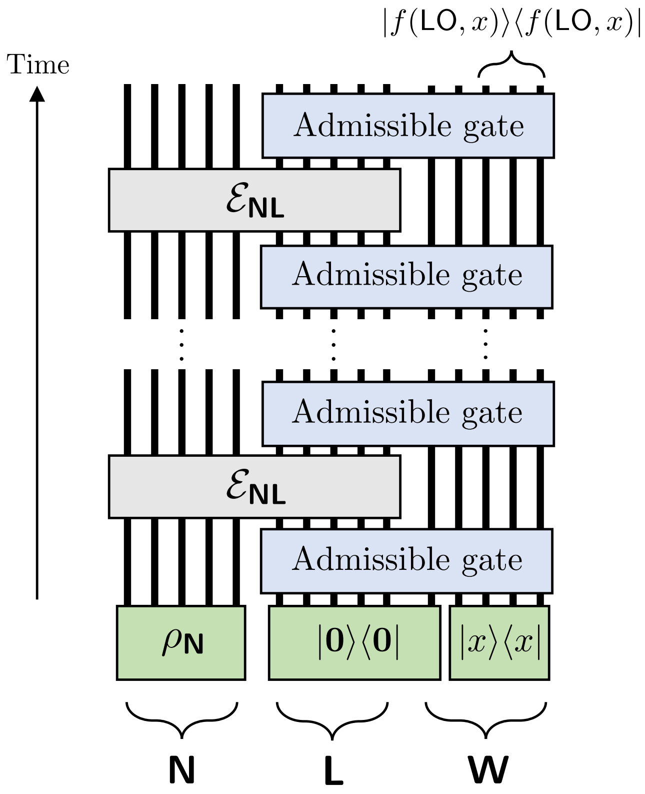

Supplementary Figure 2. *QUALM applied to a lab oracle.* We use the diagrammatic notation explained in A. The green boxes represent the initial state $\rho_{\mathbf{N}} \otimes |\mathbf{0}\rangle\langle\mathbf{0}| \otimes |x\rangle\langle x|$ where x is some bit string. The grey rectangles are the lab oracle superoperator $\mathscr{E}_{\mathbf{NL}}$ as per Definition 5. A QUALM (Definition 8) consists of a sequence of applications of either admissible gates $\mathscr{G}_i \in \mathscr{G}$ (see Definition 6) or the lab oracle superoperator $\mathscr{E}_{\mathbf{NL}}$. The output of the QUALM is on a subsystem $\mathbf{S}_{\text{out}}$, indicated by the bracket at the top right of the diagram. The QUALM (blue part of the diagram) and lab oracle superoperators (gray parts of the diagram) jointly comprise a quantum circuit mapping quantum and classical inputs (green parts of the diagram) to a final state on $\mathbf{S}_{\text{out}}$. If this map produces a state $|f(\mathsf{LO},x)\rangle$ which encodes a specified function f on lab oracles and initial bit strings that is targeted in a 'task' (see Definition 7), we say the QUALM achieves that task.

Definition 5 (*Lab Oracle).* A lab oracle is specified by a pair $\mathsf{LO} = (\mathscr{E}_{\mathbf{NL}}, \rho_{\mathbf{N}})$ where $\mathscr{E}_{\mathbf{NL}}$ is a quantum superoperator (i.e. a completely positive trace-preserving map) on $\mathbf{N} \otimes \mathbf{L}$ and $\rho_{\mathbf{N}}$ is state on $\mathbf{N}$. The set of lab oracles is denoted by $\mathscr{LO}(\mathbf{N}, \mathbf{L})$.

In some circumstances it is suitable to generalize this definition so that the lab oracle state $\rho_{\mathbf{N}}$ is instead an initial state jointly on $\mathbf{N}$ and (a subset of) $\mathbf{L}$.

We regard the experimentalist as having command over $\mathbf{L} \otimes \mathbf{W}$. The idea is that the nature subsystem $\mathbf{N}$ is initialized in the state $\rho_{\mathbf{N}}$, and that the only way to interrogate it is via $\mathscr{E}_{\mathbf{NL}}$ which couples $\mathbf{N}$ to $\mathbf{L}$. The experimentalist can then process the information accessed by this coupling by performing quantum computation on $\mathbf{L} \otimes \mathbf{W}$.

In the most general setting, we allow the experimentalist to perform arbitrary quantum operations on $\mathbf{L}$ and $\mathbf{W}$, i.e. the experimentalist has access to a universal quantum gate set. In other circumstances, it is useful to restrict the allowed operations to be classical, so as to compare with the fully quantum case. There are other useful restrictions, depending on the context. As such, it is useful to define the "admissible gates" allowed for the experimentalist to use on $\mathbf{L} \otimes \mathbf{W}$. (As usual, we allow the gates to more generally be superoperators.) We have the following definition:

Definition 6 (*Admissible gates*). We denote by $\mathscr{G}$ a set of "admissible gates", namely a set of quantum superoperators acting on $\mathbf{L} \otimes \mathbf{W}$. (Note that superoperators include measurements.)

Now we define the notion of a *task* which corresponds to the "experimental problem" to be solved: it describes what it is that the experimentalist wants to measure. The experimentalist must achieve the task only by using the lab oracle superoperator, together with the operations at her disposal in her laboratory (i.e., the admissible gates on $\mathbf{L} \otimes \mathbf{W}$).

Definition 7 (*Task*). A 'task' is a tuple $\mathsf{Task} = (\mathbf{S}_{\text{in}}, \mathbf{S}_{\text{out}}, f, \mathscr{G})$, associated with a given system $\mathbf{N} \otimes \mathbf{L} \otimes \mathbf{W}$ (which is usually implicit). Here, $\mathbf{S}_{\text{in}}$ is a p-qubit subsystem of $\mathbf{W}$, $\mathbf{S}_{\text{out}}$ is a q-qubit subsystem of $\mathbf{W}$, f is a function

$$f : \{\mathsf{LO}_0, \mathsf{LO}_1, \mathsf{LO}_2, \ldots\} \times \{0,1\}^p \longrightarrow \{0,1\}^q \,,$$

and $\mathscr{G}$ is a set of admissible gates on $\mathbf{L} \otimes \mathbf{W}$. In the domain of f, $\{\mathsf{LO}_0, \mathsf{LO}_1, \mathsf{LO}_2, \ldots\}$ is a set of lab oracles (here we denoted this set as discrete, but of course one can also consider a continuous set of lab oracles as input), i.e. a subset of $\mathscr{LO}(\mathbf{N}, \mathbf{L})$

This definition can be thought of as follows. Given a set of lab oracles which represent possible arrangements of 'Nature', the task is to compute the function f which takes as input a lab oracle, some classical lab settings (i.e., bit strings in $\{0,1\}^p$), and outputs a classical 'experimental result' (i.e., bit strings $\{0,1\}^q$). The task is to be achieved by constructing a circuit from admissible gates in $\mathscr{G}$, in conjunction with interspersed calls to the lab oracle superoperator.

Remark 2 (*A* Task *with a probabilistic output* (*Generalizing Definition* 7)). In many situations it is more natural to talk about output *probability distributions* and define f to be

$$f : \{\mathsf{LO}_0, \mathsf{LO}_1, \mathsf{LO}_2, \ldots\} \times \{0,1\}^p \longrightarrow \mathscr{D}(\{0,1\}^q) \,. \tag{4}$$

Such is the case for classical sampling tasks, as well as in the context of the task of distinguishing between lab oracles, which is the main focus of this paper; Example 2 in Subsection 2.5 demonstrates the usefulness of this generalization when considering continuous sets of lab oracles. In Definition 16 and the surrounding text, we discuss the fact that this generalized notion of a task (i.e., with f's of the form in Supplementary Equation (4)) reduces to Definition 7 for the distinguishing tasks which are the main focus of the paper (Definitions 16 and 23).

Remark 3 (*A* Task *with quantum input and output states*). We can also further generalize Definition 7 by upgrading the classical inputs and/or outputs of f to be density matrices. That is, f becomes

$$f : \{\mathsf{LO}_0, \mathsf{LO}_1, \mathsf{LO}_2, \ldots\} \times \mathscr{D}(\mathbf{S}_{\text{in}}) \longrightarrow \mathscr{D}(\mathbf{S}_{\text{out}}) \,. \tag{5}$$

This might be relevant when one is interested in sampling from Gibbs distributions of quantum Hamiltonians, or when considering QUALMs as *procedures*, or *subroutines* within other QUALMs.

Notice that if $\mathbf{N} \simeq \mathbb{C}$ (i.e. it is a trivial subsystem) and if the set of lab oracles is taken to be empty, then f in Definition 7 reduces to a map

$$f : \{0,1\}^p \longrightarrow \{0,1\}^q .$$

Accordingly, the 'task' is just that of computing the function f, using a quantum algorithm composed of gates from the gate set $\mathscr{G}$. See Subsection 2.5 for a more elaborate discussion of restricting QUALMs to standard quantum algorithms, as well as other examples.

We can now define the QUALM, which can be viewed as a specific choice of *protocol* for the execution of the desired experimental task.

Definition 8 ((QUALM)). A QUALM over the set of admissible gates $\mathscr{G}$ acting on registers $\mathbf{L}, \mathbf{W}$, is an ordered sequence of symbols $\mathscr{Q} = (\mathscr{Q}_1, \mathscr{Q}_2, ..., \mathscr{Q}_{\text{final}})$ from the alphabet $\mathscr{G} \cup \{\square\}$, together with a specification of input and output subsystems $\mathbf{S}_{\text{in}}, \mathbf{S}_{\text{out}} \subseteq \mathbf{W}$. Here, we are treating $\mathscr{G}$ as a set of symbols (i.e., each gate labels a distinct symbol) and likewise $\square$ is a symbol. Each QUALM has an associated map

$$\mathsf{QUALM} : \mathscr{LO}(\mathbf{N}, \mathbf{L}) \longrightarrow \mathsf{QuantumCircuits}(\mathbf{N} \otimes \mathbf{L} \otimes \mathbf{W}) . \tag{6}$$

This function takes in a lab oracle LO, and outputs a quantum circuit on $\mathbf{N} \otimes \mathbf{L} \otimes \mathbf{W}$. Specifically, $\mathsf{QUALM}(\mathsf{LO})$ 'compiles' a quantum circuit $\mathscr{Q} = (\mathscr{Q}_1, \mathscr{Q}_2, ..., \mathscr{Q}_{\text{final}})$ where each symbol in $\mathscr{G}$ is replaced by its corresponding gate, and each $\square$ is replaced by the superoperator $\mathscr{E}_{\mathbf{NL}}$ corresponding to LO. $\mathbf{S}_{\text{in}}, \mathbf{S}_{\text{out}}$ correspond to the input and output subsystems of the resulting circuit, respectively.

In less formal terms, a QUALM is a quantum circuit built out of an admissible gate set, where the circuit has designated spots for a lab oracle superoperator to be inserted, and specified input and output qubits.

Now we explain what it means for a QUALM to achieve a particular Task. We first define the output density matrix of a QUALM for a given lab oracle LO. The idea is to compile the quantum circuit $\mathsf{QUALM}(\mathsf{LO})$ for the lab oracle LO, and then to use it to evaluate $f(\mathsf{LO}, x)$. To do so, we construct the initial state of the circuit to be (i) $\rho_{\mathbf{N}}^{\mathsf{LO}}$ (i.e. the state corresponding to the lab oracle LO) on $\mathbf{N}$, (ii) $|x\rangle\langle x|$ on $\mathbf{S}_{\text{in}}$, and (iii) the zero state elsewhere. The full initial state will be denoted as $\rho_{\mathbf{N}}^{\mathsf{LO}} \otimes |x\rangle\langle x|_{\mathbf{S}_{\text{in}}} \otimes |\mathbf{0}\rangle\langle\mathbf{0}|$. We will run the initial state through the circuit $\mathsf{QUALM}(\mathsf{LO})$, and then trace out everything not in $\mathbf{S}_{\text{out}}$.

Definition 9 (Output density matrix of a QUALM on a given lab oracle and classical input). Given a QUALM on admissible gates $\mathscr{G}$, with specified input and output subsystems $\mathbf{S}_{\text{in}}$ and $\mathbf{S}_{\text{out}}$, its output density matrix on a given lab oracle LO and classical input x is defined to be

$$\rho_{\mathbf{S}_{\text{out}}}(\mathsf{QUALM}(\mathsf{LO}, x)) = \text{tr}_{\overline{\mathbf{S}}_{\text{out}}}\left\{\mathsf{QUALM}(\mathsf{LO})\Big[\rho_{\mathbf{N}}^{\mathsf{LO}} \otimes |x\rangle\langle x|_{\mathbf{S}_{\text{in}}} \otimes |\mathbf{0}\rangle\langle\mathbf{0}|\Big]\right\} . \tag{7}$$

To define the notion of a QUALM achieving a task, note that a Task specifies a function $f : \{\mathsf{LO}_0, \mathsf{LO}_1, \mathsf{LO}_2, ...\} \times \{0,1\}^p \longrightarrow \{0,1\}^q$.

Definition 10 (QUALM implementing a Task). A QUALM on admissible gates $\mathscr{G}$ with specified input and output subsystems $\mathbf{S}_{\text{in}}$ and $\mathbf{S}_{\text{out}}$ implements a $\mathsf{Task} = (\mathbf{S}_{\text{in}}, \mathbf{S}_{\text{out}}, f, \mathscr{G})$, if

$$\rho_{\mathbf{S}_{\text{out}}}(\mathsf{QUALM}(\mathsf{LO}_i, x)) = |f(\mathsf{LO}_i, x)\rangle\langle f(\mathsf{LO}_i, x)| \tag{8}$$

for all LO_i, x in the domain of f (i.e., for all $\mathsf{LO}_i \in \{\mathsf{LO}_0, \mathsf{LO}_1, \mathsf{LO}_2, ...\}$, and all $x \in \{0,1\}^p$).

We say that the QUALM implements the Task with error at most ε if for each input LO_i, x, we have

$$\Big\|\rho_{\mathbf{S}_{\text{out}}}(\mathsf{QUALM}(\mathsf{LO}_i, x)) - |f(\mathsf{LO}_i, x)\rangle\langle f(\mathsf{LO}_i, x)|\Big\|_1 \leq \varepsilon . \tag{9}$$

It is worthwhile to again compare to the setting of ordinary quantum algorithms, in which $f : \{0,1\}^p \to \{0,1\}^q$. Again, this is a restriction of f defined above in Definition 7 by letting the set of lab oracles in the domain be the empty set. If the QUALM superoperator is just a unitary circuit U, then Supplementary Equation (8) becomes

$$\text{tr}_{\overline{\mathbf{S}}_{\text{out}}}\{U\big(|x\rangle\langle x|_{\mathbf{S}_{\text{in}}} \otimes |\mathbf{0}\rangle\langle\mathbf{0}|\big)U^\dagger\} = |f(x)\rangle\langle f(x)| \tag{10}$$

which just says that if we input x on $\mathbf{S}_{\text{in}}$, then we output $f(x)$ on $\mathbf{S}_{\text{out}}$.

Remark 4 (*Subroutines, Error reduction by repetition, Approximation).* We remark that standard manipulations from the theory of algorithms carry over to the QUALM setting in a natural way. In particular, a QUALM can be used as a subroutine by another QUALM, as long as they both act on the same lab register $\mathbf{L}$ (like in standard subroutines, we can decide which qubit "wires" to glue from the original QUALM to the input qubits of the subroutine QUALM, and similarly for the outputs). The more natural definition for QUALMs in this case is the general one in which both the inputs and outputs are quantum registers, as in Supplementary Equation (5), unless we have restrictions on the input and output wires which make them classical.

Using such subroutine QUALMs, one can achieve error reduction (also known as amplification); this is a standard primitive in probabilistic algorithms[7], which is also needed in this work. This is done by a straightforward generalization of the way it is done for algorithms. For example, suppose we want to reduce the error probability of a given QUALM which achieves a certain deterministic task with error $1/3$; and further suppose that the image of the function f has a single output bit, measured at the end of the QUALM in the computational basis. One can construct a new QUALM', which first copies the p-bit input string x m times (for some desired amplification parameter m). It then applies QUALM as a subroutine m times, each time with a new set of qubits initialized to 0 (which together with the p qubits containing the appropriate copy of x, constituting the working register $\mathbf{W}$ of the particular subroutine). QUALM' then applies a majority calculation on all outputs of the m subroutines; this majority is the output bit of the QUALM'.

In the same manner, we can consider amplifications for QUALMs which compute probabilistic functions as in Supplementary Equation (5): if for two different lab oracles the output distributions are δ apart in total variation distance, one can amplify the distance by repetition and classical postprocessing.

Finally, for QUALMs which output probability distributions, one can talk about QUALMs which achieve certain tasks approximately. A metric on tasks is very naturally defined by considering the maximal total variation distance between the distributions which the two functions output for a given input, maximized over the lab oracles and classical inputs in the domain of the function.

We omit the formal details of these facts and definitions, as the details are completely straightforward as generalizations of probabilistic algorithms.

Supplementary Note 2.3: QUALM complexity

Having defined tasks and QUALMs, we now turn to defining QUALM complexity.

Definition 11 (*Gate complexity, query complexity, and QUALM complexity).* The gate complexity of a given QUALM over the admissible set of gates $\mathscr{G}$ is the length (i.e. the number of symbols from $\mathscr{G}\cup\{\square\}$) of the sequence $\mathscr{Q}$, minus the number of $\square$ symbols. We denote this by $\mathsf{GateComplexity}[\mathsf{QUALM}]$, and call this the QUALM gate complexity. Similarly, the query complexity is the number of $\square$'s appearing in $\mathscr{Q}$, and this is denoted by $\mathsf{QueryComplexity}[\mathsf{QUALM}]$. This is called the QUALM query complexity. We call the sum

$$\mathsf{GateComplexity}[\mathsf{QUALM}]+\mathsf{QueryComplexity}[\mathsf{QUALM}]=|\mathscr{Q}| \tag{11}$$

the QUALM complexity.

Definition 12 (*Exact QUALM complexity of a task).* Given a $\mathsf{Task}=(\mathbf{S}_{\text{in}},\mathbf{S}_{\text{out}},f,\mathscr{G})$, its exact QUALM gate complexity is

$$\min_{\{\mathsf{QUALM}:\,\mathsf{QUALM}\text{ implements }\mathsf{Task}\}}\mathsf{GateComplexity}[\mathsf{QUALM}]\,,$$

its exact QUALM query complexity is

$$\min_{\{\mathsf{QUALM}:\,\mathsf{QUALM}\text{ implements }\mathsf{Task}\}}\mathsf{QueryComplexity}[\mathsf{QUALM}]\,,$$

and its exact QUALM complexity is

$$\min_{\{\mathsf{QUALM}:\,\mathsf{QUALM}\text{ implements }\mathsf{Task}\}}\big(\mathsf{GateComplexity}[\mathsf{QUALM}]+\mathsf{QueryComplexity}[\mathsf{QUALM}]\big)\,.$$

Depending on the context, we can also specify that we take the minimum over all QUALMs which probabilistically implement the Task.

Note that it need not be the case that the exact QUALM gate complexity of a task plus the exact QUALM query complexity of a task be equal to the exact QUALM complexity of a task. This is because there may be a trade off between the gate and query complexities that arises when minimizing over their sum.

Remark 5 (*Modified definition of QUALM complexity).* In some situations, it may be useful to modify the definition of the QUALM complexity to be

$$\mathsf{GateComplexity}[\mathsf{QUALM}] + \lambda\, \mathsf{QueryComplexity}[\mathsf{QUALM}] \tag{12}$$

for a penalty factor λ. Accordingly, the exact QUALM complexity would be

$$\min_{\{\mathsf{QUALM}\,:\,\mathsf{QUALM} \text{ implements } \mathsf{Task}\}} \big(\mathsf{GateComplexity}[\mathsf{QUALM}] + \lambda\, \mathsf{QueryComplexity}[\mathsf{QUALM}]\big)\,. \tag{13}$$

For instance, λ could be the number of elementary gates required to construct the lab oracle superoperator.

As usual in computational complexity[79], one is interested in *families* of tasks and QUALMs, where some parameter dictating the *size* of the problem grows to infinity, and we ask how the complexity grows as a function of that parameter.

Remark 6 (*Approximate complexity).* As mentioned in Remark 4, one can talk about approximations and errors in QUALMs. The approximate QUALM gate complexity, approximate QUALM query complexity, and approximate QUALM complexity of a task, respectively, are then clearly defined using Definition 12, taking the minimum over all QUALMs which achieves the task to the desired approximation.

Supplementary Note 2.4: Different types of access to a lab oracle

Towards clarifying the power of different QUALMs in terms of their computational abilities, we consider different types of *accesses* of QUALMs to a lab oracle. The most natural (though presently least realistic) one is the access of a full quantum computer. By this we mean a general QUALM, without the restrictions to be specified shortly. In particular, the set of admissible gates $\mathscr{G}$ can be a universal set, and there is no restriction on which gates can be applied at any given time.

Definition 13 (Coherent access). We will refer to a general QUALM (i.e., with a universal gate set) defined in Definition 8 as a QUALM with *coherent access* to the lab oracle.

In realistic physics experiments, access is often far more limited. The experimental setup may not be able to introduce quantum entanglement between the physical system $\mathbf{N}\otimes\mathbf{L}$ and the rest of the lab $\mathbf{W}$. Measurements to the system may destroy the quantum coherence in the physical system or even completely destroy the quantum state. In the QUALM framework this is captured by restrictions on the admissible gate sets and the allowed sequences of gates. To make concrete progress we consider a model of many contemporary experiments which we call *incoherent access*. To this end, we need to recall the definition of a one-round LOCC protocol between two parties (see, e.g.,[22]). First, we recall that a map $\mathscr{N}^{\mathbf{R}}$ on density matrices on the register $\mathbf{R}$ is completely positive (CP for short) if it can be associated with a set of Kraus operators $\{\mathscr{A}_\alpha\}_\alpha$ such that for all ρ on $\mathbf{R}$

$$\mathscr{N}^{\mathbf{R}}(\rho) = \sum_\alpha \mathscr{A}_\alpha \rho \mathscr{A}_\alpha^\dagger\,.$$

Furthermore, $\mathscr{N}^{\mathbf{R}}$ is trace-preserving if

$$\sum_\alpha \mathscr{A}_\alpha^\dagger \mathscr{A}_\alpha = \mathbb{1}\,.$$

A completely positive trace-preserving (CPTP) map is often called, simply, a quantum channel.

Now we specify a quantum channel that implements a certain kind of communication between subsystems $\mathbf{A}$ and $\mathbf{B}$ called a “one-round LOCC” (see[22]).

Definition 14 (*One-round LOCC).* A *one-round LOCC operator* from subsystems $\mathbf{A}$ to $\mathbf{B}$ is a quantum channel (i.e. a CPTP map) $\mathscr{E}^{\mathbf{AB}}$ acting on $\mathscr{D}(\mathbf{A}\otimes\mathbf{B})$, which is of the form

$$\mathscr{E}^{\mathbf{AB}}(\cdot)=\sum_{\alpha}\mathscr{M}_{\alpha}^{\mathbf{A}}(\cdot)\otimes\mathscr{N}_{\alpha}^{\mathbf{B}}(\cdot) \tag{14}$$

with $\mathscr{M}_{\alpha}^{\mathbf{A}}$ a completely positive (CP) map acting on the $\mathbf{A}$ subsystem, and $\mathscr{N}_{\alpha}^{\mathbf{B}}$ a completely positive and trace-preserving (CPTP) map acting on the $\mathbf{B}$ subsystem.

It should be noted that this definition in fact requires $\mathscr{E}^{\mathbf{A}}\equiv\sum_{\alpha}\mathscr{M}_{\alpha}^{\mathbf{A}}$ to be a CPTP map (instead of merely a CP map), since $\mathscr{E}^{\mathbf{A}}$ is the reduced channel obtained by tracing over $\mathbf{B}$ in $\mathscr{E}^{\mathbf{AB}}$.

With the above definition in mind, we can now define incoherent access QUALMs:

Definition 15 (*Incoherent access).* We say a QUALM uses *incoherent access* to the lab oracle if the following holds for the sequence of symbols $\mathscr{Q}$. Let k be the number of times the symbol $\square$ appears in $\mathscr{Q}=(\mathscr{Q}_1,\mathscr{Q}_2,...,\mathscr{Q}_{\text{final}})$. As usual, we associate $\mathscr{Q}$ with the channel $\mathscr{Q}=\mathscr{Q}_{\text{final}}\circ\cdots\circ\mathscr{Q}_2\circ\mathscr{Q}_1$, and regroup terms as

$$\mathscr{Q}=\mathscr{C}_k\circ\square\circ\mathscr{C}_{k-1}\circ\square\circ\cdots\circ\square\circ\mathscr{C}_0$$

where $\mathscr{C}_i$ is a the sequence of gates applied after the ith call to the lab oracle, and before the $(i+1)$st one. We require that

- For each $i\in\{0,...,k\}$, the sequence of admissible gates $\mathscr{C}_i$ can be written as an r_i-round LOCC protocol, for some finite number of rounds r_i. In other words, $\mathscr{C}_i$ can be written as a composition $\mathscr{C}_i=\mathscr{C}_{i,r_i}\circ\cdots\circ\mathscr{C}_{i,2}\circ\mathscr{C}_{i,1}$ such that for every j, $\mathscr{C}_{i,j}$ is a one-round LOCC channel. Without losing generality, we can assume $\mathscr{C}_{i,j}$ alternates between $\mathbf{L}$-to-$\mathbf{W}$ and $\mathbf{W}$-to-$\mathbf{L}$ one-round LOCC channels, since the composition of two $\mathbf{L}$-to-$\mathbf{W}$ one-round LOCC's is still a one-round LOCC.
- Moreover we also require that for each i there exists at least one index $j\in\{1,...,r_i\}$ such that $\mathscr{C}_{i,j}$ is a complete measurement, which is a special one-round LOCC operator from $\mathbf{L}$ to $\mathbf{W}$, as follows:

$$\mathscr{C}_{i,j}(\cdot)=\sum_{\alpha\in\{0,1\}^{\ell}}\mathscr{M}_{\alpha}^{\mathbf{L}}(\cdot)\otimes\mathscr{N}_{\alpha}^{\mathbf{W}}(\cdot), \tag{15}$$

  such that $\mathscr{M}_{\alpha}^{\mathbf{L}}$ is a rank one projection for each α, i.e., $\mathscr{M}_{\alpha}^{\mathbf{L}}(\rho)=|\psi_\alpha\rangle\langle\psi_\alpha|\langle\psi_\alpha|\rho|\psi_\alpha\rangle$ for some pure state $|\psi_\alpha\rangle$, and $\{|\psi_\alpha\rangle\}_{\alpha\in\{0,1\}^{\ell}}$ is an orthonormal basis for $\mathbf{L}$.

The above definition roughly means that the interaction between the $\mathbf{L}$ and $\mathbf{W}$ registers is an LOCC throughout the QUALM protocol; moreover, between any two applications of the lab oracle, the lab register $\mathbf{L}$ is measured using a complete measurement. No coherence can be generated between the state generated by a given single call to the lab oracle, and any other register used by the QUALM – this is the source for the term "incoherent access QUALM".

We note that the above definition allows *adaptive* access to the lab oracle; namely, the state of the register $\mathbf{L}$ before an application of the lab oracle may depend on previous measurement results both of $\mathbf{L}$ and of $\mathbf{W}$, and those in turn may depend on the lab oracle.

Supplementary Note 2.5: Examples of Quantum Tasks

Before turning to new and novel tasks and QUALMs, we give examples of how standard quantum algorithms, along with quantum experimental protocols, can be cast as tasks and QUALMs. This is interesting mainly for quantum experimental protocols, which are not themselves quantum algorithms. However, we begin by showing how quantum algorithms are a special case of quantum tasks and QUALMs:

Example 1: Quantum algorithms. We have discussed this example above, but we repeat it here for completeness. Consider a $\mathsf{Task}=(\mathbf{S}_{\text{in}},\mathbf{S}_{\text{out}},f,\mathscr{G})$ where $\mathscr{G}$ is a universal quantum gate set. We further let $\mathbf{N},\mathbf{L}\simeq\mathbb{C}$ be trivial subsystems, and choose f to be

$$f:\{\mathscr{I}\}\times\{0,1\}^{p}\longrightarrow\{0,1\}^{q} \tag{16}$$

with $|\mathbf{S}_{\text{in}}| = p, |\mathbf{S}_{\text{out}}| = q$, and $\mathscr{I}$ is the trivial lab oracle. Then a QUALM achieving this Task is a quantum algorithm computing the function f.

Next, we turn to procedures like quantum tomography; we will focus on the state setting in particular.

Example 2: Quantum state tomography. Here we consider quantum state tomography on states with ℓ qubits (i.e., the size of the lab register $\mathbf{L}$). We further suppose that $|\mathbf{N}| = n = 0$ and $|\mathbf{L}| = \ell$. The lab oracle LO_ρ is defined to be a channel which takes whatever state is on $\mathbf{L}$ and turns it into ρ. Formally it is defined by $\mathsf{LO}_\rho = (\{\}, \mathscr{E}_{\mathbf{L},\rho})$, where we do not specify a state on $\mathbf{N}$ since the subsystem is empty, and $\mathscr{E}_{\mathbf{L},\rho}$ only acts on $\mathbf{L}$ where for any $\sigma \in \mathscr{D}(\mathbf{L})$ we have

$$\mathscr{E}_{\mathbf{L},\rho}[\sigma] = \operatorname{tr}(\sigma)\,\rho\,. \tag{17}$$

Accordingly, each application of $\mathscr{E}_{\mathbf{L},\rho}$ produces a copy of ρ on $\mathbf{L}$.

To model quantum tomography on a general ℓ qubit state up to error ε in the 1-norm, we consider first a set of states $\rho_1, ..., \rho_m$ with $m = \Omega(1/\varepsilon^{(4^\ell)})$ which forms an epsilon net in $\mathscr{D}(\mathbf{L})$; that is, for any ℓ qubit state ρ, there is a state ρ_i in the net such that $\|\rho - \rho_1\|_1 \leq \varepsilon$.

Then consider $\mathsf{Task} = (\mathbf{S}_{\text{in}}, \mathbf{S}_{\text{out}}, f, \mathscr{G})$ where $|\mathbf{S}_{\text{in}}| = 0$, $|\mathbf{S}_{\text{out}}| = q = \lceil \log_2(m) \rceil$, and $\mathscr{G}$ is a universal gate set. We further have

$$f : \{\mathsf{LO}_\rho\}_{\rho \in \mathscr{D}(\mathbf{L})} \longrightarrow \{0,1\}^q \tag{18}$$

such that for $f(\mathsf{LO}_\rho) = k$ we have $\|\rho - \rho_k\|_1 \leq \varepsilon$ (where the output string $k \in \{0,1\}^q$ is interpreted as a binary representation of an integer between 1 and m).

In fact, the task defined above is non-physical as it requires infinite precision. This is because the function f *partitions* the set $\mathscr{D}(\mathbf{L})$ according to the value k of f. No matter how this partition is defined, there will always be two arbitrarily close states such that the values of the function defined on them are different.

To circumvent this problem, one can restrict the input states to be just those states far from the boundaries of this partition. However, a more natural approach which is closer to physics is to define the state tomography task using a probabilistic function f; namely, f outputs a *distribution* over q-bit strings:

$$f : \{\mathsf{LO}_\rho\}_{\rho \in \mathscr{D}(\mathbf{L})} \longrightarrow \mathscr{D}\{\{0,1\}^q\} \tag{19}$$

Further, we require that for any ρ and any $k \in \{0,1\}^q$ we have

$$\Pr_{k \sim f(\mathsf{LO}_\rho)}[\|\rho - \rho_k\|_1 \leq \varepsilon] \geq 1 - \delta\,. \tag{20}$$

This defines the task of state tomography to within approximation ε, with error δ (here $1-\delta$ can be referred to as the *confidence*).

In a very similar way we can describe quantum channel tomography; we will not flesh it out here.

Now we examine more elaborate protocols, including quantum cooling and quantum communication.

Example 3: Quantum cooling protocols. The following is an example of the more general version of a QUALM, where the output can be a quantum state. Suppose we have a set of Hamiltonians $\{H_1, H_2, ..., H_m\}$ on ℓ qubits and corresponding Gibbs states at temperature T, namely $\{\rho_1(T), \rho_2(T), ..., \rho_m(T)\}$. We let $\mathbf{N} \simeq \mathbb{C}$ (i.e., it is trivial), and consider a family of lab oracles $\mathsf{LO}_k = (\{\}, \mathscr{S}_{\mathbf{L},k})$ where

$$\mathscr{S}_{\mathbf{L},k}(\sigma_{\mathbf{L}}) = \rho_k(T) \tag{21}$$

for any $\sigma_{\mathbf{L}}$. That is, the lab oracle superoperator prepares the Gibbs state $\rho_k(T)$ on $\mathbf{L}$.

Then the cooling Task is as follows. Suppose that $\mathbf{W}$ contains a subsystem $\mathbf{W}_1$ comprising a qudit with orthonormal basis $\{|1\rangle, |2\rangle, ..., |r\rangle\}$, and another subsystem $\mathbf{W}_2$ with the same number of qubits as $\mathbf{L}$. Then we let $\mathbf{S}_{\text{in}} = \mathbf{W}_1$, $\mathbf{S}_{\text{out}} = \mathbf{W}_2$, $\mathscr{G}$ be a universal gate set, and

$$f : \{\mathsf{LO}_1, \mathsf{LO}_2, ..., \mathsf{LO}_m\} \times \{1, 2, ..., r\} \longrightarrow \mathscr{D}(\mathbf{W}_2) \tag{22}$$

where

$$f(\mathsf{LO}_k, s) = \rho_k(T/s)\,. \tag{23}$$

That is, the task is asking for us to output the Gibbs state cooled to $1/s$ of its initial temperature.

Notice that this task is defined *without* access to the Hamiltonian inducing the Gibbs state. Interestingly,[25] offers an efficient QUALM for a weaker version of this task, namely computing expectation values of local observables with respect to the reduced temperature Gibbs state. Related protocols in the context of quantum error correction are[58,66].

Example 4: Quantum communication protocols. Communication complexity[94] is a well-known model in theoretical computer science, in which one considers the following setting. There are two parties, Alice and Bob, and a function $F : \{0,1\}^p \times \{0,1\}^q \longmapsto \{0,1\}$, which is fixed in advance (e.g., if $p = q$ then F can be the equality function between on bit strings). Each of the parties receives an input (x and y respectively) without the other party knowing its value. Alice and Bob now run a "protocol" in which they send bits (or qubits) back and forth between them. At the end of the protocol, one of them (say, Bob) is expected to hold the value $F(x,y)$. The communication complexity of the function is the minimal number of bits (in the quantum case, qubits) which they need to send to each other in order to achieve this goal.

We describe a way to present the communication complexity model in the QUALM framework. The communication complexity turns out to be equal to the QUALM query complexity.[3]

Let $\mathbf{N}$ be an empty subsystem, let the lab decompose as $\mathbf{L} \simeq \mathbf{L}_A \otimes \mathbf{L}_B$ where $\mathbf{L}_A$ and $\mathbf{L}_B$ are each single-qubit subsystems, and let $\mathbf{W} \simeq \mathbf{W}_A \otimes \mathbf{W}_B$ where $|\mathbf{W}_A| = |\mathbf{W}_B| = w/2$ for w even. We can take w to be very large. Our task is denoted by Task_F, and is labelled by a function $F : \{0,1\}^p \times \{0,1\}^q \to \{0,1\}$. We take $\mathbf{S}_{\text{in}}$ to be the union of the first p qubits of $\mathbf{W}_A$ with the first q qubits of $\mathbf{W}_B$, and take $\mathbf{S}_{\text{out}}$ to be the first qubit of $\mathbf{W}_B$. Our admissible gate set $\mathscr{G}$ is *not* universal; rather, it is the union of a universal gate set on $\mathbf{L}_A \otimes \mathbf{W}_A$ with a universal gate set on $\mathbf{L}_B \otimes \mathbf{W}_B$. If we imagine $\mathbf{L}_A \otimes \mathbf{W}_A$ as belonging to Alice and $\mathbf{L}_B \otimes \mathbf{W}_B$ as belonging to Bob, then they can do arbitrary quantum computation on their own domains, but cannot directly share information with one another. To allow Alice and Bob to communicate, we consider a lab oracle $\mathsf{LO}_{\text{SWAP}}$ on $\mathbf{L}_A \otimes \mathbf{L}_B$ which simply swaps the two registers. Then the function f corresponding to Task_F is

$$f : \{\mathsf{LO}_{\text{SWAP}}\} \times \{0,1\}^p \times \{0,1\}^q \longrightarrow \{0,1\} \tag{24}$$

where

$$f(x,y) = F(x,y) \tag{25}$$

for $x \in \{0,1\}^p$ and $y \in \{0,1\}^q$. We have suppressed the dependence on $\mathsf{LO}_{\text{SWAP}}$ since there are no other lab oracles to choose from.

In less mathematical terms, the task is as follows. Alice and Bob are each given bit strings x and y, respectively, and desire to compute a function $F(x,y)$ which Bob is to output. Alice and Bob can only communicate one qubit at a time via the lab oracle. The QUALM query complexity of the task is the same as the quantum communication complexity of the task.[4]

In B, we discuss the slightly more elaborate example of verifying the correct evolution of a quantum computer.

To the best of our knowledge, all examples of quantum experiments (including all those mentioned in the introduction) can be cast in the QUALM framework. Two additional interesting examples which we will mention here without

---

[3]There is another, possibly more straightforward, way to embed the model in the QUALM framework. Let Alice be represented by $\mathbf{L}$ and let Bob be represented by $\mathbf{W}'$ (which is part of the working space $\mathbf{W}$). There is also a single qubit "message" register $\mathbf{M}$ included in $\mathbf{W}$. Gates are not allowed to act jointly on qubits from both $\mathbf{W}'$ and $\mathbf{L}$. The communication protocol can be viewed as a computation, alternating between a computation on $\mathbf{L} \otimes \mathbf{M}$, and a computation on $\mathbf{W}' \otimes \mathbf{M}$. The communication complexity of the protocol is then simply the number of such computation steps; however, in this presentation, the communication complexity is not related to the QUALM query complexity or the QUALM gate complexity.

[4]Technically, our particular construction here counts a bit swap between Alice and Bob as a single operation. However, in standard quantum communication complexity, Alice sending a qubit to Bob is counted as one operation, and Bob sending a qubit to Alice is counted as one operation. Of course, our factor of two mismatch does not affect the asymptotic scaling of the complexity. If one desires, the factor of two can be remedied by making the task setup slightly more sophisticated.

going into explicit detail are control theory and imaging. Quantum tasks lend themselves well to quantum control theory problems (see e.g.[32]). The system to be controlled is regarded as part of Nature, and we can only interact with it or *control* it via the lab oracle superoperator; this couples Nature to *control parameters* in the lab. The lab oracle superoperator may also produce some 'readout' of the Nature system by 'printing' bits on a lab subsystem. This way, each time we utilize the lab oracle superoperator, we can read out some property of the present state of Nature which may influence our subsequent choices of control parameters. It is also natural to cast imaging tasks into the QUALM framework, along similar lines as Figure 1(a). For imaging, the Nature register is the object to be measured, and the lab register contains the equipment that directly interacts with the object (for example, the cantilever and tip of an atomic force microscope). The lab oracle specifies (i) the state of the object, and (ii) the superoperator that controls the dynamics of the object and its interaction with the lab register. Depending on the imaging technique, the interaction can occur by exchanging photons or electrons, or via a static electromagnetic field. The signal received by the lab register is then post-processed to obtain the output image in the working memory register. In most present-day experiments, the post-processing is classical, but in general it could be quantum. The task is a function that maps the (unknown) state and/or dynamics of the object to a desired output image that captures some designated features of the object.

Supplementary Note 3: Exponential advantage for coherent access

In this section, we construct a quantum task for which a coherent access QUALM has an exponential advantage over an incoherent access QUALM. That is, we (i) specify a coherent access QUALM which achieves the task with a number of elementary gates linear in the number of qubits, and (ii) prove that incoherent access QUALMs require *exponentially* many elementary gates in the number of qubits to achieve the task. Note that (ii) is by far the most difficult part of our arguments, as we must prove the *non-existence* of subexponential incoherent access QUALMs for our quantum task at hand.

The content of this section provides a proof of principle that coherent access QUALMs can have a significant advantage over incoherent access QUALMs, even if the latter are adaptive; accordingly some physical experiments may be made vastly more efficient by interfacing with a quantum computer – possibly even with a very simple one. This result represents a novel kind of *exponential advantage* afforded by quantum computation to speed up physics experiments. We discuss related examples to our proof of principle in Sections 4 and 5.

Supplementary Note 3.1: Problem definition and statement of results

Here we will study the problem of distinguishing between (i) many copies of a fixed, Haar random unitary, and (ii) many i.i.d. Haar random unitaries. While the task of distinguishing (i) and (ii) may seem slightly abstract, it is a toy version of a sensible experimental problem: given an experimental system, determine if its dynamics is time-translation invariant or stochastic. A system with discrete time translation symmetry is known as a Floquet system. If the time translation period is T, the time translation operator for time interval $[kT,(k+1)T]$ is a unitary U independent of the integer k. By contrast, a stochastic system such as a random quantum circuit[53] or a Brownian circuit (e.g.[69]) has a time-dependent evolution operator U_k for the same time interval, which depends on some classical random variables that are uncorrelated for different time intervals. The toy model we consider corresponds to a highly chaotic case, where we assume the U for the Floquet system is Haar random, and the U_k's for stochastic system are i.i.d. Haar random.

We begin by defining the task of distinguishing between two lab oracles, and then define our precise lab oracles of interest.

Definition 16 (*The task of distinguishing between two lab oracles*). Consider two lab oracles $\mathsf{LO}_0 = (\mathscr{E}_{\mathbf{NL}},\rho_{\mathbf{N}})$, $\mathsf{LO}_1 = (\mathscr{E}'_{\mathbf{NL}},\rho'_{\mathbf{N}})$, and a quantum task $(\mathbf{S}_{\text{in}},\mathbf{S}_{\text{out}},f,\mathscr{G})$. Here $\mathbf{S}_{\text{in}}$ is empty, $\mathbf{S}_{\text{out}}$ consists of a single qubit, $\mathscr{G}$ is any universal set of quantum gates, and

$$f:\{\mathsf{LO}_0,\mathsf{LO}_1\}\to\{0,1\}\,,$$

where $f(\mathsf{LO}_0)=0$ and $f(\mathsf{LO}_1)=1$.

By Definition 10, a QUALM achieves the above task with error at most ε if its output density matrix (in the present case, on a single qubit) is within ε in trace distance from the correct one.

Definition 17 (*A QUALM distinguishing between two lab oracles with a bias δ*). Suppose we have a QUALM with a single qubit output, $|\mathbf{S}_{out}| = 1$, which outputs the density matrix ρ_0 or ρ_1 when the lab oracle is LO_0 or LO_1, respectively. We say that the QUALM distinguishes between the two lab oracles with bias δ if the trace distance between its two output density matrices is at least δ:

$$\|\rho_0 - \rho_1\|_1 \geq \delta\,. \tag{26}$$

Remark 7 (*From biases of single qubit density matrices to deterministic functions*). In the remainder of the paper, when we consider QUALMs performing tasks which distinguish between lab oracles, we work with the more general Definition 17 and thus incorporate biases δ into our analyses. Note that given a QUALM which distinguishes between the two lab oracles with bias $\delta = \Omega(1)$, we can by repetition and classical computation (as in Remark 4) construct a new QUALM' which achieves the task in Definition 16, i.e. which implements $f(\mathsf{LO}_0) = 0$ and $f(\mathsf{LO}_1) = 1$ with error less than ε (where ε is any inverse polynomial in ℓ of our choice). As long as δ is inverse polynomial in ℓ, the QUALM query and gate complexities are just multiplied by some polynomial factor in ℓ.

Notice that the above is true since we require in Definition 17 that the output is a single qubit. This is done to avoid the following situation. Suppose we have a bigger $\mathbf{S}_{\text{out}}$ and the two output states ρ_0, ρ_1 have a bias δ' in the 1-norm, namely $\|\rho_0 - \rho_1\|_1 \geq \delta'$. Then there exists an operator on $\mathbf{S}_{\text{out}}$ that, when measured, can distinguish the two output states with this bias. However, for a large $\mathbf{S}_{\text{out}}$ the complexity of finding such an operator and then carrying out the measurement can be large. As such, requiring the output to be only a single qubit ensures that the QUALM gate complexity in Definition's 11 and 12 accounts for the complexity of the channels used for the readout.

For our purposes, we now consider two specific lab oracles: LOP_ℓ and LOQ_ℓ. We will give precise definitions of these lab oracles shortly, but first we explain what they are in words. Here LOP_ℓ is a lab oracle whose quantum channel applies a new Haar random unitary to ℓ bits in $\mathbf{L}$ each time the oracle is called (i.e., for each application of the channel a new Haar random unitary is picked independently from scratch). By contrast, LOQ_ℓ is a lab oracle whose quantum channel implements a single Haar random unitary U, i.e. the same U is applied each time the oracle is called. We now make these definitions of the lab oracles more explicit:

Definition 18 (*The fixed random unitary lab oracle LOQ_ℓ*). For ℓ a positive integer, we define the lab oracle $\mathsf{LOQ}_\ell = (\mathscr{E}_{\mathbf{NL}}, \rho_{\mathbf{N}})$ as follows:

- $\mathbf{N}$ is an $e^{\mathscr{O}(\ell)}$ qubit Hilbert space,
- $\mathbf{L}$ is an ℓ qubit Hilbert space,
- $\mathscr{E}_{\mathbf{NL}}$ is a unitary transformation such that, for U a unitary on ℓ qubits and $|U\rangle$ a classical description of U on $e^{\mathscr{O}(\ell)}$ qubits (specified to within exponentially many bits of accuracy in each entry),

$$\mathscr{E}_{\mathbf{NL}}(|U\rangle\langle U| \otimes |\alpha\rangle\langle\alpha|) = |U\rangle\langle U| \otimes U|\alpha\rangle\langle\alpha|U^\dagger\,,$$

- $\rho_{\mathbf{N}} = \int_{\text{Haar}} |U\rangle\langle U|\, dU\,.$

We likewise have:

Definition 19 (*The newly chosen random unitary lab oracle* LOP_ℓ). For ℓ a positive integer, define $\mathsf{LOP}_\ell = (\mathscr{E}'_{\mathbf{NL}}, \rho'_{\mathbf{N}})$ as follows.

- $\mathbf{N}$ is an $n \sim e^{\mathscr{O}(\ell)}$ qubit Hilbert space,
- $\mathbf{L}$ is an ℓ qubit Hilbert space,
- $\mathscr{E}'_{\mathbf{NL}} = \mathscr{E}_{\mathbf{NL}} \circ \mathscr{C}_{\mathbf{N}}$ where

$$\mathscr{C}_{\mathbf{N}}[\rho] = \int_{Haar} |U\rangle\langle U|\, dU$$

for any ρ (i.e., it prepares the same state regardless of the input) where U is a unitary on ℓ qubits, and $|U\rangle$ a classical description of U on n qubits (specified to within exponentially many bits of accuracy in each entry), and $\mathscr{E}_{\mathsf{NL}}$ is the same as in the previous definition, i.e.

$$\mathscr{E}_{\mathsf{NL}}(|U\rangle\langle U|\otimes|\alpha\rangle\langle\alpha|) = |U\rangle\langle U|\otimes U|\alpha\rangle\langle\alpha|U^\dagger,$$

- $\rho'_{\mathsf{N}} = |0\rangle\langle 0|^{\otimes n}$.

We note that the above definitions provide only approximations of the unitaries U involved, due to finite precision following from the finite number of bits in the description of the unitary; since there is no limit on the number of bits in N, this introduces an arbitrarily small error, which does not affect any of our calculations. We ignore this in our discussion.

With the definitions of LOP_ℓ and LOQ_ℓ at hand, it is easy to establish the following theorem:

Theorem 3 (Coherent access QUALM that probabilistically distinguishes LOP_ℓ from LOQ_ℓ). There exists a family of coherent access QUALMs, $\{\mathsf{QUALM}_\ell\}_{\ell=1}^{\infty}$, over universal admissible gate sets such that QUALM_ℓ distinguishes between the lab oracles LOP_ℓ and LOQ_ℓ with error $< 1/3$. Furthermore, this is achieved with $\mathscr{O}(\ell)$ QUALM gate complexity and $\mathscr{O}(1)$ QUALM query complexity.

Proof. The idea is to implement the familiar SWAP test between two applications of the lab oracle, using a control qubit. This gives a QUALM which always outputs 1 in the LOQ_ℓ case, but its probability to output 1 in the LOP_ℓ case is less than $2/3$. Thus, the QUALM achieves the task with bias $\geq \frac{1}{3}$. (This can then be amplified using standard repetition as in Remark 4 to obtain a QUALM achieving the distinguishing Task of Definition 16 with error $< \frac{1}{3}$.) We now describe the QUALM.

The coherent access QUALM we construct has no input classical bits: namely, S_{in} is empty. W is the union of register W_1 consisting of ℓ qubits, and W_2 consisting of a single qubit called the "control qubit". S_{out} consists of the single qubit in W_2.

The series of gates $\mathscr{Q}$ is as follows. We (i) apply the lab oracle superoperator on $\mathsf{N}\otimes\mathsf{L}$, (ii) swap the additional register and L and again apply the lab oracle, and (iii) finally apply $\text{SWAP}_{\text{TEST}}$ on the two ℓ qubit registers using the control qubit:

$$\mathscr{Q} = \text{SWAP}_{\text{TEST}}(\mathsf{L},\mathsf{W}_1)\circ\square\circ\prod_{i=1}^{\ell}\text{SWAP}_{i,\ell+i}\circ\square\,. \tag{27}$$

The channel $\text{SWAP}_{\text{TEST}}(\mathsf{L},\mathsf{W}_1)$ consists of first applying the Hadamard gate on the control qubit to bring it to the state $|+\rangle$; then conditioned on the control qubit being 1, the ith qubit in register L is swapped with the ith qubit in the additional register, for all i from 1 to ℓ. Then we apply another Hadamard on the control qubit W_2.

Let us calculate the distribution on W_2 in each of the cases. In the case the lab oracle is LOP_ℓ, the state of the L and W registers (namely the first 2ℓ qubits) just before the application of the SWAP test is a mixture over $U\left|0^\ell\right\rangle\otimes U'\left|0^\ell\right\rangle$ with U,U' being independently chosen Haar random ℓ qubit unitaries. In the case the lab oracle is LOQ_ℓ, the state will be a mixture over $U\left|0^\ell\right\rangle\otimes U\left|0^\ell\right\rangle$ with U being a Haar random ℓ qubit unitary. By simple algebra, the probability for the output to be 0 after applying the SWAP test on the tensor product state $|\alpha\rangle\otimes|\beta\rangle$ is

$$\frac{1+|\langle\beta|\alpha\rangle|^2}{2}.$$

In the case the lab oracle is LOQ_ℓ, this is always 1 (for any state $U\left|0^\ell\right\rangle\otimes U\left|0^\ell\right\rangle$ in the mixture); in the case the lab oracle is LOP_ℓ , the state of the L and W registers is a mixture over $|\alpha\rangle\otimes|\beta\rangle$ with $|\alpha\rangle, |\beta\rangle$ being two independently chosen Haar random ℓ qubit states. We recall a useful version of Levy's inequality:

Lemma 1 (Levy's inequality[70,72]). For any fixed unit vectors $|\alpha\rangle$ and $|\beta\rangle$ on ℓ qubits, we have

$$\text{Prob}_{\text{Haar}}\left(\left||\langle\alpha|U|\beta\rangle|^2-\frac{1}{D}\right|\geq\varepsilon\right)\leq 4\,e^{-D\varepsilon^2/(18\pi^2)} \tag{28}$$

where $D = 2^\ell$.

Levy's inequality implies that (for sufficiently large ℓ) the probability for the inner product between two Haar random states to be $< \frac{1}{100}$ is doubly exponentially close to 1. Thus the probability to get 0 in the SWAP test (averaged over the Haar random choices) is $> 1/3$.

Since the bias of this QUALM, which uses two queries to the lab oracle, is constant, only constantly many repetitions are needed. Thus, the QUALM query complexity of the amplified QUALM is indeed a constant. The QUALM gate complexity of the final amplified QUALM is a constant times that of the gate complexity of the above QUALM, which is $\mathcal{O}(\ell)$ due to the SWAP gates and Hadamards; to this we need to add the postprocessing of counting the constantly many output bits to decide on the outcome, which only requires constantly many gates. This gives a total gate complexity of $\mathcal{O}(\ell)$. □

We now consider the case of incoherent access QUALMs. Our first main result is:

Theorem 4 (Incoherent access QUALMs cannot efficiently distinguish LOP_ℓ from LOQ_ℓ). Consider an incoherent access QUALM for the task of distinguishing LOP_ℓ and LOQ_ℓ, which makes k queries to the lab oracle, such that $k < \left(2^\ell/\sqrt{6}\right)^{4/7}$. Then (i) *the output single qubit density matrices on* $\boldsymbol{S}_{out}$ *for the two cases are within trace distance* $\mathcal{O}(k^3/2^\ell)$*;* (ii) *the QUALM thus distinguishes the two lab oracles with bias at most* $\delta = \mathcal{O}(k^3/2^\ell)$*; and* (iii) *consequently, the QUALM query complexity for distinguishing the two lab oracles with constant error (Definition 16) is lower bounded by the exponential* $\Omega(2^{2\ell/7})$.

The above two theorems together imply that coherent access to lab oracle experiments can be exponentially more efficient, even when compared to QUALMs with incoherent *adaptive* access to the lab oracle.

The proof of Theorem 4 is technically heavy. The overall idea is to first consider the output distribution over the measurement outcomes, for all measurements performed when applying the QUALM on the chosen lab oracle. For the two different lab oracles, we arrive at two different distributions; we will show that these are exponentially close in total variation distance. From this it will be easy to deduce that the output density matrices of the QUALM are also exponentially close. However, for the adaptive case, the proof is very complicated. For didactic reasons, we consider first the much simpler case in which the access is non-adaptive (and also further somewhat restricted), and prove it in Subsection 3.2. The general adaptive scenario will be proved in Supplementary Note 3.3.

Supplementary Note 3.2: Proof of a restricted case of Theorem 4: the parallel repetition case

To gain more intuition, in this subsection we will prove Theorem 4 for the following very restricted case. In particular, the QUALM is non-adaptive, and moreover we require that the different applications of the lab oracle are performed in exactly the same simple way: A state $V|0^\ell\rangle$ is prepared on $\mathbf{L}$ for some fixed unitary V, the lab oracle is applied, a fixed unitary Y is applied on $\mathbf{L}$, and finally $\mathbf{L}$ is measured in the computational basis. The outcome density matrix of the QUALM is some deterministic function of all these measurement outcomes. We first write the probability distribution over all the measurement outcomes, in the case the lab oracle is LOP_ℓ or LOQ_ℓ, respectively, as follows. The distributions are over k ℓ-bit strings $x_1,...,x_k$ where each $x_i \in \{0,1\}^\ell$:

$$P_k(x_1,...,x_k;Y,V) = \prod_{i=1}^{k} \int_{\text{Haar}} dU\, |\langle x_i|YUV|0^\ell\rangle|^2 \tag{29}$$

$$Q_k(x_1,...,x_k;Y,V) = \int_{\text{Haar}} dU \prod_{i=1}^{k} |\langle x_i|YUV|0^\ell\rangle|^2 \,. \tag{30}$$

We let $D = 2^\ell$. We prove in Lemma 2 a bound on the total variation distance between the two distributions, as a function of k.

Lemma 2. For any choice of ℓ-qubit unitaries V and Y, the total variational distance between the above two distributions is $\mathcal{O}(k^2 2^{-\ell/4})$.

Note that the above lemma only implies a bound of $\Omega(2^{\ell/8})$ on the QUALM complexity. This is a slightly weaker bound than the one proven in the next subsection for the general case, Theorem 4, but is still exponential.

Proof. We distinguish between the case in which the $\ell \cdot k$-bit string has a collision and the case in which it does not. We say that an $\ell \cdot k$-bit string $(x_1, ..., x_k)$ has a *collision* if there exist two distinct indices $i, j \in \{1, ..., k\}$, $i \neq j$ such that the associated ℓ-bit strings x_i and x_j are equal. Otherwise, we say the $\ell \cdot k$-bit string has *no collision*.

No collision case: We first claim that P_k is *flat* on $\ell \cdot k$-bit strings with no collision, namely, $P_k(x_1, ..., x_k) = P_k(x'_1, ..., x'_k)$ for any two no-collision $\ell \cdot k$-bit strings $(x_1, ..., x_k)$ and $(x'_1, ..., x'_k)$. This is easy to see since P_k is simply the uniform distribution over *all* $\ell \cdot k$-bit strings, and gives all of them the same probability $\frac{1}{2^{\ell k}}$. This follows since by definition, P_k is a product distribution over the different x_i's ; to calculate the distribution over $x_i \in \{0,1\}^\ell$ for a fixed i, recall that the Haar random measure on unitaries is invariant under rotation by a fixed unitary from the left or from the right, hence

$$\int_{\text{Haar}} dU \, |\langle x_i | YUV | 0^\ell \rangle|^2 = \int_{\text{Haar}} dU \, |\langle x_i | U | 0^\ell \rangle|^2 \,. \tag{31}$$

Moreover, if we let A be a unitary which swaps $|x_i\rangle$ with $|x_j\rangle$ and applies the identity on all other computational basis states, then AU is also Haar random, and hence also

$$\int_{\text{Haar}} dU \, |\langle x_i | AU | 0^\ell \rangle|^2 = \int_{\text{Haar}} dU \, |\langle x_j | U | 0^\ell \rangle|^2.$$

We get that all x_i's are equally probable, and

$$P_k(x_1, ..., x_k; Y, V) = \frac{1}{D^k} \,. \tag{32}$$

We now claim that all $\ell \cdot k$-bit strings with no collision are equally probable also with respect to Q_k (in the case of Q_k, unlike for P_k, we do not know this to be true when there are collisions). Consider two such no-collision $\ell \cdot k$-bit strings, $(x_1, ..., x_k)$ and $(x'_1, ..., x'_k)$, namely, $x_1, ..., x_k$ are all distinct, and so are $x'_1, ..., x'_k$. One can easily convince oneself that this implies there exists a permutation on the 2^ℓ ℓ-bit strings which takes x_i to x'_i for all $i \in \{1, ..., k\}$. We now define a unitary A, acting on the Hilbert space of ℓ qubits, which applies exactly the permutation on the computational basis induced by the above permutation on ℓ-bit strings; in particular, it satisfies: $A|x_i\rangle = |x'_i\rangle$ for all i. By definition, and using the fact that A is unitary, we have

$$\langle x_i | A^{-1} = \langle x'_i | \tag{33}$$

for all $i \in \{1, ..., k\}$. We can write

$$Q_k(x_1, ..., x_k; Y, V) = \int_{\text{Haar}} dU \prod_{i=1}^{k} |\langle x_i | A^{-1} AYUV | 0^\ell \rangle|^2 = \int_{\text{Haar}} dU \prod_{i=1}^{k} |\langle x'_i | AYUV | 0^\ell \rangle|^2 \,. \tag{34}$$

Since U is Haar random so is $AYUV$ for any fixed unitaries A, Y, V, and so we get that the above expression is also equal to

$$\int_{\text{Haar}} dU \prod_{i=1}^{k} |\langle x'_i | U | 0^\ell \rangle|^2 = Q_k(x'_1, ..., x'_k; Y, V) \,. \tag{35}$$

This completes the proof that all no-collision $\ell \cdot k$-bit strings are equiprobable with respect to Q_k.

Collision case: We now claim that the total probability for a "collision" is exponentially small in ℓ, for both P_k and Q_k. For P_k the probability for a collision is at most $\binom{k}{2}$ times the probability for a collision between x_i and x_j for some fixed

choice of $i \neq j$; this latter probability is exactly $\frac{1}{2^\ell}$, and hence if we let ε_P to be the total probability mass on all $\ell \cdot k$-bit strings in P_k with collisions, we have

$$\varepsilon_P \leq \binom{k}{2} \frac{1}{2^\ell} . \tag{36}$$

For Q_k, we rely on concentration of measure arguments, and in particular, on Levy's inequality (Lemma 1). Consider the event, which we denote by B, that

$$|\langle x| YUV |0\rangle|^2 < 2^{-\ell/4} \tag{37}$$

for all $x \in \{0,1\}^\ell$ simultaneously. We will make two claims. First, we claim that the event B happens with all but doubly exponentially small probability; second, we claim that conditioned on B, the probability for a collision is exponentially small.

Claim 1. If U is distributed Haar randomly, then $Pr_{U\sim \text{Haar}}(\neg B)$ is doubly exponentially small in ℓ.

Proof. Apply Lemma 1 to the Haar random matrix YUV. The probability that a given coordinate of $YUV|0\rangle$ is more than $\varepsilon(D)$ is $\leq 4\exp(-\Omega(D\varepsilon(D)^2))$. Pick $\varepsilon(D)$ to be $D^{-1/4}$. We get that the chances are $< 4\exp(-\Omega(\sqrt{D}))$ that a particular coordinate would be larger than $D^{-1/4}$. The claim follows by applying a union bound on all $D = 2^\ell$ ℓ-bit strings. □

To bound ε_Q, the total probability for a collision in Q_k, we next upper bound the total probability of all $\ell \cdot k$-bit strings *with* a collision, conditioned that the event B occured, namely that $|\langle x| YUV |0\rangle|^2 < 2^{-\ell/4}$ for all ℓ-bit strings x. For each such U, the probability (now probability is with respect to the quantum measurement, not the choice of U!) for a collision is at most the sum over $i = 2, ..., k$, i.e. over the probability that the next string will be equal to one of the previous $i-1$ chosen strings. By the union bound, this is at most $k^2 2^{-\ell/4}$. Together with Claim 1 we arrive at the following upper bound on ε_Q, the probability for a collision in Q_k:

$$\varepsilon_Q \leq \Pr[\neg B] + \Pr[\text{collision}|B] \leq 2k^2 2^{-\ell/4}. \tag{38}$$

This is sufficient for our purposes.

Bounding the TVD: P_k is thus flat on no-collision $\ell \cdot k$-bit strings, and has at most $\varepsilon_P \leq \binom{k}{2}\frac{1}{2^\ell}$ probability for $\ell \cdot k$-bit strings with collision. Q_k is also flat on no-collision $\ell \cdot k$-bit strings, and has at most $\varepsilon_Q \leq 2k^2 2^{-\ell/4}$ probability for a collision. The TVD between the two distributions is at most

$$\sum_{x \text{ with no collision}} \left| \frac{1-\varepsilon_P}{N} - \frac{1-\varepsilon_Q}{N} \right| + \sum_{x \text{ with collision}} |P_k(x) - Q_k(x)| \leq 2\varepsilon_P + 2\varepsilon_Q \leq 2\left(\binom{k}{2} 2^{-\ell} + k^2 2^{-\ell/4}\right), \tag{39}$$

where N is the number of $\ell \cdot k$ bit strings x without collision.

This completes the proof of Lemma 2. □

We now deduce item (i) in Theorem 4 (in the restricted case of this subsection) from the above lemma, using the following simple fact, whose proof is just the triangle inequality:

Fact 1. Let $\rho : \{0,1\}^m \longmapsto \mathscr{D}(\mathbb{C}^2)$ be a function from m-bit strings to density matrices of a single qubit. Let μ_1, μ_2 be two probability distributions over m bit strings, with total variation distance $\|\mu_1 - \mu_2\|_1$. Then if we define for $i \in \{1,2\}$

$$\rho_i = \sum_x \mu_i(x)\rho(x) ,$$

we have

$$\|\rho_1 - \rho_2\|_1 \leq \|\mu_1 - \mu_2\|_1 .$$

Item (ii) in the theorem then follows from the well-known fact (e.g.,[5]) which states that the total variation distance between output distributions of measurements applied to two density matrices is at most the trace distance between these density matrices. Finally, item (iii) in the theorem follows from Remark 7.

Remark 8 (*Comment on our choice of lab oracles).* The astute reader may wonder why we chose the lab oracle LOQ_ℓ in Theorem 4 to be averaged over Haar random U. One might ask: given Supplementary Equation (37), can we not satisfy it by a single U? We note that the fact that the output probability distribution gives every string a very small probability (and thus the probability of a collision is very small) is not sufficient to imply hardness of the distinguishing task. Consider for example the lab oracle LOQ'_ℓ which provides samples from a uniform distribution over some fixed chosen subset S of $\{0,1\}^\ell$ containing *half* of the ℓ-bit strings (and set $Y = V = \mathbb{1}$ for this example). Note that Supplementary Equation (37) is satisfied. But of course, if we knew the set S, we in fact could solve the problem of distinguishing between the two lab oracles with only $\mathscr{O}(1)$ samples! By just sampling constantly many times, we are likely to get a string not in S, in the case the lab oracle is LOP_ℓ. The proof of Lemma 2 does not go through in this example *despite* that fact that the probability of collision is negligible, because Supplementary Equation (39) does not hold: the TVD is not small since LOQ'_ℓ does not allow sampling from all $\ell \cdot k$-bit strings with no collision, but only from a tiny fraction of them. Technically, our proof does go through for the lab oracle LOQ_ℓ since in this case we pick a Haar random U and all $\ell \cdot k$-bit strings with no collision are allowed. More intuitively and generally, the reason that in our case distinguishing is indeed impossible is that the lab oracle LOQ_ℓ still has an enormous amount of randomness in the choice of U, and thus one cannot tailor the rule of distinguishing in a fixed QUALM in such a manner that fits all the possible unitaries at once.

Supplementary Note 3.3: Proof of Theorem 4

In this subsection we prove our main result, Theorem 4. The proof of this fact consists of two steps. The first step is to study a special case, which we call a 'simple measurement' (SM) QUALM, and show that it cannot distinguish the two lab oracles LOP_ℓ, LOQ_ℓ . The second step is to show that the output of a general incoherent access QUALM can be related to a probabilistic average of those of SM QUALMs, so that a general incoherent access QUALM cannot distinguish the two lab oracles if no SM QUALM can do that.

3.3.1 Simple measurement QUALM

Before providing the rigorous definition of the SM QUALM, we would like to explain the physical intuition. The SM QUALM describes a simple case of an incoherent access QUALM where there is only one measurement carried out after each application of lab oracle. The measurements are of a particular form: each is a POVM in which each element is of rank one. In the ith round, the measurement output s_i is recorded in a new tensor factor of W, denoted by W_i . The ith POVM is thus described by

$$\{\lambda^i_{s_0 s_1 \dots s_i} \left|y^i_{s_0 s_1 \dots s_i}\right\rangle \left\langle y^i_{s_0 s_1 \dots s_i}\right|\}_{s_i}, \text{ where } \sum_{s_i} \lambda^i_{s_0 s_1 \dots s_i} \left|y^i_{s_0 s_1 \dots s_i}\right\rangle \left\langle y^i_{s_0 s_1 \dots s_i}\right| = \mathbb{1}, \;\; 0 < \lambda^i_{s_0 s_1 \dots s_i} \leq 1 \,. \tag{40}$$

We note that both $\left|y^i_{s_0 s_1 \dots s_i}\right\rangle$ and $\lambda^i_{s_0 s_1 \dots s_i}$ depend not only on s_i but also on all previous measurement results $s_0, \dots, s_{i-1}$ or in short, $s_{j<i}$. After the measurement[5], L is prepared into a mixed state $\sigma^i_{s_0 s_1 \dots s_i}$, which again can depend on the previous measurement results $s_1, \dots, s_{i-1}$. After the last measurement, a readout channel $\mathscr{C}_{\text{out}}$ is applied to W, which maps the diagonal density operator of W to a single qubit output state. The more rigorous definition is given in the following.

Definition 20. (Simple Measurement QUALM) The simple measurement QUALM is an incoherent access QUALM $(\mathsf{L}, \mathsf{W}, \mathscr{Q}, \mathsf{S}_{\text{out}})$ with $\mathsf{W} = \mathsf{W}_0 \otimes \mathsf{W}_1 \otimes \cdots \otimes \mathsf{W}_k$ initialized at $\bigotimes_{i=0}^k \left|0^{\mathsf{W}_i}\right\rangle$, and

$$\mathscr{Q} = \mathscr{C}_{\text{final}} \circ \mathscr{Q}_k \circ \square \circ \mathscr{Q}_{k-1} \circ \square \circ \cdots \circ \square \circ \mathscr{Q}_0 \,.$$

[5]Typically in quantum computation, one considers complete projective measurements and deduces from this the general case. However as we will see below, for the purpose of proving Theorem 4 we need to consider measurements that are slightly more general, where all POVM elements are of rank 1, but they do not correspond to projections onto orthogonal states.

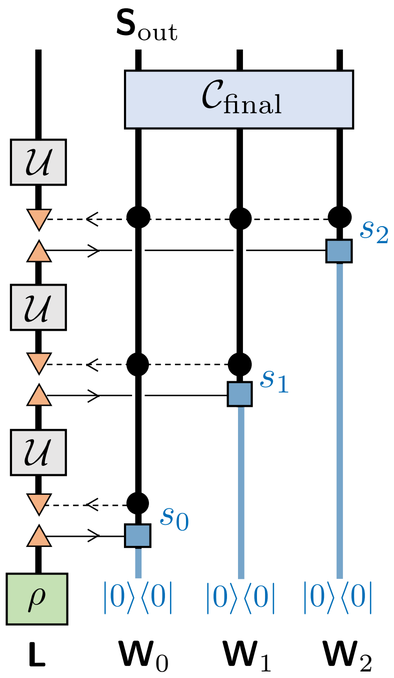

Supplementary Figure 3. *Illustration of the simple measurement QUALM*. This provides a diagrammatic representation of Definition 20. The upward pointing triangles indicate the weighted projection $\rho_{\mathsf{L}} \to \langle y_{s_0,\ldots s_i} | \rho_{\mathsf{L}} | y_{s_0,\ldots,s_i} \rangle \lambda_{s_0,\ldots,s_i}$. The horizontal solid lines indicate the recording of POVM measurement results in W_i. The horizontal dashed lines connected to downward pointing triangles indicate the preparation of initial state $\sigma_{s_0,\ldots,s_i}$ controlled by previous measurement results $s_0,\ldots,s_i$.

Each $\mathscr{Q}_i$ applies to L and $\mathsf{W}_j,\ j \leq i$. Since W_i is initialized to $\left|0^{\mathsf{W}_i}\right\rangle$, we only need to define the action of $\mathscr{Q}_i$ on states of the form $\rho_{\mathsf{L}} \otimes \rho_{\mathsf{W}_{j<i}} \otimes \left|0^{\mathsf{W}_i}\right\rangle\left\langle 0^{\mathsf{W}_i}\right|$:

$$\mathscr{Q}_i\left(\rho_{\mathsf{L}} \otimes \rho_{\mathsf{W}_{j<i}} \otimes \left|0^{\mathsf{W}_i}\right\rangle\left\langle 0^{\mathsf{W}_i}\right|\right) = \sum_{s_0,s_1,\ldots,s_i} \lambda^i_{s_0s_1\ldots s_i} \left\langle y^i_{s_0s_1\ldots s_i}\right|\rho_{\mathsf{L}}\left|y^i_{s_0s_1\ldots s_i}\right\rangle \sigma^i_{s_0s_1\ldots s_i} \otimes K_{s_0s_1\ldots s_{i-1}}\rho_{\mathsf{W}_{j<i}}K_{s_0s_1\ldots s_{i-1}} \otimes \left|s_i^{\mathsf{W}_i}\right\rangle\left\langle s_i^{\mathsf{W}_i}\right| \tag{41}$$

with $s_j = 1,2,\ldots,D_m$ a set of labels for each $j = 0,1,\ldots,k$; $s_{j<i} \equiv \{s_0,s_1,\ldots,s_{i-1}\}$ refers to the previous measurement results;

$$0 < \lambda^i_{s_0s_1\ldots s_i} \leq 1\,; \tag{42}$$

the vectors $\left|y^i_{s_0s_1\ldots s_i}\right\rangle$ and values $\lambda^i_{s_0s_1\ldots s_i}$ satisfy

$$\sum_{s_i} \lambda^i_{s_0s_1\ldots s_i}\left|y^i_{s_0s_1\ldots s_i}\right\rangle\left\langle y^i_{s_0s_1\ldots s_i}\right| = \mathbb{1}\,; \tag{43}$$

$\sigma^i_{s_0s_1\ldots s_i}$ are density matrices of L; $\mathsf{W}_{j<i} \equiv \mathsf{W}_0 \otimes \mathsf{W}_1 \otimes \cdots \otimes \mathsf{W}_{i-1}$; $K_{s_{j<i}} = \bigotimes_{j=0}^{i-1}\left|s_j^{\mathsf{W}_j}\right\rangle\left\langle s_j^{\mathsf{W}_j}\right|$ is the projector to a particular record in $\mathsf{W}_{j<i}$; and $\left|s_j^{\mathsf{W}_j}\right\rangle$ is an orthornormal basis of W_j. Finally, S_{out} is the first qubit in W, and $\mathscr{C}_{\text{final}} : \mathscr{D}(\mathsf{W}) \to \mathscr{D}(\mathsf{W})$ is a general quantum channel.

The definition is illustrated in Supplementary Figure 3. Physically, $\mathscr{Q}_i$ is measuring the state of L with the POVM defined by Supplementary Equation (43) and then based on the outcome s_i, prepares a new state $\sigma^i_{s_0s_1\ldots s_i}$ which is in general a mixed state. The measurement basis and the state prepared depend also on the previous measurement outputs $s_0,s_1,\ldots,s_{i-1}$, which we read from the state of $\mathsf{W}_{j<i}$. It should be noted that the states $\left|y^i_{s_0s_1\ldots s_i}\right\rangle$ are normalized but are not necessarily orthogonal to each other. Due to the condition given in Supplementary Equation (43), in general the range D_m (where 'm' stands for 'measurement') of s_i is greater than or equal to the L Hilbert space dimension $D = 2^\ell$. Without loss of generality, we assume that D_m is the same for each i. If we trace over W, the reduced channel on L is what is known as a measure-and-prepare channel.

For the SM QUALM, the output density operator is determined by applying the readout channel $\mathscr{C}_{\text{final}}$ to the diagonal density operator of W, which encodes the probability distribution of the measurement results $s_0,s_1,\ldots,s_k$. For the fixed random unitary lab oracle LOQ_ℓ (Definition 18), the probability distribution is

$$Q_k\left(s_0,s_1,\ldots,s_k\right) = \Pr(s_0)\cdot\left(\int_{\text{Haar}} dU \prod_{i=1}^{k}\left\langle y^i_{s_0s_1\ldots s_i}\right|U\sigma^{i-1}_{s_0s_1\ldots s_{i-1}}U^\dagger\left|y^i_{s_0s_1\ldots s_i}\right\rangle\lambda^i_{s_0s_1\ldots s_i}\right) \tag{44}$$

with $\Pr(s_0) = |\left\langle y^0_{s_0}\middle|0^{\mathsf{L}}\right\rangle|^2\lambda^0_{s_0}$. The probability distribution for the other lab oracle LOP_ℓ (Definition 19) is

$$P_k\left(s_0,s_1,\ldots,s_k\right) = \Pr(s_0)\left(\prod_{i=1}^{k}\int_{\text{Haar}} dU_i\left\langle y^i_{s_0s_1\ldots s_i}\right|U\sigma^{i-1}_{s_0s_1\ldots s_{i-1}}U^\dagger\left|y^i_{s_0s_1\ldots s_i}\right\rangle\lambda^i_{s_0s_1\ldots s_i}\right). \tag{45}$$

We note that this probability is equal to

$$P_k\left(s_0,s_1,\ldots,s_k\right) = \Pr(s_0)D^{-k}\prod_{i=1}^{k}\lambda^i_{s_i}. \tag{46}$$

In the following we will often denote the ordered sequence $s_0,s_1,\ldots,s_k$ by s for simplicity.

Our conclusion is that these two probability distributions Q_k and P_k are difficult to distinguish, which is given in the following lemma:

Lemma 3. Let Q_k and P_k be defined by Supplementary Equations (44) and (45) above, where $\left|y^i_{s_0 s_1 \ldots s_i}\right\rangle$ are normalized pure states in $\mathbf{L}$, and $\sigma^{i-1}_{s_0 s_1 \ldots s_{i-1}}$ are normalized density operators in the same Hilbert space. We have $0 \le \lambda^i_{s_0 s_1 \ldots s_i} \le 1$ and $\sum_{s_i} \lambda^i_{s_0 s_1 \ldots s_i} \left|y^i_{s_0 s_1 \ldots s_i}\right\rangle \left\langle y^i_{s_0 s_1 \ldots s_i}\right| = \mathbb{1}$ (condition (43)). $\mathbf{L}$ consists of ℓ qubits, and $k < \left(2^\ell/\sqrt{6}\right)^{4/7}$. $s_j = 1, 2, \ldots, D_m$ is a set of labels for each $j = 0, 1, \ldots, k$. Then

$$\|P_k - Q_k\|_1 = \sum_s |P_k(s) - Q_k(s)| \le \mathscr{O}\left(\frac{k^3}{2^\ell}\right). \tag{47}$$

Lemma 3 will be proved in the next subsection. If we let $\rho(s) = \mathrm{tr}_{\overline{\mathbf{S}}_{\mathrm{out}}}\left(\mathscr{C}_{\mathrm{final}}\left[\bigotimes_{i=0}^k \left|s_i^{\mathbf{W}_i}\right\rangle \left\langle s_i^{\mathbf{W}_i}\right|\right]\right)$, the output state for each of the lab oracles LOP_ℓ and LOQ_ℓ (namely the final state on $\mathbf{S}_{\mathrm{out}}$) is

$$\rho^P_{\mathrm{final}} = \sum_s P_k(s)\rho(s), \qquad \rho^Q_{\mathrm{final}} = \sum_s Q_k(s)\rho(s). \tag{48}$$

Based on Fact 1 in the previous section as well as Lemma 3, we obtain $\|\rho^P_{\mathrm{final}} - \rho^Q_{\mathrm{final}}\|_1 \le \|P_k - Q_k\|_1 \le \mathscr{O}\left(\frac{k^3}{2^\ell}\right)$. This proves Theorem 4 in the restricted case of simple measurement QUALMs. After presenting the proof in the next subsection, we will show how to deduce the full theorem from the SM case, in Supplementary Note 3.3.3.

3.3.2 Proof of Lemma 3

We write the probability distribution Q_k in Supplementary Equation (44) as follows:

$$Q_k(s) = \Pr(s_0) \int_{\mathrm{Haar}} dU\, \mathrm{tr}\left(U^{\otimes k} A_s U^{\dagger \otimes k} B_s\right) \tag{49}$$

where

$$A_s = \bigotimes_{i=1}^k \sigma^{i-1}_{s_0 s_1 \ldots s_{i-1}}, \quad B_s = \bigotimes_{i=1}^k \left|y^i_{s_0 s_1 \ldots s_i}\right\rangle \left\langle y^i_{s_0 s_1 \ldots s_i}\right| \lambda^i_{s_0 s_1 \ldots s_i}. \tag{50}$$

Our goal is to prove that $Q_k(s)$ is close to $P_k(s)$ as in Supplementary Equation (45). A simple fact is that

$$P_k(s) = \Pr(s_0) D^{-k} \mathrm{tr}(B_s). \tag{51}$$

The first step is to carry the integration over U using Weingarten functions:

$$\int_{\mathrm{Haar}} dU \left[U^{\otimes k}\right]_{IJ} \left[U^{*\otimes k}\right]_{KL} = \sum_{\sigma, \tau \in S^k} \tau_{KI} \sigma_{LJ} W(\tau\sigma^{-1}, D). \tag{52}$$

Here I, J, K, L label an orthogonal basis in the k-copied Hilbert space. In a tensor product basis (e.g. the computational basis), we can take $I = \{x_1 x_2 \ldots x_k\}$ with $x_j = 1, 2, \ldots, D$ and $j = 1, 2, \ldots, k$. The action of the permutation group elements σ and τ corresponds to the permutation of different Hilbert space copies. For example, for $k = 2$ a pair permutation is defined by $\sigma(|\psi_1\rangle \otimes |\psi_2\rangle) = |\psi_2\rangle \otimes |\psi_1\rangle$. Many useful mathematical properties of the Weingarten functions are known, which will play an essential role in the proof. We will leave the mathematical details of Weingarten functions to C.

Using Supplementary Equation (52), Q_k can be rewritten as (see Subsection C.1)

$$Q_k(s) = \sum_{\sigma, \tau \in S^k} \mathrm{tr}(A_s \sigma)\, \mathrm{tr}(B_s \tau^{-1}) W(\tau\sigma^{-1}, D). \tag{53}$$

The biggest term in this sum corresponds to $\tau = \sigma = \mathbb{1}$ since $\mathrm{tr}(A_s\sigma)$ and $\mathrm{tr}(B_s\tau^{-1})$ are both bounded by 1, and since the Weingarten function is maximal at $\tau\sigma^{-1} = \mathbb{1}$. The sum in Supplementary Equation (53) consists of three kinds of terms: (i) $\tau = \sigma = \mathbb{1}$; (ii) $\tau = \mathbb{1}$, $\sigma \neq \mathbb{1}$; (iii) $\tau \neq \mathbb{1}$. This leads to the following inequality:

$$\begin{aligned} |Q_k(s) - P_k(s)| \le & \left|W(\mathbb{1}, D) - D^{-k}\right| \mathrm{tr}(B_s) + \sum_{\sigma \neq \mathbb{1}} \left|W(\sigma^{-1}, D)\right| |\mathrm{tr}(A_s\sigma)|\, \mathrm{tr}(B_s) \\ & + \sum_{\tau \neq \mathbb{1}} \sum_\sigma \left|W(\tau\sigma^{-1}, D)\right| |\mathrm{tr}(A_s\sigma)| \left|\mathrm{tr}(B_s\tau^{-1})\right|. \end{aligned} \tag{54}$$

Using

$$|\mathrm{tr}(A_s\sigma)| \le 1 \tag{55}$$

we can further bound the right-hand side by

$$|Q_k(s) - P_k(s)| \le \left[\left|W(\mathbb{1},D) - D^{-k}\right| + \sum_{\nu\neq\mathbb{1}} |W(\nu,D)|\right] \mathrm{tr}(B_s) + \sum_{\nu} |W(\nu,D)| \sum_{\tau\neq\mathbb{1}} \left|\mathrm{tr}\left(B_s\tau^{-1}\right)\right| . \tag{56}$$

Carrying out the sum over s and using the fact that $\sum_s B_s = \mathbb{1}$, and $\mathrm{tr}(\mathbb{1}) = D^k$ (where the identity is over the $\ell \cdot k$ qubits on which B_s acts) we obtain the following bound of the 1-norm distance between the two probability distributions:

$$\delta\left(P_k, Q_k\right) \equiv \sum_s |Q_k(s) - P_k(s)| \le c_1 + c_2 T \tag{57}$$

$$c_1 = D^k \left|W(\mathbb{1},D) - D^{-k}\right| + D^k \sum_{\nu\neq\mathbb{1}} |W(\nu,D)| \tag{58}$$

$$c_2 = D^k \sum_{\nu} |W(\nu,D)| \tag{59}$$

$$T = \sum_{\tau\neq\mathbb{1}} \sum_s \left|\mathrm{tr}\left(B_s\tau^{-1}\right)\right| . \tag{60}$$

In the regime $k < \left(2^\ell/\sqrt{6}\right)^{4/7}$, we are going to prove

$$c_1 = \mathscr{O}\left(\frac{k^{7/2}}{D^2}\right) \tag{61}$$

$$c_2 = 1 + \mathscr{O}\left(\frac{k^2}{D}\right) \tag{62}$$

$$T \le \frac{k^3}{D} + \frac{k^2}{D} + \mathscr{O}\left(\frac{k^5}{D^2}\right) . \tag{63}$$

These together will prove Lemma 3, with the dominant term being $c_2 T$.

Supplementary Equations (61) and (62) are based on relatively simple mathematical facts about Weingarten functions (with the condition $k < \left(2^\ell/\sqrt{6}\right)^{4/7}$), which we derive in C. In the main text we will focus on the more nontrivial proof of Supplementary Equation (63). The proof is based on the following inequality for each nontrivial τ:

$$\sum_s \left|\mathrm{tr}\left(B_s\tau^{-1}\right)\right| \le D^{k-\lfloor \frac{L_\tau}{2} \rfloor} . \tag{64}$$

Here L_τ is the total length of nontrivial cycles in τ, and $\lfloor \frac{L_\tau}{2} \rfloor$ is the greatest integer that is less than or equal to $L_\tau/2$. For example, $L_\tau = 2$ corresponds to a single pair transmutation and more generally $L_\tau = k$ indicates that a cyclic permutation of length k is the longest cycle.

We write the above term more explicitly by using the definition of B_s in Supplementary Equation (49):

$$\left|\mathrm{tr}\left(B_s\tau^{-1}\right)\right| = \prod_{i=1}^{k} \left|\left\langle y^{\tau(i)}_{s_0 s_1 \ldots s_{\tau(i)}} \middle| y^i_{s_0 s_1 \ldots s_i}\right\rangle\right| \lambda^i_{s_0 s_1 \ldots s_i} \tag{65}$$

with $\tau(i)$ the index that i is permuted to. (It should be noted that τ only acts on $i = 1, 2, \ldots, k$ since they correspond to measurements following a lab oracle. The state $i = 0$ does not appear in the permutation.) This equation is illustrated in Supplementary Figure 4. The subtlety is that the measurement basis choice can be adaptive, which means $\left|y^i_{s_i}\right\rangle$ generically

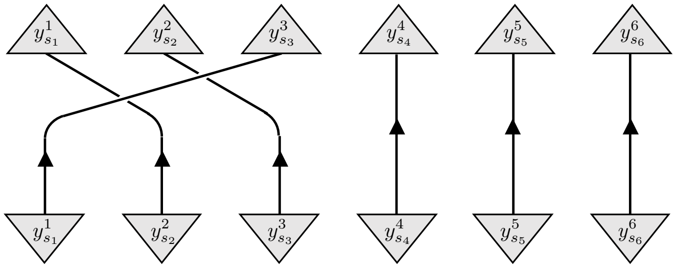

Supplementary Figure 4. *Illustration of an inner product term.* Here we diagrammatically depict the term $\prod_{i=1}^{k}\left\langle y^{\tau(i)}_{s_0 s_1 \ldots s_{\tau(i)}} \middle| y^{i}_{s_0 s_1 \ldots s_i}\right\rangle$ for the example of a cyclic permutation (123). For simplicity of notation we have denoted $y^i_{s_0 s_1 \ldots s_i}$ by $y^i_{s_i}$ in the diagram.

depends on the measurement results s_j for $j < i$ (note that $\left| y^{\tau(i)}_{s_{\tau(i)}} \right\rangle$ may depend on s_j for $j < \tau(i)$). This dependency makes the sum over s_i more difficult than the non-adaptive case.

In the following we denote

$$M_{ji} = \left\langle y^{j}_{s_0 s_1 \ldots s_j} \middle| y^{i}_{s_0 s_1 \ldots s_i}\right\rangle \tag{66}$$

where M_{ji} depends on all the indices s_a for $a \le i$ or $a \le j$. Each permutation τ can be decomposed into cyclic permutations. To illustrate the proof, let us consider an example case. For $n = 8$ we can consider

$$\tau = (175462)(3)(8) \tag{67}$$

which maps 175462 cyclically to 754621 and preserves $3, 8$. For this τ we have

$$\left|\mathrm{tr}\left(B_s \tau^{-1}\right)\right| = |M_{71} M_{57} M_{45} M_{64} M_{26} M_{12}| \prod_{i=1}^{8} \lambda_{s_0 s_1 \ldots s_i}\,. \tag{68}$$

We illustrate a permutation by a curve in Supplementary Figure 5. The τ we consider in Supplementary Equation (67) corresponds to the subfigure (a). The y values of the curve are the site labels 175462, along with an additional 1 returning to the starting point. Each link in this curve which goes from y-coordinates i to j corresponds to a term M_{ji}. When there is more than one loop, we draw a separate curve for each loop. For example, in Supplementary Figure 5(b) we draw the curves for $\tau = (175462)(398)$. The total number of M_{ji} (with $j \neq i$) in $\mathrm{tr}\left(B\tau^{-1}\right)$ is equal to the total length of the nontrivial loops, which we denote as L_τ. For instance, $L_\tau = 6$ for the example in Supplementary Equation (67).

Now we define a partition of the M_{ji} terms, which corresponds to the links in red and blue, respectively. The partition is required to satisfy the following two rules:

1. The number of red links l_r and the number of blue links l_b are different by no more than 1.
2. No segments contain a local maximum (Λ shape).

The second rule means that each local maximum (i.e. cusp points, such as 7 and 6 in Supplementary Figure 5(a)) has to border between red and blue links. By splitting at the cusp points, we obtain "V" shapes. Then we can split each "V" shape into two parts which are only different in length by 0 or ± 1. By choosing the location of the cut, we can make sure that the net length of the red and blue segments are equal (if the total loop length is even) or different by 1 (if the total loop length is odd). Since $l_b + l_r = L_\tau$, without loss of generality we choose

$$l_r = \left\lfloor \frac{L_\tau}{2} \right\rfloor \text{ and } l_b = L_\tau - l_r\,. \tag{69}$$

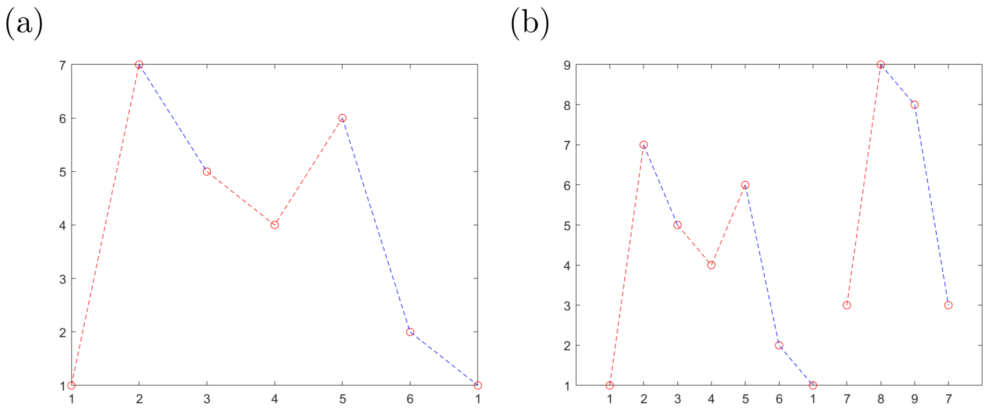

$\tau = (175462)$ $\tau = (175462)(398)$

Supplementary Figure 5. *Illustration of a permutation.* Each cycle in the permutation corresponds to a curve on the figure, starting and ending at the same lowest index. Each link in the figure corresponds to a term M_{ji} in (66), with j, i given by the y value of the two end points. The links can be partitioned into two parts, colored by red and blue. The rules of the partition are that (1) the total length of red and blue links are only different at most by 1; and (2) no segment contains a local maximum (Λ shape).

Now we write

$$\left|\mathrm{tr}(B_s\tau^{-1})\right| = |F_1|\,|F_2|\prod_{i=1}^{8}\lambda_{s_i}^{i} \tag{70}$$

$$F_1 = \prod_{\langle ji\rangle=\mathrm{red}} M_{ji} \tag{71}$$

$$F_2 = \prod_{\langle ji\rangle=\mathrm{blue}} M_{ji} \tag{72}$$

and use the inequality

$$\sum_s \left|\mathrm{tr}(B_s\tau^{-1})\right| = \sum_s |F_1|\prod_{i=1}^{8}\lambda_{s_0s_1\ldots s_i}|F_2| \leq \frac{1}{2}\sum_s\prod_{i=1}^{8}\lambda_{s_0s_1\ldots s_i}\left(|F_1|^2+|F_2|^2\right). \tag{73}$$

For instance, in the example $\tau = (175462)$ with the partition in Supplementary Figure 5(a), we have

$$\begin{aligned} F_1 &= M_{71}M_{64}M_{45} \\ F_2 &= M_{57}M_{26}M_{12}\,. \end{aligned} \tag{74}$$

Now considering $\sum_s |F_1|^2$, we carry out the sum in reverse time order, starting from s_8. The sum over s_8 leads to a factor of D since taking the trace of condition (43) leads to $\sum_{s_i}\lambda^{i}_{s_0s_1\ldots s_i} = D$. Then we can sum over s_7. Since the M_{ji} for $i<7, j<7$ are independent of s_7, the sum can be carried out easily:

$$\sum_{s_7}|M_{71}|^2\lambda^{7}_{s_0s_1\ldots s_7}\lambda^{1}_{s_0s_1} = \sum_{s_7}\langle y^{7}_{s_0s_1s_2\ldots s_7}|y^{1}_{s_0s_1}\rangle\langle y^{1}_{s_0s_1}|y^{7}_{s_0s_1s_2\ldots s_7}\rangle\lambda^{7}_{s_0s_1s_2\ldots s_7}\lambda^{1}_{s_0s_1} = \lambda^{1}_{s_0s_1}\,. \tag{75}$$

Following the same reasoning, we can carry out the remainder of the sum using condition (43). We obtain

$$\sum_s |F_1|^2 = D^5\,. \tag{76}$$

The same technique applies to a general permutation. In general, each red or blue segment contributes one factor of D in the sum. Each segment with total length l_α (defined by the number of *links* in the segment) contains a sum over $l_\alpha + 1$ sites that contribute D, which means it leads to a suppression by D^{-l_α} compared with the trivial permutation case $\sum_s \prod_{i=1}^k \lambda^i_{s_0 s_1 \ldots s_i} = D^k$. Consequently,

$$\sum_s |F_1|^2 = D^{k-l_r}, \qquad \sum_s |F_2|^2 = D^{k-l_b} \tag{77}$$

with l_r and l_b the total length of the red and blue segments, respectively. Interestingly, the result only depends on the net length of blue and red segments l_b, l_r and is *independent* of the number of segments. Therefore, using Supplementary Equations (73) and (69) we obtain

$$\sum_s \left|\mathrm{tr}\left(B\tau^{-1}\right)\right| \le \frac{1}{2} D^k \left(D^{-l_b} + D^{-l_r}\right) \le D^{k-\lfloor \frac{L_\tau}{2} \rfloor}. \tag{78}$$

Performing the sum over τ we find

$$T \equiv \frac{1}{D^k} \sum_{\tau \neq \mathbb{1}} \sum_s \left|\mathrm{tr}\left(B\tau^{-1}\right)\right| \le \frac{1}{D^k} \sum_{\tau \neq \mathbb{1}} D^{k-\lfloor \frac{L_\tau}{2} \rfloor} = \frac{1}{D^k} \sum_{L=2}^{k} N(k,L) D^{k-\lfloor \frac{L}{2} \rfloor} \tag{79}$$

with $N(k,L)$ the number of permutation elements with $L_\tau = L$. It is easy to see that

$$N(k,L) \le \begin{pmatrix} k \\ L \end{pmatrix} L! = \frac{k!}{(k-L)!} < k^L \tag{80}$$

and thus

$$T < \sum_{L=2}^{\infty} k^L D^{-\lfloor \frac{L}{2} \rfloor} = \frac{(1+k)\frac{k^2}{D}}{1-\frac{k^2}{D}} = \frac{k^3}{D} + \frac{k^2}{D} + \mathscr{O}\left(\frac{k^5}{D^2}\right). \tag{81}$$

This proves Supplementary Equation (63). (Note that in the regime we are considering, $\frac{k^3}{D} < O\left(D^{-1/7}\right)$ is exponentially small.)

This completes the proof of Lemma 3.

3.3.3 The general incoherent access QUALM

To complete the proof of Theorem 4, we show that a general incoherent access QUALM cannot do better than a simple measurement QUALM. The key idea is the following. Since in an incoherent access QUALM **L** and **W** are only coupled by an LOCC, the output probability distribution of **W** can be expressed as a sequence of conditional probabilities, each given by an LOCC. Then we can organize this chain of conditional probabilities into two groups: those that depend on the lab oracle, and those that do not. The latter can be expressed as a probabilistic average over a set of deterministic functions (i.e. with conditional probability that is 1 on a particular output and 0 everywhere else), and for each deterministic function the QUALM reduces to a simple measurement QUALM.

More precisely, this is expressed in the following claim:

Claim 2 (*Derandomization claim*). For any incoherent access QUALM denoted by QUALM, there is a set of simple measurement QUALMs, $\{\mathsf{QUALM}_r\}_r$, and a fixed probability distribution over the indices r of $\Pi(r)$ (which crucially is independent of the lab oracle) such that

- The QUALM query complexity of each QUALM_r is the same as that of QUALM;
- For any lab oracle LO, the output of QUALM applied to LO is an average over the output of each QUALM_r applied to LO, where the average is taken with respect to the distribution $\Pi(r)$.

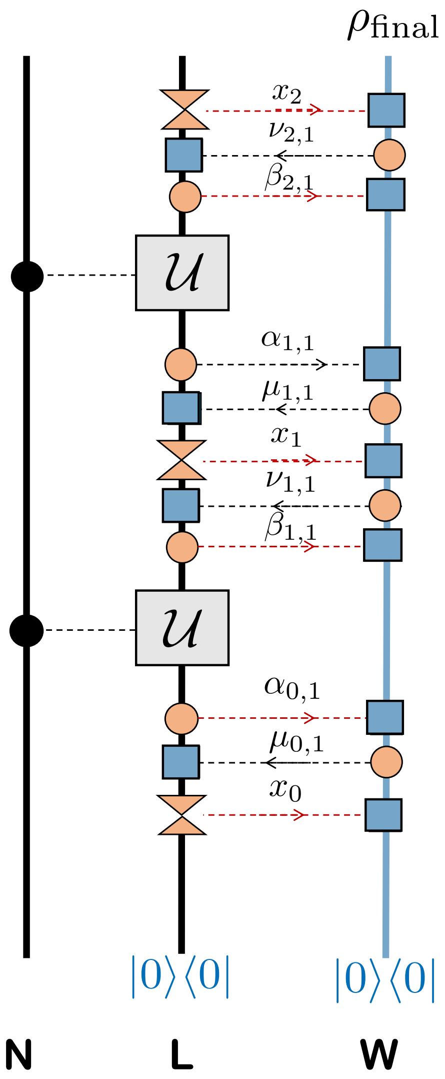

Supplementary Figure 6. *Illustration of the incoherent access QUALM*. This is a diagrammatic depiction of Definition 15. Each orange solid circle is a CP map and each blue box is a CPTP map. At least one of the CP maps between each application of the lab oracle is a complete measurement, indicated by the double triangle. The direction of the arrow in each horizontal dashed line indicates the direction of classical information flow. Only the LOCC's corresponding to $\beta_{i,j}$ and x_i lead to conditional probabilities that depend on the lab oracle, which are indicated by red dashed lines.

From this claim, it follows that if QUALM achieves the task of distinguishing the two lab oracles LOP_ℓ and LOQ_ℓ with error $< \frac{1}{3}$ (which means that the output probability distributions for the two lab oracles are at least $\frac{1}{3}$ apart in total variation distance; see Definition 16) then there must be a simple measurement QUALM of the same query complexity which achieves the same total variation distance between the two output distributions. By Lemma 3, this is impossible if the query complexity k of QUALM is smaller than order $2^{2\ell/7}$, because in this case the two output distributions of the simple measurement QUALM, for the two lab oracles, are exponentially close in ℓ.

We would like to emphasize that Claim 2 applies to all incoherent access QUALMs with arbitrary lab oracles. To prove Theorem 4 we only need to apply it to the specific lab oracles LOP_ℓ and LOQ_ℓ, but later in Supplementary Note 5 we will apply it to other cases.

It thus remains to prove Claim 2 to finish the proof of Theorem 4.

Towards this goal, we introduce some notation. An incoherent access QUALM is illustrated in Supplementary Figure 6. We have labeled the complete measurement results in the ith round by bit strings x_i. All other one-round LOCC's are labeled by Greek letters. The $\mathsf{L} \to \mathsf{W}$ LOCC's after a complete measurement x_i and before the lab oracle are labeled by $\alpha_{i,j}$'s, and those after the lab oracle and before the next complete measurement are labeled by $\beta_{i,j}$'s. Likewise, the $\mathsf{W} \to \mathsf{L}$ LOCC's after the complete measurement x_i and before the lab oracle are labeled by $\mu_{i,j}$'s, and those after the lab oracle and before the next complete measurement are labeled by $\nu_{i,j}$'s. Note that $\alpha_{i,j}$ and $\beta_{i,j}$ are weak measurement results (i.e., results of POVM measurements) of L that are sent to W, and $\mu_{i,j}$ and $\nu_{i,j}$ are weak measurement results of W that are sent to L.

Proof of Claim 2. We first observe that the output density matrix of the W register, when the QUALM is applied on some lab oracle, is fully determined (i.e., specified *deterministically*) by the values that the random variables $\alpha_{i,j}, \beta_{i,j}, \mu_{i,j}, \nu_{i,j}, x_i$ take. Namely, the only dependence on the lab oracle is through these values. We denote the final reduced density matrix on S_{out} as a function of these variables by $\rho^{\mathsf{S}_{\text{out}}}(\alpha, \beta, \mu, \nu, x)$.

Let the probability distribution over these variables be $\Pi(\alpha, \beta, \mu, \nu, x)$. The output density matrix is the average

$$\rho_{\text{final}} = \sum_{\alpha,\beta,\mu,\nu,x} \Pi(\alpha,\beta,\mu,\nu,x)\rho^{\mathsf{S}_{\text{out}}}(\alpha,\beta,\mu,\nu,x)\,. \tag{82}$$

Crucially, the state $\rho^{\mathsf{S}_{\text{out}}}(\alpha,\beta,\mu,\nu,x)$ does not depend on the lab oracle since W can only probe L using the measurement results α,β,μ,ν,x, but the probability distribution $\Pi(\alpha,\beta,\mu,\nu,x)$ does depend on the lab oracle.

For the two lab oracles we are interested in, we denote the corresponding probability distributions by Π^P and Π^Q. We have

$$\begin{aligned}\left\|\rho^P_{\text{final}} - \rho^Q_{\text{final}}\right\|_1 &\leq \sum_{\alpha,\beta,\mu,\nu,x} \left|\Pi^P(\alpha,\beta,\mu,\nu,x) - \Pi^Q(\alpha,\beta,\mu,\nu,x)\right| \left\|\rho^{\mathsf{S}_{\text{out}}}(\alpha,\beta,\mu,\nu,x)\right\|_1 \\ &= \sum_{\alpha,\beta,\mu,\nu,x} \left|\Pi^P(\alpha,\beta,\mu,\nu,x) - \Pi^Q(\alpha,\beta,\mu,\nu,x)\right|\,. \end{aligned} \tag{83}$$

Therefore it is sufficient to show that the probability distributions $\Pi^P(\alpha,\beta,\mu,\nu,x), \Pi^Q(\alpha,\beta,\mu,\nu,x)$ are close to each other in total variation distance. In the following, we will omit the lab oracle label P,Q since the discussion is independent of which of the two lab oracles is being used. We will return to the comparison of the particular lab oracles at the end.

In its current form, $\Pi(\alpha,\beta,\mu,\nu,x)$ is not the output of a simple measurement QUALM. There are two key differences between the general case and simple measurement case: (i) In a simple measurement QUALM there is only a single (generalized) projective measurement between two applications of lab oracles, while in the general case L is measured by many weak measurements in addition to projective measurements. (ii) In between the measurements with outputs α and β, there are quantum channels applied to L controlled by μ,ν, but μ,ν are random variables determined by weak measurements on W, and therefore depend probabilistically on all previous parameters. By comparison, in a simple measurement QUALM, the previous parameters $s_0 s_1 \ldots s_{i-1}$ have a *deterministic* effect on the measurement basis vector $\left|y^i_{s_0 s_1 \ldots s_i}\right\rangle$, the weights $\lambda^i_{s_0 s_1 \ldots s_i}$, and the prepared state $\sigma^i_{s_0 s_1 \ldots s_i}$. In the following we will carry some further transformations to relate the probability distribution $\Pi(\alpha,\beta,\mu,\nu,x)$ to simple measurement QUALMs. The proof contains three steps.

1. Decomposition of the chain of conditional probabilities. Each one-round LOCC channel in Definition 14 has the form $\mathscr{E}_m = \sum_\eta \mathscr{E}_{m\eta}$, with η labeling α, β, μ, ν or x for different one-round LOCC's. The $\mathscr{E}_{m\eta}$ are CP maps of the form $\mathscr{M}^{\mathbf{L}}_{m\eta} \otimes \mathscr{N}^{\mathbf{W}}_{m\eta}$ (for an $\mathbf{L}$-to-$\mathbf{W}$ LOCC) or $\mathscr{N}^{\mathbf{L}}_{m\eta} \otimes \mathscr{M}^{\mathbf{W}}_{m\eta}$ (for a $\mathbf{W}$-to-$\mathbf{L}$ LOCC). We have $m = 1, 2, ..., k \cdot r \equiv M$ if there are k rounds of application of lab oracles, and r rounds of LOCC's in each step. By a slight abuse of notation, here we denote by $\mathscr{E}_{M\eta_M}$ the last channel applied in the QUALM *followed by* a channel which traces out all qubits in $\mathbf{W}$ which are not in $\mathbf{S}_{\text{out}}$. This means that the final density matrix is a state of $\mathbf{S}_{\text{out}}$. We use the general notation $\mathscr{E}_{m\eta}$ for convenience in the following discussion about conditional probabilities. We will return to the more specific labels $\alpha, \beta, \mu, \nu, x$ after this discussion.

The effect of applying $\mathscr{E}_m,\ m = 1, 2, ..., M$ sequentially to an initial state state ρ_{in} is

$$\begin{aligned}\rho_{\text{final}} &= \sum_{\eta_1 \eta_2 \cdots \eta_M} \mathscr{E}_{M\eta_M} \circ \mathscr{E}_{M-1,\eta_{M-1}} \cdots \circ \mathscr{E}_{1\eta_1}[\rho_{\text{in}}] \\ &= \sum_{\eta_1 \eta_2 \cdots \eta_M} \Pi_M(\eta_1, \eta_2, ..., \eta_M)\, \rho_M(\eta_1, \eta_2, ..., \eta_M) \end{aligned} \tag{84}$$

with

$$\Pi_M(\eta_1, \eta_2, ..., \eta_M) = \text{tr}\left(\mathscr{E}_{M\eta_M} \circ \mathscr{E}_{M-1,\eta_{M-1}} \cdots \circ \mathscr{E}_{1\eta_1}[\rho_{\text{in}}]\right) \tag{85}$$

$$\rho_M(\eta_1, \eta_2, ..., \eta_M) = \frac{\mathscr{E}_{M\eta_M} \circ \mathscr{E}_{M-1,\eta_{M-1}} \cdots \circ \mathscr{E}_{1\eta_1}[\rho_{\text{in}}]}{\Pi_M(\eta_1, \eta_2, ..., \eta_m)}\,. \tag{86}$$

Normalization of the channel $\sum_{\eta_M} \mathscr{E}_{M\eta_M}$ implies that

$$\sum_{\eta_M} \Pi_M(\eta_1, \eta_2, ..., \eta_M) = \Pi_{M-1}(\eta_1, \eta_2, ..., \eta_{M-1}) \tag{87}$$

where the right-hand side is the probability obtained by only applying the first $M-1$ CP maps. Thus the probability Π_M can be written as a chain of conditional probabilities:

$$\Pi_M(\eta_1, \eta_2, ..., \eta_M) = \prod_{m=2}^{M} \Pi_m(\eta_m | \eta_1, \eta_2, ..., \eta_{m-1})\, \Pi_1(\eta_1) \tag{88}$$

$$\Pi_m(\eta_m | \eta_1, \eta_2, ..., \eta_{m-1}) = \frac{\Pi_m(\eta_1, \eta_2, ..., \eta_m)}{\Pi_{m-1}(\eta_1, \eta_2, ..., \eta_{m-1})} = \text{tr}\left\{\mathscr{E}_{m\eta_m}[\rho_{m-1}(\eta_1, \eta_2, ..., \eta_{m-1})]\right\}. \tag{89}$$

The conditional probability is determined by a weak measurement on the state ρ_{m-1} which depends on the previous measurement outputs $\eta_1, ..., \eta_{m-1}$.

Now we apply this general decomposition to an incoherent access QUALM. We decompose the probability distribution $\Pi(\alpha, \beta, \mu, \nu, x)$ into a product of two terms

$$\Pi(\alpha, \beta, \mu, \nu, x) = \Pi_1(\beta, x | \alpha, \mu, \nu)\, \Pi_2(\alpha, \mu, \nu | \beta, x) \tag{90}$$

where we denote

$$\Pi_1(\beta, x | \alpha, \mu, \nu) \equiv \prod_{i,j} \Pi(\beta_{i,j} | ...)\, \Pi(x_i | ...) \tag{91}$$

$$\Pi_2(\alpha, \mu, \nu | \beta, x) \equiv \prod_{i,j} \Pi(\mu_{i,j} | ...)\, \Pi(\nu_{i,j} | ...)\, \Pi(\alpha_{i,j} | ...)\,. \tag{92}$$

Π_1 consists of the product of terms in Π in which β or x are random variables conditioned on others, whereas Π_2 is the product of those terms in which the α, μ, ν are random. Note that the notation used for the terms in the right-hand side means that the conditioning is done on all *earlier parameters*. Here and below, in conditional probabilities we will denote all previous measurement outputs $\alpha, \beta, \mu, \nu, x$ by ellipses, i.e. "...". Since all labels have a unique time ordering, this abbreviation does not cause any ambiguity. We now claim:

Claim 3. For all values of β, x, the conditional probability distributions $\Pi_2(\alpha,\mu,\nu|\beta,x)$ do not depend on the lab oracle.

Proof. In this case, for fixed labels α,β,μ,ν,x, the conditional state (corresponding to $\rho_m(\eta_1,\eta_2,...,\eta_m)$) in the general discussion is a tensor product state of $\mathbf{L}$ and $\mathbf{W}$. We do not see a way to avoid having tedious notation, so to avoid confusion let us first consider the example case in Supplementary Figure 6(a). The state after applying the LOCC labeled by $\nu_{1,1}$ is

$$\rho(\nu_{1,1},\beta_{1,1},\alpha_{0,1},\mu_{0,1},x_0) = \rho^{\mathbf{L}}(\nu_{1,1},...)\otimes\rho^{\mathbf{W}}(\nu_{1,1},...) \tag{93}$$

$$\rho^{\mathbf{L}}(\nu_{1,1},...) = \Omega_{\mathbf{L}}^{-1}\mathcal{N}^{\mathbf{L}}_{\nu_{1,1}}\circ\mathcal{M}^{\mathbf{L}}_{\beta_{1,1}}\circ\mathcal{U}\circ\mathcal{M}^{\mathbf{L}}_{\alpha_{0,1}}\circ\mathcal{N}^{\mathbf{L}}_{\mu_{0,1}}[|x_0\rangle\langle x_0|] \tag{94}$$

$$\rho^{\mathbf{W}}(\nu_{1,1},...) = \Omega_{\mathbf{H}}^{-1}\mathcal{M}^{\mathbf{W}}_{\nu_{1,1}}\circ\mathcal{N}^{\mathbf{W}}_{\beta_{1,1}}\circ\mathcal{N}^{\mathbf{W}}_{\alpha_{0,1}}\circ\mathcal{M}^{\mathbf{W}}_{\mu_{0,1}}\circ\mathcal{N}^{\mathbf{W}}_{x_0}[|0_{\mathbf{W}}\rangle\langle 0_{\mathbf{W}}|]\,. \tag{95}$$

As a reminder, the $\mathcal{M}$'s are CP maps and the $\mathcal{N}$'s are CPTP maps; see Definition 14. $\mathcal{U}$ denotes the superoperator associated with applying the unitary U from the lab oracle, and $\Omega_{\mathbf{L}}^{-1},\Omega_{\mathbf{H}}^{-1}$ are normalization factors. Because $\mathcal{N}^{\mathbf{L}}_{\nu_{1,1}}$ is trace-preserving, the conditional probability $\Pi(\nu_{1,1}|\beta_{1,1},...,x_0)$ defined in Supplementary Equation (89) only depends on $\rho^{\mathbf{W}}$:

$$\Pi(\nu_{1,1}|\beta_{1,1},...,x_0) = \mathrm{tr}\left\{\mathcal{M}^{\mathbf{W}}_{\nu_{1,1}}\left(\rho^{\mathbf{W}}(\beta_{1,1},\alpha_{0,1},\mu_{0,1},x_0)\right)\right\} \tag{96}$$

where $\rho^{\mathbf{W}}(\beta_{1,1},\alpha_{0,1},\mu_{0,1},x_0)$ is the state before applying the CP map $\mathcal{M}^{\mathbf{W}}_{\nu_{1,1}}$. Explicitly, we have $\rho^{\mathbf{W}}(\beta_{1,1},\alpha_{0,1},\mu_{0,1},x_0) = \tilde{\Omega}_{\mathbf{W}}^{-1}\mathcal{N}^{\mathbf{W}}_{\beta_{1,1}}\circ\mathcal{N}^{\mathbf{W}}_{\alpha_{0,1}}\circ\mathcal{M}^{\mathbf{W}}_{\mu_{0,1}}\circ\mathcal{N}^{\mathbf{W}}_{x_0}[|0_{\mathbf{W}}\rangle\langle 0_{\mathbf{W}}|]$.

Importantly, $\rho^{\mathbf{W}}(\beta_{1,1},...,x_0)$ only depends on the labels and does not depend on the lab oracle. Therefore the same is true for the conditional probability $\Pi(\nu_{1,1}|\beta_{1,1},...,x_0)$. This argument applies to all conditional probabilities $\Pi(\nu_{i,j}|...)$, $\Pi(\mu_{i,j}|...)$ corresponding to $\mathbf{W}$-to-$\mathbf{L}$ LOCC's.

Following a similar argument, one can see that the conditional probabilities corresponding to $\mathbf{L}$-to-$\mathbf{W}$ LOCC's are determined by the conditional state of $\mathbf{L}$, which does depend on the lab oracle, denoted by $\mathcal{U}$ in Supplementary Equation (94). For example,

$$\Pi(\beta_{1,1}|\alpha_{0,1},\mu_{0,1},x_0) = \mathrm{tr}\left\{\mathcal{M}^{\mathbf{L}}_{\beta_{1,1}}\left[\rho^{L}(\alpha_{0,1},\mu_{0,1},x_0)\right]\right\} \tag{97}$$

Up to this point, the discussion applies as long as $\mathbf{L}$ and $\mathbf{W}$ are only coupled by LOCC's, and we have not used the condition that there is a complete projective measurement between any two subsequent applications of the lab oracle. The projective measurement leads to an important simplification because the state after the measurement $|x_i\rangle$ is completely determined, such that the application of the lab oracle before this step has no effect on the conditional states $\rho^{\mathbf{L}}$ occuring afterwards. Consequently, the conditional probability $\Pi(\beta_{i,j}|...)$ only depends on the ith application of the lab oracle, and $\Pi(\alpha_{i,j}|...)$ does not depend on the lab oracle at all. □

2. Derandomization of the lab oracle independent part. Since Π_2 in Supplementary Equation (92) is independent of the lab oracle, we can consider it as probabilistic instructions given by $\mathbf{W}$ to $\mathbf{L}$. If the probability distribution Π_2 did not depend on β, x, Supplementary Equation (90) would mean that the general QUALM output is a probabilistic average for fixed α,μ,ν, and therefore we can consider the QUALM as an average over QUALMs with fixed α,μ,ν, treating these variables as constants, and we can focus on Π_1. However, since the conditional probability distribution in each term may depend on the previous measurement results β, x, which in turn depend on the lab oracle, we are not ready to drop Π_2. Instead, we introduce an additional step to relate these probabilistic instructions to deterministic ones.

For this purpose, we consider the following simple mathematical fact:

Fact 2. For an arbitrary probability distribution $P(\alpha)$ of a discrete index $\alpha = 1,2,...,N$ (normalized as $\sum_{\alpha=1}^{N}P(\alpha)=1$), there exists a function $f(r):[0,1]\to\{1,2,...,N\}$ which takes a variable $r\in[0,1]$ and maps it to one of the integers $\alpha = 1,2,...,N$, such that when r is a random variable with uniform probability distribution, the probability that $f(r)$ takes value α is $P(\alpha)$:

$$\mathrm{Prob}(f(r)=\alpha) = \int_0^1 dr\,\delta_{\alpha,f(r)} = P(\alpha)\,, \tag{98}$$

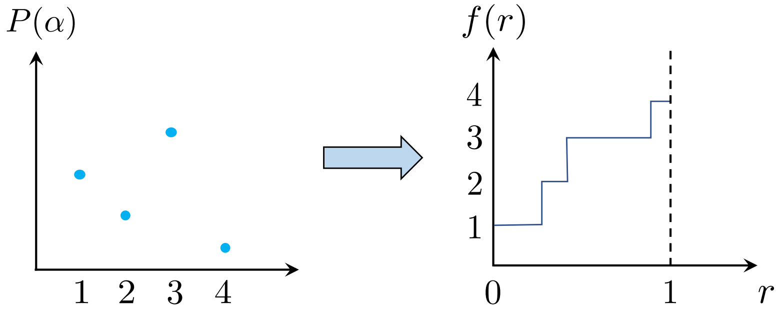

Supplementary Figure 7. *Illustration of the function $f(r)$.* Here we show the graph of $f(r)$, defined in Supplementary Equation (99), such that the probability of $f(r) = \alpha$ is $P(\alpha)$. In the example, $\alpha = 1,2,3,4$.

where $\delta_{i,j}$ is the Kronecker delta.

Intuitively, one just needs to divide the interval $[0,1]$ into N subintervals, and make sure that the αth interval has width P_α so that the probability with which r falls in that interval is α. An explicit formula for $f(r)$ is

$$f(r) = \sum_{\alpha=1}^{N} \alpha\, \theta\left(r - \sum_{\beta=1}^{\alpha-1} P(\beta)\right) \theta\left(\sum_{\beta=1}^{\alpha} P(\beta) - r\right) \tag{99}$$

where $\theta(x)$ is the step function that is 1 for $x > 0$ and 0 otherwise. This function has N steps, taking value α when $\sum_{\beta=1}^{\alpha-1} P(\beta) < r < \sum_{\beta=1}^{\alpha} P(\beta)$. An example is illustrated in Supplementary Figure 7.

Now for each conditional probability in Π_2 in Supplementary Equation (92) we introduce an independent random variable. For example, $\Pi(\mu_{i,j}|\ldots)$ corresponds to a function $f_\mu\left(r^\mu_{ij};\ldots\right)$. For each fixed value r^μ_{ij}, this function outputs a particular index $\mu_{i,j}$ which depends on the previous measurement results. The same decomposition is incorporated in the $\nu_{i,j}$ and $\alpha_{i,j}$ terms with independent random variables r^ν_{ij} and r^α_{ij}. Applying the decomposition in Supplementary Equation (98) to all these variables we obtain

$$\Pi_2(\alpha,\mu,\nu|\beta,x) = \int_0^1 \prod_{ij} dr^\mu_{ij}\, dr^\nu_{ij}\, dr^\alpha_{ij}\, \delta_r(\alpha,\mu,\nu|\beta,x) \tag{100}$$

$$\delta_r(\alpha,\mu,\nu|\beta,x) = \prod_{ij} \delta_{\mu_{i,j}, f^\mu_{ij}(r^\mu_{ij};\ldots)}\, \delta_{\nu_{i,j}, f^\nu_{ij}(r^\nu_{ij};\ldots)}\, \delta_{\alpha_{i,j}, f^\alpha_{ij}(r^\alpha_{ij};\ldots)}\,. \tag{101}$$

This expression means that we can decompose the probability Π_2 into an average of δ-functions $\delta_r(\alpha,\mu,\nu|\beta,x)$ over uniform, independent random variables $r^{\alpha,\mu,\nu}_{ij}$. In more physical terms, if we replace Π_2 by δ_r in the QUALM, it corresponds to replacing the probabilistic instructions by deterministic ones: at each step, **W** performs a computation and obtains a particular value of μ,ν or α based on the previous measurement results. Applying this decomposition in Supplementary Equation (90) we obtain

$$\Pi(\alpha,\beta,\mu,\nu,x) = \int dr\, \Pi_1(\beta,x|\alpha,\mu,\nu)\, \delta_r(\alpha,\mu,\nu|\beta,x) \tag{102}$$

where we have used $\int dr$ as an abbreviation for the integration over all variables $r^{\mu,\nu,\alpha}_{ij}$. Since Π_2 and therefore the functions δ_r are independent of the lab oracle, for two different lab oracles the 1-norm difference defined in the right-hand side of Supplementary Equation (83) is bounded by

$$\sum_{\alpha,\beta,\mu,\nu,x} \left|\Pi^P(\alpha,\beta,\mu,\nu,x) - \Pi^Q(\alpha,\beta,\mu,\nu,x)\right| \le \int dr\, \Delta^{PQ}_r \tag{103}$$

where

$$\Delta_r^{PQ} = \sum_{\alpha,\beta,\mu,\nu,x} \left| \Pi_1^P(\beta,x|\alpha,\mu,\nu) - \Pi_1^Q(\beta,x|\alpha,\mu,\nu) \right| \delta_r(\alpha,\mu,\nu|\beta,x)$$
$$\equiv \sum_{\beta,x} \left| \Pi_r^P(\beta,x) - \Pi_r^Q(\beta,x) \right| \tag{104}$$

and

$$\Pi_r(\beta,x) = \sum_{\alpha,\mu,\nu} \Pi_1(\beta,x|\alpha,\mu,\nu)\delta_r(\alpha,\mu,\nu|\beta,x). \tag{105}$$

The reason for the last equality in Supplementary Equation (104) is that the sum in Supplementary Equation (105) contains only a single non-zero term, due to the Kronecker delta function.

Observe that $\Pi_r(\beta,x)$ is the output probability distribution of a different QUALM, where all indices α,μ,ν are deterministic functions specified by $\delta_r(\alpha,\mu,\nu|\beta,x)$ as functions of previous measurement results. This QUALM is derived from the original QUALM by fixing the input random bits to be r. Our remaining task is to show the relation of this QUALM to a simple measurement QUALM. In particular, we will prove that Δ_r^{PQ} is upper bounded by the total variation distance between the two lab oracles, achieved by a simple measurement QUALM.

3. Relating the lab oracle dependent part to a simple measurement QUALM. We can decompose the conditional probability Π_1 in Supplementary Equation (91) into the terms after the ith application of the lab oracle:

$$\Pi_1(\beta,x|\alpha,\mu,\nu) = \prod_{i=1}^{k} \Pi_i(\beta_{i,j},x_i|\ldots) \tag{106}$$

where

$$\Pi_i(\beta_{i,j},x_i|\ldots) = \prod_j \Pi(\beta_{i,j}|\ldots)\,\Pi(x_i|\ldots)$$
$$= \langle x_i | \mathscr{N}^{\mathbf{L}}_{\nu_{i,j\max}} \circ \mathscr{M}^{\mathbf{L}}_{\beta_{i,j\max}} \circ \cdots \circ \mathscr{M}^{\mathbf{L}}_{\beta_{i,1}} \circ \mathscr{U}[\rho^{\mathbf{L}}_{i-1}] | x_i \rangle \tag{107}$$

and $\rho^{\mathbf{L}}_{i-1}$ the state of $\mathbf{L}$ right before the ith application of the lab oracle, which depends on the previous measurement results. Here $|x_i\rangle$ denotes the vectors of the complete ith measurement.[6] Note that each $\nu_{i,j}$ already takes a deterministic value that is determined by previous measurement results. The difference with the simple measurement QUALM case is that here we have a sequence of weak measurements defined by the CP maps

$$\mathscr{E}^i_{\beta_{i,1}\beta_{i,2}\cdots\beta_{i,j\max}} = \mathscr{N}^{\mathbf{L}}_{\nu_{i,j\max}} \circ \mathscr{M}^{\mathbf{L}}_{\beta_{i,j\max}} \circ \cdots \circ \mathscr{M}^{\mathbf{L}}_{\beta_{i,1}} \tag{108}$$

before the projective measurement on states $|x_i\rangle$. To show that this is equivalent to the generalized projective measurement in a simple measurement QUALM, we first use the following fact:

Fact 3. Consider a family of CP maps $\mathscr{E}_m$, $m = 1,2,\ldots,M$ from states in a Hilbert space $\mathbf{L}$ to operators in the same Hilbert space, such that $\sum_{m=1}^{M} \mathscr{E}_m$ is a CPTP map. In addition, introduce an auxiliary system $\mathbf{A}$ with Hilbert space dimension M, and an orthonormal basis $|m_{\mathbf{A}}\rangle$, $m = 1,2,\ldots,M$. Then the map $\mathscr{E}$ defined in the following is a CPTP map from $\mathbf{L}$ to $\mathbf{L}\otimes\mathbf{A}$:

$$\mathscr{E}[\rho] = \sum_{m=1}^{M} \mathscr{E}_m[\rho] \otimes |m_{\mathbf{A}}\rangle\langle m_{\mathbf{A}}|\,. \tag{109}$$

[6]Though the notation is slightly confusing, note that these vectors form a general orthonormal basis which is not necessarily the computational basis.

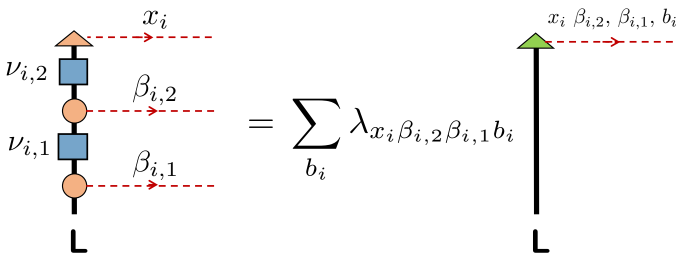

Supplementary Figure 8. *CP maps applied to the lab oracle and subsequent measurement.* We depict how the CP maps after the ith application of the lab oracle (see Supplementary Figure 6) followed by a projective measurement are equivalent to a single POVM with operators $\lambda_{x_i\beta_{ij}b_i} \left|y_{x_i\beta_{ij}b_i}\right\rangle \left\langle y_{x_i\beta_{ij}b_i}\right|$, with a sum over the extra index b_i. This is an application of the general result in Supplementary Equation (112).

This result can be verified by checking directly that $\mathscr{E}$ is completely positive and trace-preserving. Now one can see that applying $\mathscr{E}_m$ followed by a projection on $|x_i\rangle$ is equivalent to applying the CPTP map $\mathscr{E}$ followed by a projection on $|x_i\rangle \otimes |m_{\mathbf{A}}\rangle$:

$$\langle x_i| \mathscr{E}_m[\rho] |x_i\rangle = (\langle x_i| \otimes \langle m_{\mathbf{A}}|) \mathscr{E}[\rho] (|x_i\rangle \otimes |m_{\mathbf{A}}\rangle) . \tag{110}$$

To relate to the projective measurements, we can further decompose $\mathscr{E}$ into Kraus operators:

$$\mathscr{E}[\rho] = \sum_b A_b \rho A_b^\dagger \tag{111}$$

with $A_b : \mathbf{L} \to \mathbf{L} \otimes \mathbf{A}$ being linear operators. Using this decomposition we obtain

$$\langle x_i| \mathscr{E}_m(\rho) |x_i\rangle = \sum_b \left\langle y_{x_imb}\right| \rho \left|y_{x_imb}\right\rangle \lambda_{x_imb} \tag{112}$$

$$\lambda_{x_imb} = (\langle x_i| \otimes \langle m_{\mathbf{A}}|) A_b A_b^\dagger (|x_i\rangle \otimes |m_{\mathbf{A}}\rangle) \tag{113}$$

$$\left|y_{x_imb}\right\rangle = \lambda_{x_imb}^{-1/2} A_b^\dagger |x_i\rangle \otimes |m_{\mathbf{A}}\rangle . \tag{114}$$

One can check that

$$\sum_{x_imb} \lambda_{x_imb} \left|y_{x_imb}\right\rangle \left\langle y_{x_imb}\right| = \sum_b A_b^\dagger A_b = \mathbb{1} . \tag{115}$$

Therefore, the basis $\{\left|y_{x_imb}\right\rangle\}$ and coeffcients λ_{x_imb} satisfy the requirements of a simple measurement QUALM.

Now we can apply the procedure above for $\mathscr{E}^i_{\beta_{i,1}\ldots\beta_{i,j_{\max}}}$ defined in Supplementary Equation (108) for each round i (m now corresponds to all $\beta_{i,j}$ with fixed i). This defines states $\left|y_{x_i,\beta_{i,j},b_i}\right\rangle$ and weights $\lambda_{x_i,\beta_{i,j},b_i}$, as is illustrated in Supplementary Figure 8. We can then define a simple measurement QUALM which carries POVM operators $\lambda_{x_i,\beta_{i,j},b_i} \left|y_{x_i,\beta_{i,j},b_i}\right\rangle \left\langle y_{x_i,\beta_{i,j},b_i}\right|$, and prepares states $\rho_i^{\mathbf{L}}$ (defined in Supplementary Equation (107)). The output of this simple measurement QUALM is not identical to $\Pi_r(\beta,x)$ in Supplementary Equation (105) since it also depends on the new variables b_i. However, according to Supplementary Equation (112) they are related by simply performing the sum over b_i:

$$\Pi_r(\beta,x) = \sum_{b_1 b_2 \ldots b_k} \widetilde{\Pi}_r(\beta,x,b) , \tag{116}$$

where $\widetilde{\Pi}_r$ on the right-hand side is the probability distribution of the simple measurement QUALM defined above.

In other words, the probability distribution Π_r is obtained by applying the simple measurement QUALM and forgetting about some of the variables b, which will only reduce the capability of distinguishing two lab oracles. More explicitly, Δ_r^{PQ} in Supplementary Equation (104) is bounded by

$$\Delta_r^{PQ} \leq \sum_{\beta,x,b} \left| \widetilde{\Pi}_r^P(\beta,x,b) - \widetilde{\Pi}_r^Q(\beta,x,b) \right| . \tag{117}$$

The right-hand side of this equation is the bias for the simple measurement QUALM, so that we can apply the upper bound given by Lemma 3. Using this bound and Supplementary Equations (83) and (103), we conclude that a general incoherent access QUALM with k rounds of applications of the lab oracle ($k < \left(2^\ell/\sqrt{6}\right)^{4/7}$) and ℓ qubits in L has a bias upper bound

$$\left\| \rho_{\text{final}}^P - \rho_{\text{final}}^Q \right\|_1 \leq \mathscr{O}\left(\frac{k^3}{2^\ell}\right) . \tag{118}$$

This proves Theorem 4. □

Supplementary Note 4: Corollaries

Here we discuss three interesting corollaries of Theorem 4, corresponding to different kinds of tomography-themed quantum tasks and QUALMs. These are summarized informally below:

- In the first corollary, we extend Theorem 4 to the task of distinguishing between lab oracles encoding more elaborate ensembles of unitaries.
- We provide a similar result for the task of distinguishing lab oracles that produce certain ensembles of states.
- Finally, we provide a related bound for distinguishing a random unitary channel from a completely depolarizing channel.

We now proceed with a more detailed discussion.

Supplementary Note 4.1: Correlated random unitaries

Definition 21 (*A unitary ensemble distinction problem*). Consider a lab oracle LO_ℓ^μ such that its quantum channel applies a unitary U_k to L upon being queried k times. That is, the lab oracle superoperator applies U_1 the first time it is queried, U_2 the second time it is queried, and so on. Suppose that the lab oracle superoperator determines each of these unitaries by sampling from a probability distribution $\mu(U_1, U_2, ..., U_k, ...)$, and that the probability distribution is left-invariant:

$$\mu(VU_1, VU_2, ..., VU_k, ...) = \mu(U_1, U_2, ..., U_k, ...) \tag{119}$$

for an arbitrary unitary transformation V. The task we consider is to distinguish LO_ℓ^μ and $\mathsf{LO}_\ell^{\mu'}$ for different distributions μ and μ'.

Note that LOP_ℓ and LOQ_ℓ discussed above are examples of lab oracles of the form LO_ℓ^μ. In particular, LOP_ℓ corresponds to the U_i chosen i.i.d., and LOQ_ℓ corresponds to all U_i's perfectly correlated with each other. Note also that to implement the lab oracle described above in our framework, we need a register within N which is incremented by 1 every time the lab oracle is called. We will not formally write down the lab oracle here since this is straightfoward, and its exact form is not important for our discussion.

We have the following corollary of Theorem 4:

Corollary 3. For an arbitrary left-invariant probability distribution $\mu(U_1, U_2, ..., U_k, ...)$ and an arbitrary incoherent access QUALM which queries the lab oracle at most $k < \left(2^\ell/\sqrt{6}\right)^{4/7}$ times, the output density matrix $\rho_{\mathsf{S}_{\text{out}}}^\mu$ of the QUALM

when the lab oracle is LO_ℓ^μ and the output density matrix $\rho^P_{\mathbf{S}_{\rm out}}$ of the QUALM when the lab oracle is LOP_ℓ satisfy the following bound:

$$\left\|\rho^\mu_{\mathbf{S}_{\rm out}} - \rho^P_{\mathbf{S}_{\rm out}}\right\|_1 \leq \mathcal{O}\left(\frac{k^3}{2^\ell}\right). \tag{120}$$

Proof. To prove this corollary, we define $U_i = U_1 R_i$ with $R_1 = \mathbb{1}$. Due to left-invariance, $\mu\left(U_1, U_2, ..., U_k, ...\right) = \mu\left(\mathbb{1}, R_2, R_3, ..., R_k, ...\right)$. For a given incoherent access QUALM, the output density operator can be expressed as an average

$$\rho^\mu_{\mathbf{S}_{\rm out}} = \int \prod_{i\geq 2}^k dR_i\, \mu\left(\mathbb{1}, R_2, R_3, ..., R_k\right) \int dU_1\, \rho_{\mathbf{S}_{\rm out}}(U_1, R_2, R_3, ..., R_k) \tag{121}$$

where $\rho_{\mathbf{S}_{\rm out}}(U_1, R_2, R_3, ..., R_k)$ is the output state of $\mathbf{S}_{\rm out}$ for fixed $U_1, R_2, R_3, ..., R_k$. Now if we carry out the integration over U_1, we see that

$$\tilde{\rho}_{\mathbf{S}_{\rm out}}(R_2, R_3, ..., R_k) \equiv \int dU_1\, \rho_{\mathbf{S}_{\rm out}}(U_1, R_2, ..., R_k) \tag{122}$$

can be interpreted as the output state of a modified QUALM with fixed unitary lab oracle LOQ_ℓ. In this modified QUALM, there is an additional gate R_i before each lab oracle. The new QUALM is still incoherent, so that Theorem 4 implies

$$\left\|\tilde{\rho}_{\mathbf{S}_{\rm out}}(R_2, R_3, ..., R_k) - \rho^P_{\mathbf{S}_{\rm out}}\right\|_1 \leq \mathcal{O}\left(\frac{k^3}{2^\ell}\right). \tag{123}$$

Integration over R_i in Supplementary Equation (121) gives us

$$\left\|\rho^\mu_{\mathbf{S}_{\rm out}} - \rho^P_{\mathbf{S}_{\rm out}}\right\|_1 \leq \int \prod_{i=2}^k dR_i \mu\left(\mathbb{1}, R_2, R_3, ..., R_k\right) \left\|\tilde{\rho}_{\mathbf{S}_{\rm out}}(R_2, R_3, ..., R_k) - \rho^P_{\mathbf{S}_{\rm out}}\right\|_1 \leq \mathcal{O}\left(\frac{k^3}{2^\ell}\right). \tag{124}$$

□

This result guarantees (following the same argument as in the proof of Theorem 4; see the end of Supplementary Note 3.2) that the lab oracles LO_ℓ^μ, $\mathsf{LO}_\ell^{\mu'}$ corresponding to two different left-invariant probability distributions μ and μ' are difficult to distinguish since they are both "close" to LOP_ℓ.

Supplementary Note 4.2: State ensemble distinction

Definition 22 (*A state ensemble distinction problem*). Suppose we have a lab oracle LOS_ℓ^ν where its quantum channel prepares the state $|\psi_k\rangle$ on $\mathbf{L}$ upon being queried k times. Specifically, the lab oracle superoperator prepares $|\psi_1\rangle$ the first time it is queried, prepares $|\psi_2\rangle$ the second time it is queried, etc. We suppose that the lab oracle superoperator determines these states by sampling from a probability distribution $\nu\left(|\psi_1\rangle, |\psi_2\rangle, ..., |\psi_k\rangle, ...\right)$, and require that the probability distribution is unitarily invariant:

$$\nu\left(U|\psi_1\rangle, U|\psi_2\rangle, ..., U|\psi_k\rangle, ...\right) = \nu\left(|\psi_1\rangle, |\psi_2\rangle, ..., |\psi_k\rangle, ...\right) \tag{125}$$

for any unitary U. We consider the task of distinguishing LOS_ℓ^ν and $\mathsf{LOS}_\ell^{\nu'}$ where ν and ν' are distinct distributions.

An example is distinguishing between two lab oracles where one always generates the same Haar random state, and the other generates a new Haar random state each time. This is analogous to distinguishing LOQ_ℓ and LOP_ℓ in the setting of unitary ensembles. Another example to consider is that one lab oracle always generates the same Haar random state while the other one randomly generates one of the two independently chosen Haar random states $|\psi_1\rangle, |\psi_2\rangle$.

In similar spirit to the previous subsection, we have the following corollary to Theorem 4, which provides an exponential separation for a problem studied in[17] (see also[20]):

Corollary 4. An incoherent access QUALM calling the oracle at most k times, with $k < \left(2^\ell/\sqrt{6}\right)^{4/7}$, can only distinguish two state ensemble probability distributions ν_1 and ν_2 with bias $\leq \mathscr{O}(k^3/2^\ell)$ if both distributions are unitarily invariant, satisfying Supplementary Equation (125).

Proof. This corollary can be proven by contradiction. We choose a reference state $|0\rangle$. For each of the given state ensembles $\nu_1(|\psi_1\rangle, |\psi_2\rangle, ..., |\psi_k\rangle, ...)$ and $\nu_2(|\psi_1\rangle, |\psi_2\rangle, ..., |\psi_k\rangle, ...)$, we can define a probability distribution of unitaries U_i

$$\widetilde{\nu}_1(U_1, U_2, ..., U_k, ...) = \Omega^k \nu_1(U_1|0\rangle, U_2|0\rangle, ..., U_k|0\rangle, ...) \tag{126}$$
$$\widetilde{\nu}_2(U_1, U_2, ..., U_k, ...) = \Omega^k \nu_2(U_1|0\rangle, U_2|0\rangle, ..., U_k|0\rangle, ...)\,. \tag{127}$$

Here Ω is a constant normalization factor that takes care of the difference in integration measures of state integration and unitary operator integration. The integration over states is over the manifold $\mathbb{C}P\left(2^\ell - 1\right) = SU(2^\ell)/SU(2^\ell - 1)$, so

$$\int d\psi\, f(|\psi\rangle) = \frac{1}{\Omega}\int_{\text{Haar}} f(U|0\rangle)\, dU\,. \tag{128}$$

If an incoherent access QUALM can distinguish between these two lab oracles with a larger bias than $\mathscr{O}\left(k^3/2^n\right)$, then the same bias can be achieved for a different task of distinguishing the two ensembles of unitaries $\widetilde{\nu}_1$ and $\widetilde{\nu}_2$, since we could simply prepare input states $|0\rangle$ before each application of the lab oracle and then apply the QUALM that distinguishes the ensemble of states obtained this way. This is a contradiction with Corollary 3. □

Supplementary Note 4.3: Detecting non-unitarity

Another simple corollary that follows directly from Theorem 4 is the following:

Corollary 5. Consider a lab oracle LOD_ℓ which applies a completely depolarizing channel every time it is called, i.e. $\mathscr{E}_{\mathsf{L}}[\rho_{\mathsf{L}}] = \frac{\mathbb{1}}{2^\ell}$. For any incoherent access QUALM applied k times with with $k < \left(2^\ell/\sqrt{6}\right)^{4/7}$, LOD_ℓ and the fixed unitary lab oracle LOQ_ℓ cannot be distinguished with a bias bigger than $\mathscr{O}\left(k^3/2^\ell\right)$.

Proof. The proof of this corollary is almost trivial, since for any ρ,

$$\int_{\text{Haar}} dU\, U\rho U^\dagger = \frac{\mathbb{1}}{2^\ell}\,. \tag{129}$$

In words, averaging a state over random unitary evolution is equivalent to applying the completely depolarizing channel. The output of any incoherent access QUALM for LOD_ℓ is therefore identical to that of LOP_ℓ which uses independent random unitaries. Thus, we have reduced our problem to one previously solved. □

Supplementary Note 5: QUALM for symmetry of time evolution operator

Supplementary Note 5.1: Outline of problem

In this section, we will be inspired by a new question: given an experimental system evolved by a time-independent Hamiltonian H, how do we determine the symmetries of H? There are different kinds of symmetries we might be interested in, such as global and local symmetries. Clearly this is an experimentally relevant problem, as symmetries are key to understanding the physics of natural systems. We will address a toy version of the problem which itself may be useful in contemporary experiments.

We consider the three most commonly studied symmetry classes, known as the unitary, orthogonal and symplectic classes. These comprise a classification of different forms of time-reversal symmetry (or a lack thereof). The time evolution operator of a system without time-reversal symmetry belongs to the unitary group $U(D)$ when the Hilbert space dimension is D. The time-reversal transformation is an anti-unitary operator which satisfies $T(a|\psi_1\rangle + b|\psi_2\rangle) = a^* T|\psi_1\rangle + b^* T|\psi_2\rangle$,

and preserves the norm of the state. Time-reversal symmetry requires the time evolution operator U to satisfy[7] $UT = TU$, or equivalently

$$TUT = T^2 U \,. \tag{130}$$

Physically, every state should return to itself if we apply time-reversal twice, up to a phase, and so T^2 must be proportional to identity. It can be proven that the phase has to be ± 1 (simply because T^2 commutes with T), so that there are two classes of time-reversal symmetric systems.

If $T^2 = \mathbb{1}$, the canonical choice is $T = K$ where K is the complex conjugation operator defined with respect to the standard basis

$$K\left(\sum_x c_x |x\rangle\right) = \sum_x c_x^* |x\rangle \,. \tag{131}$$

In this case, $TUT = U^*$ and the time-reversal symmetry condition becomes $U^* = U$, so that U is a real matrix satisfying $U^T U = \mathbb{1}$, which belongs to the orthogonal group $O(D)$.

If $T^2 = -\mathbb{1}$, then T can be written as $T = \mathbf{J}K$ with $\mathbf{J}$ a real anti-symmetric matrix satisfying $\mathbf{J}^2 = -\mathbb{1}$. A standard choice is

$$\mathbf{J} := \begin{bmatrix} \mathbf{0}_{D/2} & \mathbb{1}_{D/2} \\ -\mathbb{1}_{D/2} & \mathbf{0}_{D/2} \end{bmatrix} \tag{132}$$

where $\mathbf{0}_{D/2}$ is the $D/2 \times D/2$ matrix of all zeros (note that D has to be even for this symmetry class). In this case, the time-reversal requirement becomes $-\mathbf{J}U^*\mathbf{J} = U$, or equivalently

$$-\mathbf{J}U^T\mathbf{J} = U^{-1} \,. \tag{133}$$

The unitaries satisfying this requirement form the compact symplectic group $\mathrm{Sp}(D/2)$.

Some well-known examples of orthogonal and symmetric matrices are realized for the unitary dynamics of a single $SU(2)$ spin. For a spin in a static magnetic field, the Hamiltonian is $H = \vec{h} \cdot \vec{S}$, with $\vec{S}$ the spin angular momentum operator. For integer spin, the time evolution operator e^{-iHt} is an orthogonal matrix, and for half odd integer spin (i.e., the value of the spin is in $\mathbb{Z}_{\geq 0} + \frac{1}{2}$) it is a symplectic matrix.

On each of the three Lie groups $U(D), O(D)$ and $\mathrm{Sp}(D/2)$, there is a unique Haar measure that is invariant under both left-translation and right-translation. Here, then, is the problem we will address: suppose we are given oracle access to a unitary which is either (i) a fixed Haar random unitary, (ii) a fixed Haar random orthogonal matrix, and (iii) a fixed Haar random symplectic matrix. Can we efficiently distinguish whether the oracle is of type (i), (ii), or (iii)? This is tantamount to determing the type of time-reversal symmetry (or lack thereof) of the unitary encoded in the oracle.

The answer is: for coherent access, we can efficiently distinguish between (i), (ii), (iii) using $\mathcal{O}(1)$ oracle queries and $\mathcal{O}(\ell)$ gates. For incoherent access, (i), (ii), and (iii) can only be reliably distinguished using $e^{\Omega(\ell)}$ oracle queries and as many gates. As such we have an exponential gap in the query complexity, and thus in the QUALM complexity, between the coherent and incoherent settings, when it comes to determining the type of time-reversal symmetry. The proofs of these statements generalize the proofs developed in previous sections. The main additional complication is that Weingarten functions for the orthogonal and symplectic Haar ensembles are more sophisticated than the ordinary Weingarten functions, although the basic architecture of the proofs is the same as those in Supplementary Note 3. We will draw extensively from the Weingarten function results in[24,48,73], and a review can be found in C.

Supplementary Note 5.2: Problem definition: distinguishing between symmetries of unitaries

The problem stated above involves distinguishing three lab oracles. Here is a precise statement of this task, which generalizes Definition 16:

[7] There is a difference between time-reversal symmetry of U and that of a Hamiltonian. We will not expand on this point and will focus on the requirement in Supplementary Equation (130).

Definition 23 (*The task of distinguishing between three lab oracles*). Given three lab oracles $\mathsf{LO}_0 = (\mathscr{E}_0, \rho_0)$, $\mathsf{LO}_1 = (\mathscr{E}_1, \rho_1)$, $\mathsf{LO}_2 = (\mathscr{E}_2, \rho_2)$, consider a task of the form: $(\mathbf{S}_{\text{in}}, \mathbf{S}_{\text{out}}, f, \mathscr{G})$ for some $\mathscr{G}$ where $\mathbf{S}_{\text{in}}$ is empty, $\mathbf{S}_{\text{out}}$ consists of two qubits, and

$$f : \mathscr{LO}(\mathbf{N}, \mathbf{L}) \to \{0, 1, 2\},$$

where $f(\mathsf{LO}_i) = i$.

As in Definition 16, we may have an error ε in the computation of this function; and just like in Definition 17, we can talk about a QUALM which outputs for each of the lab oracles a probability distribution over the possible outcomes $\{0, 1, 2\}$. If the minimal total variation distance between any pair of these three distributions is δ, we say that the QUALM achieves the task of distinguishing between these three lab oracles with bias δ. Yet again, this bias can be amplified by standard repetition and a simple follow up classical calculation.

Next we define the relevant lab oracles for the "symmetry distinction" problem:

Definition 24 (*The fixed random unitary lab oracles* $\mathsf{LO}_\ell^U, \mathsf{LO}_\ell^O, \mathsf{LO}_\ell^{\text{Sp}}$). For ℓ a positive integer, we define the lab oracles $\mathsf{LO}_\ell^U = (\mathbf{N}, \mathbf{L}, \mathscr{E}, \rho^U)$, $\mathsf{LO}_\ell^O = (\mathbf{N}, \mathbf{L}, \mathscr{E}, \rho^O)$, $\mathsf{LO}_\ell^{\text{Sp}} = (\mathbf{N}, \mathbf{L}, \mathscr{E}, \rho^{\text{Sp}})$ as follows:

- $\mathbf{N}$ is an $e^{\Omega(\ell)}$ qubit Hilbert space,
- $\mathbf{L}$ is an ℓ qubit Hilbert space with dimension $D = 2^\ell$,
- $\mathscr{E}$ is a unitary transformation which for U a unitary on ℓ qubits, and $|U\rangle$ a classical description of U on $e^{\mathscr{O}(\ell)}$ qubits (specified to within exponentially many bits of accuracy in each entry), does the following:

  $$\mathscr{E}(|U\rangle \otimes |\alpha\rangle) = |U\rangle \otimes U |\alpha\rangle$$

- The states $\rho^U, \rho^O, \rho^{\text{Sp}}$ are given by:
  - $\rho^U = \left(\int_{U(D)} |U\rangle \langle U| \, dU \right) \otimes |0^\ell\rangle \langle 0^\ell|$
  - $\rho^O = \left(\int_{O(D)} |O\rangle \langle O| \, dO \right) \otimes |0^\ell\rangle \langle 0^\ell|$
  - $\rho^{\text{Sp}} = \left(\int_{\text{Sp}(D/2)} |S\rangle \langle S| \, dS \right) \otimes |0^\ell\rangle \langle 0^\ell|$

Then the problem statement is as follows:

Problem statement (Symmetry distinction): Given a set of admissible gates, find a QUALM which implements the task of distinguishing between the three lab oracles $\mathsf{LO}_\ell^U, \mathsf{LO}_\ell^O, \mathsf{LO}_\ell^{\text{Sp}}$ with error at most $1/3$.

Having stated the problem precisely, we now give our main results:

Theorem 5. For all $\ell \geq 1$, there exists a QUALM_ℓ in the coherent access model which solve the symmetry distinction problem between $\mathsf{LO}_\ell^U, \mathsf{LO}_\ell^O, \mathsf{LO}_\ell^{\text{Sp}}$ and has $\mathscr{O}(\ell)$ QUALM complexity.

Theorem 6. Any incoherent access QUALM which makes $k < \left(2^\ell/12\right)^{2/7}$ queries to the lab oracle can at most distinguish between $\mathsf{LO}_\ell^U, \mathsf{LO}_\ell^O, \mathsf{LO}_\ell^{\text{Sp}}$ with a bias δ of order $O\left(k^3/2^\ell\right)$. Consequently, the QUALM query complexity for distinguishing these three lab oracles with constant error (Definition 23) is lower bounded by the exponential $\Omega(2^{2\ell/7})$.

These two theorems are clearly analogs of Theorems 3 and 4 above. Once again, the proof of Theorem 5 is easy (though slightly more complicated than that of Theorem 3) whereas the proof of the lower bound, Theorem 6, is far from trivial. In the following two subsections, we prove Theorems 5 and 6 in turn.

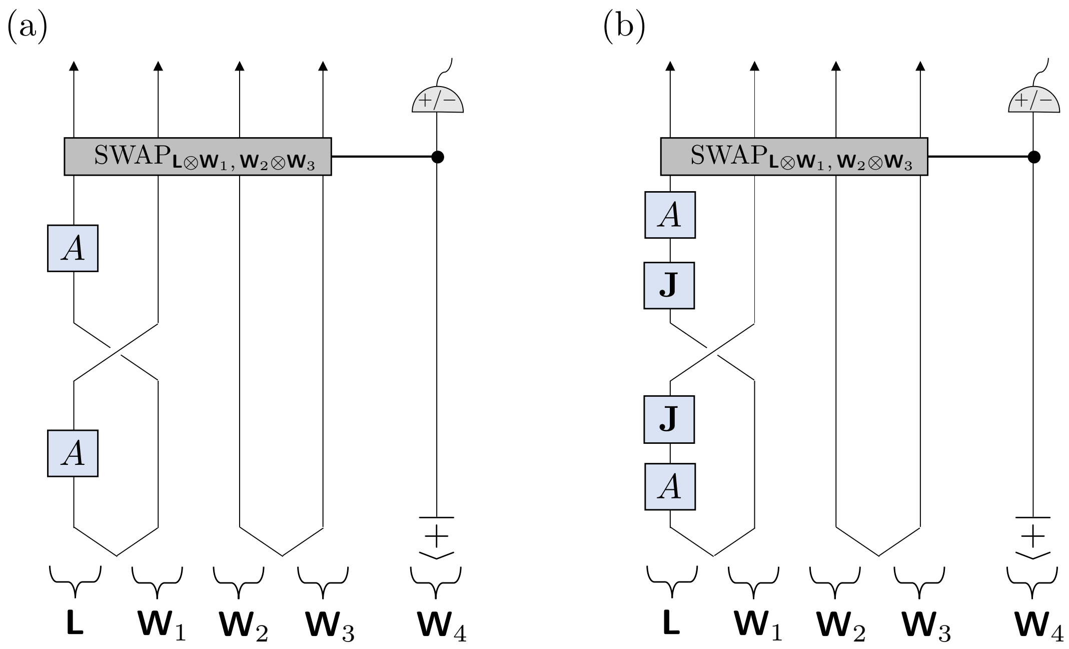

Supplementary Figure 9. *Coherent access QUALMs for symmetry distinction.* (a) Illustration of the coherent access QUALM that distinguishes between LO_ℓ^O from the other two lab oracles LO_ℓ^U and $\mathsf{LO}_\ell^{\mathrm{Sp}}$. (b) The coherent access QUALM that distinguishes between LO_ℓ^U and $\mathsf{LO}_\ell^{\mathrm{Sp}}$.

Supplementary Note 5.3: Proof of Theorem 5

Here we give an efficient QUALM which efficiently distinguishes $\mathsf{LO}_\ell^U, \mathsf{LO}_\ell^O, \mathsf{LO}_\ell^{\mathrm{Sp}}$. The method is essentially a variation of the SWAP test. This amounts to a proof of Theorem 5, and goes as follows.

Proof. Recall that L has size ℓ, and we let W have size $3\ell+1$. We write $\mathsf{W} \simeq \mathsf{W}_1 \otimes \mathsf{W}_2 \otimes \mathsf{W}_3 \otimes \mathsf{W}_4$ where $\mathsf{W}_1, \mathsf{W}_2, \mathsf{W}_3$ each contain ℓ qubits, and W_4 contains a single qubit. Here we provide a description of the QUALM. Letting $\{|x\rangle\}_{x\in\{0,1\}^\ell}$ be the standard basis on ℓ qubits, and letting $|+\rangle = \frac{1}{\sqrt{2}}(|0\rangle + |1\rangle)$ be a single qubit state, we prepare the state

$$|\Phi\rangle_{\mathsf{LW}} := \left(\frac{1}{\sqrt{D}} \sum_{x\in\{0,1\}^\ell} |x\rangle_{\mathsf{L}} \otimes |x\rangle_{\mathsf{W}_1}\right) \otimes \left(\frac{1}{\sqrt{D}} \sum_{y\in\{0,1\}^\ell} |y\rangle_{\mathsf{W}_2} \otimes |y\rangle_{\mathsf{W}_3}\right) \otimes |+\rangle_{\mathsf{W}_4}. \tag{134}$$

This state can be prepared from the all zero state using $2\ell+1$ Hadamard gates and 2ℓ CNOT gates. Next we query the lab oracle once and have it apply a unitary to L. For now, we label the applied unitary by A, to be agnostic as to whether it is a regular unitary, an orthogonal matrix, or a symplectic matrix. Then we swap the L qubits with the W_1 qubits (which

again requires $\mathscr{O}(\ell)$ 2-qubit gates), yielding

$$\begin{aligned}
&\mathrm{SWAP}_{\mathsf{L},\mathsf{W}_1}\left(\frac{1}{\sqrt{D}}\sum_{x\in\{0,1\}^\ell} A|x\rangle_{\mathsf{L}}\otimes|x\rangle_{\mathsf{W}_1}\right)\otimes\left(\frac{1}{\sqrt{D}}\sum_{y\in\{0,1\}^\ell}|y\rangle_{\mathsf{W}_2}\otimes|y\rangle_{\mathsf{W}_3}\right)\otimes|+\rangle_{\mathsf{W}_4}\\
&=\left(\frac{1}{\sqrt{D}}\sum_{x\in\{0,1\}^\ell}|x\rangle_{\mathsf{L}}\otimes A|x\rangle_{\mathsf{W}_1}\right)\otimes\left(\frac{1}{\sqrt{D}}\sum_{y\in\{0,1\}^\ell}|y\rangle_{\mathsf{W}_2}\otimes|y\rangle_{\mathsf{W}_3}\right)\otimes|+\rangle_{\mathsf{W}_4}\\
&=\left(\frac{1}{\sqrt{D}}\sum_{x\in\{0,1\}^\ell}A^T|x\rangle_{\mathsf{L}}\otimes |x\rangle_{\mathsf{W}_1}\right)\otimes\left(\frac{1}{\sqrt{D}}\sum_{y\in\{0,1\}^\ell}|y\rangle_{\mathsf{W}_2}\otimes|y\rangle_{\mathsf{W}_3}\right)\otimes|+\rangle_{\mathsf{W}_4}
\end{aligned} \tag{135}$$

where we have leveraged the identity[8]

$$\sum_{x\in\{0,1\}^\ell}|x\rangle\otimes A|x\rangle=\sum_{x\in\{0,1\}^\ell}A^T|x\rangle\otimes|x\rangle \tag{136}$$

for any A, where A^T is defined as the matrix transpose in the $|x\rangle$ basis, i.e. $\langle x_1|A^T|x_2\rangle=\langle x_2|A|x_1\rangle$. We query the oracle again, giving us

$$\left(\frac{1}{\sqrt{D}}\sum_{x\in\{0,1\}^\ell}AA^T|x\rangle_{\mathsf{L}}\otimes |x\rangle_{\mathsf{W}_1}\right)\otimes\left(\frac{1}{\sqrt{D}}\sum_{y\in\{0,1\}^\ell}|y\rangle_{\mathsf{W}_2}\otimes|y\rangle_{\mathsf{W}_3}\right)\otimes|+\rangle_{\mathsf{W}_4}. \tag{137}$$

Finally, we perform a controlled swap between $\mathsf{L}\otimes\mathsf{W}_1$ and $\mathsf{W}_2\otimes\mathsf{W}_3$, conditioned on the $|+\rangle$ qubit in W_4. Subsequently measuring the $|+\rangle$ qubit in the $\{|+\rangle,|-\rangle\}$ basis, we will measure

$$\Pr\big[\text{measure }+\big]=\frac{1}{2}\left(1+\left|\frac{1}{D}\mathrm{tr}\left\{AA^T\right\}\right|^2\right),\qquad \Pr\big[\text{measure }-\big]=\frac{1}{2}\left(1-\left|\frac{1}{D}\mathrm{tr}\left\{AA^T\right\}\right|^2\right). \tag{138}$$

If the lab oracle is LO_ℓ^O, then we measure $|+\rangle$ with probability one. However, if the lab oracle is LO_ℓ^U or $\mathsf{LO}_\ell^{\mathrm{Sp}}$, we will measure $|+\rangle$ and $|-\rangle$ with probabilities that are exponentially close to $1/2$ (i.e., exponentially suppressed in ℓ). To see this, recall Markov's inequality for a random variable X:

$$\Pr\left[X\geq C\right]\leq\frac{\mathbb{E}[X]}{C}. \tag{139}$$

Using this, we find in the unitary setting

$$\begin{aligned}
\Pr_{A\sim U(D)}\left[\left|\frac{1}{D}\mathrm{tr}\left\{AA^T\right\}\right|^2\geq\frac{1}{D}\right]&\leq D\int_{U(D)}dU\left|\frac{1}{D}\mathrm{tr}\left\{UU^T\right\}\right|^2\\
&=\frac{1}{D}\sum_{a,b,c,d}\int_{U(D)}dU\,U_{ab}U_{ab}U_{cd}^\dagger U_{cd}^\dagger\\
&=\frac{2}{D+1}
\end{aligned} \tag{140}$$

where we have used Supplementary Equation (209) to go from the second line to the third line. Similarly in the symplectic

[8] To see this, write $A=\sum_{x_1,x_2}A_{x_1x_2}|x_1\rangle\langle x_2|$ and observe that $\sum_x|x\rangle\otimes A|x\rangle=\sum_{x,x_1,x_2}|x\rangle\otimes A_{x_1x_2}|x_1\rangle\langle x_2|x\rangle=\sum_{x_1,x_2}A_{x_1x_2}|x_2\rangle\otimes|x_1\rangle=\sum_{x,x_1,x_2}A_{x_1x_2}|x_2\rangle\langle x_1|x\rangle\otimes|x\rangle=\sum_x A^T|x\rangle\otimes|x\rangle$.

setting,

$$\mathrm{Pr}_{A\sim \mathrm{Sp}(D/2)}\left[\left|\frac{1}{D}\mathrm{tr}\left\{AA^T\right\}\right|^2 \geq \frac{1}{D}\right] \leq D\int_{\mathrm{Sp}(D/2)} dS\left|\frac{1}{D}\mathrm{tr}\left\{SS^T\right\}\right|^2$$
$$= \frac{1}{D}\sum_{a,b,c,d}\int_{\mathrm{Sp}(D/2)} dS\, S_{ab}S_{ab}S^\dagger_{cd}S^\dagger_{cd}$$
$$= \frac{2}{D+1} \tag{141}$$

where Supplementary Equation (251) has been used to go from the second line to the third line. Comparing with (138), we see that in the unitary and symplectic cases $\Pr[\text{measure } +]$ and $\Pr[\text{measure } -]$ are exponentially close to $1/2$ with probability exponentially close to one.

In summary, with $\mathscr{O}(1)$ measurements, we can distinguish LO_ℓ^O from both of LO_ℓ^U and $\mathsf{LO}_\ell^{\mathrm{Sp}}$ with constant bias. If we measure $|-\rangle$ in our set of $\mathscr{O}(1)$ measurements, the lab oracle could be either LO_ℓ^U or $\mathsf{LO}_\ell^{\mathrm{Sp}}$. Then we repeat the procedure above, but with a minor alteration. After querying the oracle the first time, we apply $\mathbf{J}$ to L, giving us

$$\left(\frac{1}{\sqrt{D}}\sum_{x\in\{0,1\}^\ell}\mathbf{J}A|x\rangle_{\mathsf{L}}\otimes|x\rangle_{\mathsf{W}_1}\right)\otimes\left(\frac{1}{\sqrt{D}}\sum_{y\in\{0,1\}^\ell}|y\rangle_{\mathsf{W}_2}\otimes|y\rangle_{\mathsf{W}_3}\right)\otimes|+\rangle_{\mathsf{W}_4}\,. \tag{142}$$

Note that the application of $\mathbf{J}$ is easy, since $\mathbf{J} = i\sigma^y\otimes\mathbf{1}_2^{\otimes(\ell-1)}$. Next we swap L with W_1 as before. But before the second oracle query, we apply $\mathbf{J}$ to L, at this point giving us

$$\left(\frac{1}{\sqrt{D}}\sum_{x\in\{0,1\}^\ell}\mathbf{J}|x\rangle_{\mathsf{L}}\otimes\mathbf{J}A|x\rangle_{\mathsf{W}_1}\right)\otimes\left(\frac{1}{\sqrt{D}}\sum_{y\in\{0,1\}^\ell}|y\rangle_{\mathsf{W}_2}\otimes|y\rangle_{\mathsf{W}_3}\right)\otimes|+\rangle_{\mathsf{W}_4}$$
$$=\left(\frac{1}{\sqrt{D}}\sum_{x\in\{0,1\}^\ell}\mathbf{J}A^T\mathbf{J}^T|x\rangle_{\mathsf{L}}\otimes|x\rangle_{\mathsf{W}_1}\right)\otimes\left(\frac{1}{\sqrt{D}}\sum_{y\in\{0,1\}^\ell}|y\rangle_{\mathsf{W}_2}\otimes|y\rangle_{\mathsf{W}_3}\right)\otimes|+\rangle_{\mathsf{W}_4}$$
$$=\left(\frac{1}{\sqrt{D}}\sum_{x\in\{0,1\}^\ell}(-\mathbf{J}A^T\mathbf{J})|x\rangle_{\mathsf{L}}\otimes|x\rangle_{\mathsf{W}_1}\right)\otimes\left(\frac{1}{\sqrt{D}}\sum_{y\in\{0,1\}^\ell}|y\rangle_{\mathsf{W}_2}\otimes|y\rangle_{\mathsf{W}_3}\right)\otimes|+\rangle_{\mathsf{W}_4} \tag{143}$$

where we have used Supplementary Equation (136) as well as $\mathbf{J}^T = -\mathbf{J}$.

Then querying the oracle again, performing a controlled swap between $\mathsf{L}\otimes\mathsf{W}_1$ and $\mathsf{W}_2\otimes\mathsf{W}_3$ with $|+\rangle$ on W_4 being the control qubit, and subsequently measuring the control qubit in the $\{|+\rangle,|-\rangle\}$ basis, we find

$$\Pr[\text{measure } +] = \frac{1}{2}\left(1+\left|\frac{1}{D}\mathrm{tr}\left\{A(-\mathbf{J}A^T\mathbf{J})\right\}\right|^2\right)\,,\quad \Pr[\text{measure } -] = \frac{1}{2}\left(1-\left|\frac{1}{D}\mathrm{tr}\left\{A(-\mathbf{J}A^T\mathbf{J})\right\}\right|^2\right)\,. \tag{144}$$

Then if the lab oracle is $\mathsf{LO}_\ell^{\mathrm{Sp}}$ by Supplementary Equation (133) we will measure $|+\rangle$ with probability one, whereas if the lab oracle is LO_ℓ^U we will measure $|+\rangle$ and $|-\rangle$ with probabilities exponentially close to $1/2$. To see this, we again use the Markov inequality as

$$\mathrm{Pr}_{A\sim U(D)}\left[\left|\frac{1}{D}\mathrm{tr}\left\{A(-\mathbf{J}A^T\mathbf{J})\right\}\right|^2 \geq \frac{1}{D}\right] \leq D\int_{U(D)} dU\left|\frac{1}{D}\mathrm{tr}\left\{U(-\mathbf{J}U^T\mathbf{J})\right\}\right|^2$$
$$= \frac{1}{D}\sum_{a,b,c,d,e,f,g,h}\int_{U(D)} dU\, U_{ab}\mathbf{J}_{bc}U_{dc}\mathbf{J}_{da}U^\dagger_{ef}\mathbf{J}_{fg}U^\dagger_{hg}\mathbf{J}_{he}$$
$$= \frac{2}{D-1} \tag{145}$$

where we have used Supplementary Equation (209) to go from the second line to the third line. Then indeed, for Supplementary Equation (144) in the unitary case, $\Pr\big[\text{measure } +\big]$ and $\Pr\big[\text{measure } -\big]$ are exponentially close to $1/2$ with probability exponentially close to unity. As such, we can distinguish between $\mathsf{LO}_\ell^{\mathrm{Sp}}$ and LO_ℓ^{U} with constant bias.

In totality, we can efficiently distinguish between $\mathsf{LO}_\ell^{U}, \mathsf{LO}_\ell^{O}, \mathsf{LO}_\ell^{\mathrm{Sp}}$ with constant bias, using $\mathscr{O}(1)$ queries of the oracle and $\mathscr{O}(\ell)$ gates. □

Supplementary Note 5.4: Proof of Theorem 6

We use a similar proof strategy for Theorem 6 as we did for Theorem 4. The strategy in the latter case was to first prove Lemma 3 which established Theorem 4 in the simple measurement QUALM setting, and then to leverage Claim 2 to generalize to the full incoherent access QUALM setting. For Theorem 6, we first prove a Lemma which establishes the result in the simple measurement QUALM setting. Then again leveraging Claim 2, we obtain the full incoherent access QUALM setting.

So let us focus on establishing Theorem 6 for the simple measurement QUALM setting. Indeed, it suffices to prove that the following three probability distributions are exponentially close in total variational distance. Consider the probability distributions over k ℓ-bit strings, using the notation of Definition 20:

$$Q_k^U(s_0,s_1,\ldots,s_k) = \Pr(s_0)\cdot\left(\int_{U(D)} dU \prod_{i=1}^{k} \langle y^i_{s_0 s_1\ldots s_i}|U\sigma^{i-1}_{s_0 s_1\ldots s_{i-1}}U^\dagger|y^i_{s_0 s_1\ldots s_i}\rangle \lambda^i_{s_0 s_1\ldots s_i}\right) \tag{146}$$

$$Q_k^O(s_0,s_1,\ldots,s_k) = \Pr(s_0)\cdot\left(\int_{O(D)} dO \prod_{i=1}^{k} \langle y^i_{s_0 s_1\ldots s_i}|O\sigma^{i-1}_{s_0 s_1\ldots s_{i-1}}O^\dagger|y^i_{s_0 s_1\ldots s_i}\rangle \lambda^i_{s_0 s_1\ldots s_i}\right) \tag{147}$$

$$Q_k^{\mathrm{Sp}}(s_0,s_1,\ldots,s_k) = \Pr(s_0)\cdot\left(\int_{\mathrm{Sp}(D/2)} dS \prod_{i=1}^{k} \langle y^i_{s_0 s_1\ldots s_i}|S\sigma^{i-1}_{s_0 s_1\ldots s_{i-1}}S^\dagger|y^i_{s_0 s_1\ldots s_i}\rangle \lambda^i_{s_0 s_1\ldots s_i}\right) \tag{148}$$

where $\Pr(s_0) = |\langle y^0_{s_0}|0^{\mathsf{L}}\rangle|^2 \lambda^0_{s_0}$. In order to show that Q_k^U, Q_k^O, Q_k^{Sp} are all exponentially close in total variational distance, it suffices that they are all exponentially close to a single probability distribution

$$P_k(s_0,s_1,\ldots,s_k) = \Pr(s_0) D^{-k}\prod_{i=1}^{k}\lambda^i_{s_i} \tag{149}$$

since we can then use the triangle inequality to the bound the pairwise distances we seek.

We have the following Lemma, which generalizes Lemma 3:

Lemma 4. Let $Q_k^U, Q_k^O, Q_k^{\mathrm{Sp}}$ and P_k be defined as above, where $\left|y^i_{s_0 s_1\ldots s_i}\right\rangle$ are normalized pure states in L and $\sigma^{i-1}_{s_0 s_1\ldots s_{i-1}}$ are normalized density operators in the same Hilbert space. We have $0 \le \lambda^i_{s_0 s_1\ldots s_i} \le 1$ and $\sum_{s_i} \lambda^i_{s_0 s_1\ldots s_i} \left|y^i_{s_0 s_1\ldots s_i}\right\rangle\left\langle y^i_{s_0 s_1\ldots s_i}\right| = \mathbb{1}$. The lab subsystem L consists of ℓ qubits, and the number of queries to the lab oracle k satisfies $k < \left(2^\ell/12\right)^{2/7}$. Furthermore, $s_j = 1,2,\ldots,D_m$ is a set of labels for each $j = 0,1,\ldots,k$. Then

$$\|P_k - Q_k^U\|_1 = \sum_s \left|P_k(s) - Q_k^U(s)\right| \le \mathscr{O}(k^3/2^\ell) \tag{150}$$

$$\|P_k - Q_k^O\|_1 = \sum_s \left|P_k(s) - Q_k^O(s)\right| \le \mathscr{O}(k^3/2^\ell) \tag{151}$$

$$\|P_k - Q_k^{\mathrm{Sp}}\|_1 = \sum_s \left|P_k(s) - Q_k^{\mathrm{Sp}}(s)\right| \le \mathscr{O}(k^3/2^\ell)\,. \tag{152}$$

As remarked above, we see that Q_k^U, Q_k^O and Q_k^{Sp} are pairwise close to one another with distance $\mathscr{O}(1/2^\ell)$ by the triangle inequality.

The proof will require several facts about Weingarten functions of the orthogonal and symplectic ensembles. These are reviewed in a fair amount of detail in C, so presently we will just provide some basic notations and definitions. Let S_k denote the set of permutations on k elements, and let $P_2(2k)$ denote the set of pair permutations on $2k$ elements.

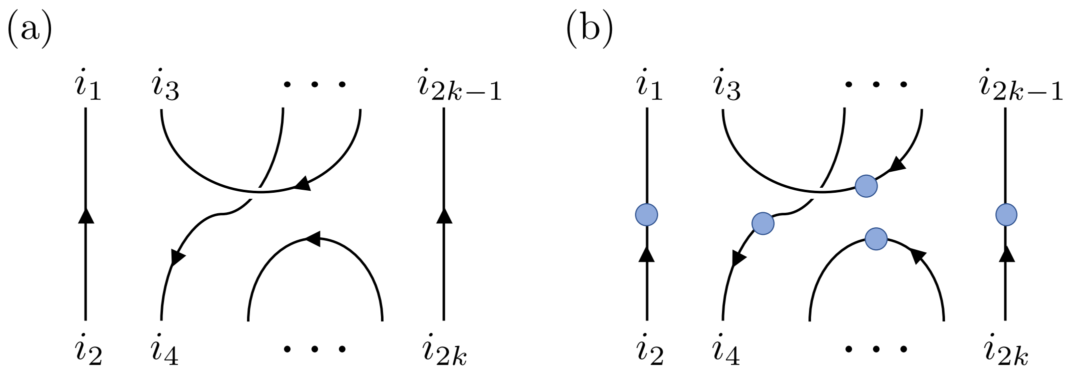

Supplementary Figure 10. *Matrix elements of* Δ*-operators.* Illustration of the matrix elements of the operators $\Delta_{\mathfrak{m}}$ and $\Delta'_{\mathfrak{m}}$ in Supplementary Equations (154) and (155), in (a) and (b) respectively. Fixed values of odd indices $i_1 i_3 \ldots i_{2k-1}$ specify a row of the matrix, and fixed values of the even indices $i_2 i_4 \ldots i_{2k}$ specify a column of the matrix. As usual, we use the tensor diagram notation explained in A. Each line in (a) represents a Kronecker δ, such as $\delta_{i_1 i_2}$. Each line in (b) with a blue solid circle represent a $\mathbf{J}$ matrix element such as $\mathbf{J}_{i_1 i_2}$.

Since $P_2(2k)$ is less familiar than the set of ordinary permutations, let us delve into it further. Recall that a pair permutation is a partition of $2k$ elements into k pairs. An element $\mathfrak{m} \in P_2(2k)$ is written as

$$\mathfrak{m} = \{\mathfrak{m}(1), \mathfrak{m}(2)\}\{\mathfrak{m}(3), \mathfrak{m}(4)\} \cdots \{\mathfrak{m}(2k-1), \mathfrak{m}(2k)\} \tag{153}$$

with the ordering convention $\mathfrak{m}(2i-1) < \mathfrak{m}(2i)$ for $1 \leq i \leq k$ and $\mathfrak{m}(1) < \mathfrak{m}(3) < \cdots < \mathfrak{m}(2k-1)$. We let $\mathfrak{e}$ denote the identity pairing $\mathfrak{e} = \{1,2\}\{3,4\} \cdots \{2k-1, 2k\}$. There is a canonical map from each $\mathfrak{m}$ to a unique permutation $\sigma_{\mathfrak{m}}$ in S_{2k} satisfying $\sigma_{\mathfrak{m}}(i) = \mathfrak{m}(i)$. We will often use $\mathfrak{m}$ and its counterpart in S_{2k} interchangeably.

For the proof, it will be convenient to define the following $D^k \times D^k$ matrices $\Delta_{\mathfrak{m}}$ and $\Delta'_{\mathfrak{m}}$ (see[24,73]). We let

$$[\Delta_{\mathfrak{m}}]^{i_2 i_4 \ldots i_{2k}}_{i_1 i_3 \ldots i_{2k-1}} = \prod_{s=1}^{k} \delta_{i_{\mathfrak{m}(2s-1)}, i_{\mathfrak{m}(2s)}} \tag{154}$$

and

$$\left[\Delta'_{\mathfrak{m}}\right]^{i_2 i_4 \ldots i_{2k}}_{i_1 i_3 \ldots i_{2k-1}} = \prod_{s=1}^{k} \mathbf{J}_{i_{\mathfrak{m}(2s-1)}, i_{\mathfrak{m}(2s)}}, \tag{155}$$

where $\mathbf{J}$ is the $D \times D$ canonical symplectic form in Supplementary Equation (132). We note that for $\mathfrak{m} = \mathfrak{e}$, we have

$$\Delta_{\mathfrak{e}} = \mathbb{1}^{\otimes k} \tag{156}$$

and

$$\Delta'_{\mathfrak{e}} = \mathbf{J}^{\otimes k}. \tag{157}$$

We will further use the multi-index notations $II' = (i_1, i'_1 \ldots, i_k, i'_k)$ and similarly and $A_{MN} = A_{m_1,n_1} A_{m_2,n_2} \cdots A_{m_k,n_k}$ for any $D \times D$ matrix A.

With these notations in mind, we commence with the proof of Lemma 4.

Proof. We have previously proven the Q^U_k case in Supplementary Equation (150) – this is the same as Lemma 3. But we still need to prove the Q^O_k and Q^{Sp}_k cases. In fact, the proof of the Q^U_k case in Lemma 3 is somewhat modular: it contains structure that is common to all three cases, with certain ingredients that need to be modified on a case-by-case basis.

We begin by defining

$$A_s = \bigotimes_{i=1}^{k} \sigma^{i-1}_{s_0 s_1 \ldots s_{i-1}}\,, \qquad B_s = \bigotimes_{i=1}^{k} |y^i_{s_0 s_1 \ldots s_i}\rangle\langle y^i_{s_0 s_1 \ldots s_i}|\, \lambda^i_{s_0 s_1 \ldots s_i} \tag{158}$$

so that

$$Q_k^U(s) = \sum_{I,J,I',J'} \int_{U(D)} dU\, U_{IJ} A_{s\,JI'} U^\dagger_{I'J'} B_{s\,J'I} \tag{159}$$

$$Q_k^O(s) = \sum_{I,J,I',J'} \int_{O(D)} dO\, O_{IJ} A_{s\,JI'} O^T_{I'J'} B_{s\,J'I} \tag{160}$$

$$Q_k^{\mathrm{Sp}}(s) = \sum_{I,J,K,L,M,N} \int_{Sp(D/2)} dS\, S_{IJ} A_{s\,JK} \mathbf{J}_{KL} S^T_{LM} \mathbf{J}^T_{MN} B_{s\,NI}\,. \tag{161}$$

In C we use the Weingarten calculus to arrive at the following equalities (see Supplementary Equations (203), (229), and (247) and the discussions surrounding them):

$$Q_k^U(s) = \sum_{\sigma,\tau \in S_k} \mathrm{tr}(\sigma A_s)\, \mathrm{tr}(\tau^{-1} B_s)\, \mathrm{Wg}^U(\tau\sigma^{-1}, D) \tag{162}$$

$$Q_k^O(s) = \sum_{\mathfrak{m},\mathfrak{n} \in P_2(2k)} \mathrm{tr}(\Delta_{\mathfrak{m}} A_s)\, \mathrm{tr}(\Delta_{\mathfrak{n}} B_s)\, \mathrm{Wg}^O(\sigma_{\mathfrak{n}}\sigma_{\mathfrak{m}}^{-1}, D) \tag{163}$$

$$Q_k^O(s) = \sum_{\mathfrak{m},\mathfrak{n} \in P_2(2k)} \mathrm{tr}(\Delta'_{\mathfrak{m}} A_s \mathbf{J}^{\otimes k})\, \mathrm{tr}(\Delta'_{\mathfrak{n}} \mathbf{J}^{T\otimes k} B_s)\, \mathrm{Wg}^{\mathrm{Sp}}(\sigma_{\mathfrak{n}}\sigma_{\mathfrak{m}}^{-1}, D/2)\,. \tag{164}$$

Noting that $|\mathrm{tr}(A_s)|, |\mathrm{tr}(B_s)|, |\mathrm{tr}(\Delta_{\mathfrak{e}} A_s)|, |\mathrm{tr}(\Delta_{\mathfrak{e}} B_s)|, |\mathrm{tr}(\Delta'_{\mathfrak{e}} A_s \mathbf{J}^{\otimes k})|, |\mathrm{tr}(\Delta'_{\mathfrak{e}} \mathbf{J}^{T\otimes k} B_s)| = 1$, we can leverage the expressions for $Q_k^U(s), Q_k^O(s), Q_k^{\mathrm{Sp}}(s)$ above to find the following inequalities:

$$\begin{aligned} |Q_k^U(s) - P_k(s)| \le |\mathrm{Wg}^U(\mathbb{1}, D) - D^{-k}| &+ \sum_{\sigma}\sum_{\tau\neq\mathbb{1}} |\mathrm{tr}(\sigma A_s)|\,|\mathrm{tr}(\tau^{-1} B_s)|\,|\mathrm{Wg}^U(\tau\sigma^{-1}, D)| \\ &+ \sum_{\sigma\neq\mathbb{1}} |\mathrm{tr}(\sigma A_s)|\,|\mathrm{Wg}^U(\sigma^{-1}, D)|\,, \end{aligned} \tag{165}$$

$$\begin{aligned} |Q_k^O(s) - P_k(s)| \le |\mathrm{Wg}^O(\sigma_{\mathfrak{e}}, D) - D^{-k}| &+ \sum_{\mathfrak{m}}\sum_{\mathfrak{n}\neq\mathfrak{e}} |\mathrm{tr}(\Delta_{\mathfrak{m}} A_s)|\,|\mathrm{tr}(\Delta_{\mathfrak{n}} B_s)|\,|\mathrm{Wg}^O(\sigma_{\mathfrak{n}}\sigma_{\mathfrak{m}}^{-1}, D)| \\ &+ \sum_{\mathfrak{m}\neq\mathfrak{e}} |\mathrm{tr}(\Delta_{\mathfrak{m}} A_s)|\,|\mathrm{Wg}^O(\sigma_{\mathfrak{m}}^{-1}, D)|\,, \end{aligned} \tag{166}$$

$$\begin{aligned} |Q_k^{\mathrm{Sp}}(s) - P_k(s)| \le |\mathrm{Wg}^{\mathrm{Sp}}(\sigma_{\mathfrak{e}}, D/2) - D^{-k}| &+ \sum_{\mathfrak{m}}\sum_{\mathfrak{n}\neq\mathfrak{e}} |\mathrm{tr}(\Delta'_{\mathfrak{m}} A_s \mathbf{J}^{\otimes k})|\,|\mathrm{tr}(\Delta_{\mathfrak{n}} \mathbf{J}^{T\otimes k} B_s)|\,|\mathrm{Wg}^{\mathrm{Sp}}(\sigma_{\mathfrak{n}}\sigma_{\mathfrak{m}}^{-1}, D/2)| \\ &+ \sum_{\mathfrak{m}\neq\mathfrak{e}} |\mathrm{tr}(\Delta'_{\mathfrak{m}} A_s \mathbf{J}^{\otimes k})|\,|\mathrm{Wg}^{\mathrm{Sp}}(\sigma_{\mathfrak{m}}^{-1}, D/2)|\,. \end{aligned} \tag{167}$$

To derive these inequalities, we have repeatedly used the triangle inequality, along with a convenient rearranging of the sums. This is directly analogous to the derivation of (54) (in fact, (165) is the same as (54)). To simplify the inequalities above, we will need that $|\mathrm{tr}(\sigma A_s)| \le 1$, $|\mathrm{tr}(\Delta_{\mathfrak{n}} A_s)| \le 1$, $|\mathrm{tr}(\Delta'_{\mathfrak{n}} A_s \mathbf{J}^{\otimes k})| \le 1$. The arguments for these three inequalities are given below:

1. In the unitary case, $|\mathrm{tr}(\sigma A_s)| \le 1$ since it is the absolute value of a product of Hilbert-Schmidt inner products of normalized density matrices.

2. In the orthogonal case, we bound $|\mathrm{tr}(\Delta_{\mathfrak{m}} A_s)|$ as follows. Recalling that A_s is a tensor product of k density matrices,

we can write each constituent density matrix in its respective eigenbasis so that we have

$$\begin{aligned} A_s &= \sum_{j_1,j_2,\dots,j_k} p^{(1)}_{j_1} p^{(2)}_{j_2} \cdots p^{(k)}_{j_k} |\phi^{(1)}_{j_1}\rangle\langle\phi^{(1)}_{j_1}| \otimes |\phi^{(2)}_{j_2}\rangle\langle\phi^{(2)}_{j_2}| \otimes \cdots \otimes |\phi^{(k)}_{j_k}\rangle\langle\phi^{(k)}_{j_k}| \\ &= \sum_{j_1,j_2,\dots,j_k} p^{(1)}_{j_1} p^{(2)}_{j_2} \cdots p^{(k)}_{j_k} \Phi_{j_1 j_2 \cdots j_k} \,. \end{aligned} \tag{168}$$

We have introduced $\Phi_{j_1 j_2 \cdots j_k}$ to compress the notation slightly. Now notice that

$$|\mathrm{tr}(\Delta_{\mathfrak{m}} A_s)| \le \sum_{j_1,j_2,\dots,j_k} p^{(1)}_{j_1} p^{(2)}_{j_2} \cdots p^{(k)}_{j_k} \, |\mathrm{tr}(\Delta_{\mathfrak{m}} \Phi_{j_1 j_2 \cdots j_k})| \,. \tag{169}$$

Here $\mathrm{tr}(\Delta_{\mathfrak{m}} \Phi_{j_1 j_2 \cdots j_k})$ is just a product of terms of the form $\langle \phi^{\ell}_{j_\ell} | \phi^{\ell'}_{j_{\ell'}} \rangle$, $\langle \phi^{\ell}_{j_\ell} | (|\phi^{\ell'}_{j_{\ell'}}\rangle)^*$, $(\langle \phi^{\ell}_{j_\ell}|)^* |\phi^{\ell'}_{j_{\ell'}}\rangle$, or $(\langle \phi^{\ell}_{j_\ell} | \phi^{\ell'}_{j_{\ell'}}\rangle)^*$; these are just inner products of pure states. But for any two pure (normalized) states $|\phi\rangle, |\phi'\rangle$, we have $|\langle \phi | \phi' \rangle| \le 1$, and so $|\mathrm{tr}(\Delta_{\mathfrak{m}} \Phi_{j_1 j_2 \cdots j_k})| \le 1$. Accordingly,

$$|\mathrm{tr}(\Delta_{\mathfrak{m}} A_s)| \le \sum_{j_1,j_2,\dots,j_k} p^{(1)}_{j_1} p^{(2)}_{j_2} \cdots p^{(k)}_{j_k} = 1 \,, \tag{170}$$

thus establishing $|\mathrm{tr}(\Delta_{\mathfrak{m}} A_s)| \le 1$.

3. In the symplectic case, we consider $|\mathrm{tr}(\Delta'_{\mathfrak{m}} A_s \mathbf{J}^{\otimes k})|$. We can bound this with the same strategy as the orthogonal case above, since $\mathrm{tr}(\Delta'_{\mathfrak{m}} \Phi_{j_1 j_2 \cdots j_k} \mathbf{J}^{\otimes k})$ is also a product of inner products of pure states (recall that $\mathbf{J}$ is unitary). Thus $|\mathrm{tr}(\Delta'_{\mathfrak{m}} A_s \mathbf{J}^{\otimes k})| \le 1$.

As such, we have the needed bounds in all three cases. Using the bounds in Supplementary Equations (165), (166) and (167), the Cauchy-Schwarz inequality, and some basic properties of the Weingarten functions (i.e., we perform manipulations directly analogous to the derivation of (56) in the proof of Lemma 3), we find

$$|Q^U_k(s) - P_k(s)| \le |\mathrm{Wg}^U(\mathbb{1}, D) - D^{-k}| + \sum_{\sigma} |\mathrm{Wg}^U(\sigma, D)| \sum_{\tau \ne \mathbb{1}} |\mathrm{tr}(\tau^{-1} B_s)| + \sum_{\sigma \ne \mathbb{1}} |\mathrm{Wg}^U(\sigma, D)| \,, \tag{171}$$

$$|Q^O_k(s) - P_k(s)| \le |\mathrm{Wg}^U(\sigma_{\mathfrak{e}}, D) - D^{-k}| + \sum_{\mathfrak{m}} |\mathrm{Wg}^O(\sigma_{\mathfrak{m}}, D)| \sum_{\mathfrak{n} \ne \mathfrak{e}} |\mathrm{tr}(\Delta_{\mathfrak{n}} B_s)| + \sum_{\mathfrak{m} \ne \mathfrak{e}} |\mathrm{Wg}^O(\sigma_{\mathfrak{m}}, D)| \,, \tag{172}$$

$$\begin{aligned} |Q^{\mathrm{Sp}}_k(s) - P_k(s)| \le |\mathrm{Wg}^U(\sigma_{\mathfrak{e}}, D/2) - D^{-k}| + \sum_{\mathfrak{m}} |\mathrm{Wg}^{\mathrm{Sp}}(\sigma_{\mathfrak{m}}, D/2)| \sum_{\mathfrak{n} \ne \mathfrak{e}} |\mathrm{tr}(\Delta'_{\mathfrak{n}} \mathbf{J}^{T \otimes k} B_s)| \\ + \sum_{\mathfrak{m} \ne \mathfrak{e}} |\mathrm{Wg}^{\mathrm{Sp}}(\sigma_{\mathfrak{m}}, D/2)| \,. \end{aligned} \tag{173}$$

We organize the above information by writing

$$\sum_s |Q^U_k(s) - P_k(s)| \le c^U_1 + c^U_2 T^U \tag{174}$$

$$\sum_s |Q^O_k(s) - P_k(s)| \le c^O_1 + c^O_2 T^O \tag{175}$$

$$\sum_s |Q^{\mathrm{Sp}}_k(s) - P_k(s)| \le c^{\mathrm{Sp}}_1 + c^{\mathrm{Sp}}_2 T^{\mathrm{Sp}} \tag{176}$$

where

$$c_1^U = D^k\left(|\mathrm{Wg}^U(\mathbb{1},D)-D^{-k}| + \sum_{\sigma\neq\mathbb{1}}|\mathrm{Wg}^U(\sigma,D)|\right),\; c_2^U = D^k\sum_{\sigma}|\mathrm{Wg}^U(\sigma,D)|,\; T^U = \frac{1}{D^k}\sum_{\tau\neq\mathbb{1}}\sum_s|\mathrm{tr}(\tau^{-1}B_s)| \tag{177}$$

$$c_1^O = D^k\left(|\mathrm{Wg}^O(\sigma_{\mathfrak{e}},D)-D^{-k}| + \sum_{\mathfrak{m}\neq\mathfrak{e}}|\mathrm{Wg}^O(\sigma_{\mathfrak{m}},D)|\right),\; c_2^O = D^k\sum_{\mathfrak{m}}|\mathrm{Wg}^O(\sigma_{\mathfrak{m}},D)|,\; T^O = \frac{1}{D^k}\sum_{\mathfrak{n}\neq\mathfrak{e}}\sum_s|\mathrm{tr}(\Delta_{\mathfrak{n}}B_s)| \tag{178}$$

$$c_1^{\mathrm{Sp}} = D^k\left(|\mathrm{Wg}^{\mathrm{Sp}}(\sigma_{\mathfrak{e}},D)-D^{-k}| + \sum_{\mathfrak{m}\neq\mathfrak{e}}|\mathrm{Wg}^{\mathrm{Sp}}(\sigma_{\mathfrak{m}},D/2)|\right),\; c_2^{\mathrm{Sp}} = D^k\sum_{\mathfrak{m}}|\mathrm{Wg}^{\mathrm{Sp}}(\sigma_{\mathfrak{m}},D/2)|,$$
$$T^{\mathrm{Sp}} = \frac{1}{D^k}\sum_{\mathfrak{n}\neq\mathfrak{e}}\sum_s|\mathrm{tr}(\Delta'_{\mathfrak{n}}\mathbf{J}^{T\otimes k}B_s)|. \tag{179}$$

So we need to appropriately bound the c_1's, c_2's and T's. We do so in turn.

Bounding the c_1's. We use the standard bounds on Weingarten functions (discussed in C).

1. In the unitary case, for $k < \left(2^\ell/\sqrt{6}\right)^{4/7}$, we have $|\mathrm{Wg}^U(\mathbb{1},D)-D^{-k}| = \mathscr{O}(k^{7/2}D^{-(k+2)})$ by Lemma 5 in C.1. Additionally, $\sum_{\sigma\in S_k}|\mathrm{Wg}^U(\sigma,D)| = \frac{(D-k)!}{D!} = D^{-k}+\mathscr{O}(k^2D^{-(k+1)})$ by Lemma 6 in C.1. Then $c_1^U = \mathscr{O}(k^{7/2}D^{-2})$.
2. In the orthogonal case, for $k < \left(2^\ell/12\right)^{2/7}$, we have $|\mathrm{Wg}^O(\sigma_{\mathfrak{e}},D)-D^{-k}| = \mathscr{O}(k^7D^{-(k+2)})$ by Lemma 7 in C.2. We also have $\sum_{\mathfrak{m}\in P_2(2k)}|\mathrm{Wg}^O(\sigma_{\mathfrak{m}},2D)| = \frac{(D-k)!!}{D!!} = D^{-k}+\mathscr{O}(k^2D^{-(k+1)})$ by Lemma 8 in C.2. Taken together, we find $c_1^O = \mathscr{O}(k^7D^{-2})$.
3. In the symplectic case, for $k < \left(2^\ell/6\right)^{2/7}$, we have $|\mathrm{Wg}^{\mathrm{Sp}}(\sigma_{\mathfrak{e}},D/2)-D^{-k}| = \mathscr{O}(k^{7/2}D^{-(k+2)})$ by Lemma 9 in C.3. Furthermore, we have $\sum_{\mathfrak{m}\in P_2(2k)}|\mathrm{Wg}^{\mathrm{Sp}}(\sigma_{\mathfrak{m}},D/2)| = \prod_{j=0}^{k-1}\frac{1}{D+2j} = D^{-k}+\mathscr{O}(k^2D^{-(k+1)})$ by Lemma 10 in C.3. Altogether, $c_1^{\mathrm{Sp}} = \mathscr{O}(k^{7/2}D^{-2})$.

For $k < \left(2^\ell/12\right)^{2/7}$, all three conditions above are applicable.

Bounding the c_2's. Here we can use the same bounds (i) $\sum_{\sigma\in S_k}|\mathrm{Wg}^U(\sigma,D)| = D^{-k}+\mathscr{O}(k^2D^{-(k+1)})$, (ii) $\sum_{\mathfrak{m}\in P_2(2k)}|\mathrm{Wg}^O(\sigma_{\mathfrak{m}},2D)| = D^{-k}+\mathscr{O}(k^2D^{-(k+1)})$, and (iii) $\sum_{\mathfrak{m}\in P_2(2k)}|\mathrm{Wg}^{\mathrm{Sp}}(\sigma_{\mathfrak{m}},D/2)| = D^{-k}+\mathscr{O}(k^2D^{-(k+1)})$ that we used for the c_1's. Multiplying (i), (ii) and (iii) by D^k, we find:

1. $c_2^U = 1+\mathscr{O}(k^2D^{-1})$.
2. $c_2^O = 1+\mathscr{O}(k^2D^{-1})$.
3. $c_2^{\mathrm{Sp}} = 1+\mathscr{O}(k^2D^{-1})$.

Finally, we need to bound the T's.

Bounding the T's.

1. The unitary case was already established in Lemma 3 above, and we found the bound $\frac{1}{D^k}\sum_s\sum_{\tau\neq\mathbb{1}}|\mathrm{tr}(\tau^{-1}B_s)| \leq \mathscr{O}\left(k^3/D\right)$.

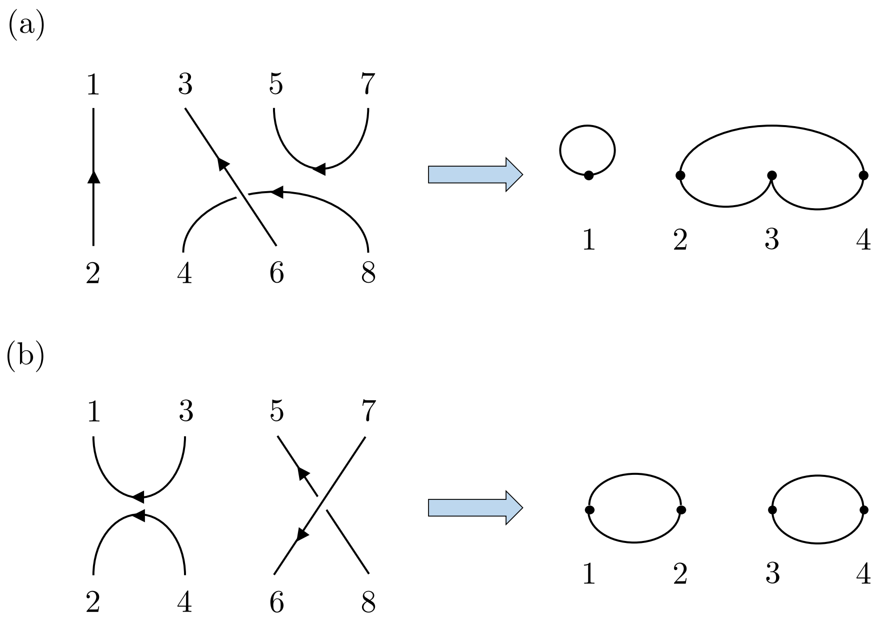

Supplementary Figure 11. *Two examples of the procedure that maps pair partitions $\mathfrak{m}$ to unoriented loops.* (a) $\mathfrak{m} = \{12\}\{36\}\{48\}\{57\}$ corresponds to (1)(234). (b) $\mathfrak{m} = \{13\}\{24\}\{58\}\{67\}$ corresponds to (12)(34).

2. Now we treat the orthogonal case. It is convenient to define the following $2k$ states:

$$|\psi_{2i-1}\rangle = \left|y^i_{s_0 s_1 \ldots s_i}\right\rangle, \; |\psi_{2i}\rangle = \left|y^i_{s_0 s_1 \ldots s_i}\right\rangle^* . \tag{180}$$

Then the trace is a product of state overlaps similar to the unitary case:

$$|\mathrm{tr}\,(\Delta_{\mathfrak{m}} B_s)| = \prod_{i=1}^{k} \left|\left\langle \psi_{\mathfrak{m}(2i-1)}\right| \left(\left|\psi_{\mathfrak{m}(2i)}\right\rangle^*\right)\right| \lambda^i_{s_0 s_1 \ldots s_i} \tag{181}$$

Now we define a procedure that relates each $\mathfrak{m}$ to loops in a k-vertex graph. Take k vertices, and add an unoriented link between i and j if $\mathfrak{m}$ contains one of the following pairs: $\{2i-1, 2j-1\}$, $\{2i-1, 2j\}$, $\{2i, 2j-1\}$, $\{2i, 2j\}$. $\mathfrak{m}$ could contain two of these pairs. For example if $\mathfrak{m}$ contains $\{13\}\{24\}$, we should connect two links between $1,2$ in the graph. As is illustrated in Supplementary Figure 11, pictorially the graph is obtained by simply replacing a pair of points $2i-1, 2i$ in the diagram representing $\Delta_{\mathfrak{m}}$ by a single point. This procedure leads to a graph with two unoriented links at each vertex, which therefore consists of unoriented loops. For example, $\mathfrak{m} = \{12\}\{36\}\{48\}\{57\}$ (see Figure 11(a)) corresponds to loops $(1)(234)$. Each trivial loop contributes 1 in the product, and the contribution corresponding to a nontrivial loop is very similar to the unitary case with a permutation with the same cycle, with the only difference being that some states are transformed by complex conjugation, following Supplementary Equations (180) and (181). For example, $\mathfrak{m} = \{12\}\{36\}\{48\}\{57\}$ corresponds to

$$|\mathrm{tr}\,(\Delta_{\mathfrak{m}} B_s)| = \left|\left\langle y^2_{s_0 s_1 s_2}\middle| y^3_{s_0 \ldots s_3}\right\rangle \left\langle y^{2*}_{s_0 s_1 s_2}\middle| y^4_{s_0 \ldots s_4}\right\rangle \left\langle y^3_{s_0 \ldots s_3}\middle| y^{4*}_{s_0 \ldots s_4}\right\rangle\right| \prod_{i=1}^{4} \lambda^i_{s_0 \ldots s_4} \tag{182}$$

where we have denoted $\left|y^{i*}_{s_0 \ldots s_i}\right\rangle$ for $\left(\left|y^i_{s_0 \ldots s_i}\right\rangle\right)^*$ and similarly for the bras, for simplicity. Then we can apply the same loop decomposition procedure as in the proof of Lemma 3. The only difference is the states in the matrix M_{ji} in Supplementary Equation (66) get complex conjugated in some cases. Since the complex conjugation does not change the orthonormality condition, the proof goes through, which gives the following inequality:

$$\sum_s |\mathrm{tr}\,(\Delta_{\mathfrak{m}} B_s)| \le D^{k - \left\lfloor \frac{L_{\mathfrak{m}}}{2} \right\rfloor} \tag{183}$$

with $L_{\mathfrak{m}}$ the total length of nontrivial loops obtained in the procedure above. Carrying out the sum over $\mathfrak{m}$ we obtain

$$T^{O} = \frac{1}{D^k} \sum_{\mathfrak{m}\neq\mathfrak{e}} \sum_{s} \left|\mathrm{tr}\left(\Delta_{\mathfrak{m}} B_s\right)\right| \leq \frac{1}{D^k} \sum_{\mathfrak{m}\neq\mathfrak{e}} D^{k-\lfloor \frac{L_{\mathfrak{m}}}{2} \rfloor} . \tag{184}$$

The number of pairings of $2k$ items where $k-L$ of the pairings are trivial (i.e., a pairing of the form $\{2i-1, 2i\}$) is given by

$$N^{\text{pair}}(2k, L) = \binom{k}{L} (2L-2)!! = \binom{k}{L} 2^{L-1} (L-1)! \tag{185}$$

Thus

$$T^{O} \leq \frac{1}{D^k} \sum_{L=1}^{k} N^{\text{pair}}(2k, L) D^{k-\lfloor \frac{L}{2} \rfloor} \leq \frac{1}{2} \frac{(1+2k)\frac{4k^2}{D}}{1-\frac{4k^2}{D}} = \frac{4k^3}{D} + \frac{2k^2}{D} + \mathscr{O}\left(\frac{k^5}{D^2}\right) . \tag{186}$$

3. The symplectic case is very similar to the orthogonal case. We define $2k$ states

$$|\psi_{2i-1}\rangle = \left|y^{i}_{s_0 s_1 \ldots s_i}\right\rangle , \; |\psi_{2i}\rangle = \mathbf{J} \left|y^{i}_{s_0 s_1 \ldots s_i}\right\rangle^{*} . \tag{187}$$

The trace becomes

$$|\mathrm{tr}(\Delta'_{\mathfrak{m}} \mathbf{J}^{T\otimes k} B)| = \prod_{i=1}^{k} \left| \left\langle \psi_{\mathfrak{m}(2i-1)} \right| \left(\mathbf{J}^{T} \left| \psi_{\mathfrak{m}(2i)} \right\rangle^{*} \right) \right| \lambda^{i}_{s_0 s_1 \ldots s_i} . \tag{188}$$

Following the same procedure as the orthogonal case, we can map each $\mathfrak{m}$ to a graph with unoriented loops. The remainder of the proof is identical, leading to the same upper bound

$$T^{Sp} \leq \frac{4k^3}{D} + \frac{2k^2}{D} + \mathscr{O}\left(\frac{k^5}{D^2}\right) \tag{189}$$

Putting all of the bounds on the c_1's, c_2's, and T's together, we find

$$\|P_k - Q_k^{U}\|_1 = \sum_{s} \left|P_k(s) - Q_k^{U}(s)\right| \leq \mathscr{O}(k^3/D) \tag{190}$$

$$\|P_k - Q_k^{O}\|_1 = \sum_{s} \left|P_k(s) - Q_k^{O}(s)\right| \leq \mathscr{O}(k^3/D) \tag{191}$$

$$\|P_k - Q_k^{\mathrm{Sp}}\|_1 = \sum_{s} \left|P_k(s) - Q_k^{\mathrm{Sp}}(s)\right| \leq \mathscr{O}(k^3/D) . \tag{192}$$

These are the desired bounds. □

Finally, using Claim 2, we can upgrade the simple measurement QUALM setting of Lemma 4 to prove the incoherent access QUALM setting of Theorem 6.

Supplementary Note A: Diagrams of quantum circuits and quantum channels

Here we explain our diagrammatic notation for quantum circuits and quantum channels used in the figures in the main text. First, we consider standard circuit notation for a quantum state $|\psi\rangle$, with two unitaries U_1, U_2 acting on it:

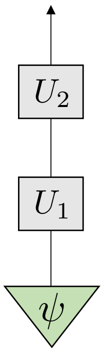

$$= \quad U_2 U_1 |\psi\rangle \tag{193}$$

This generalizes in the obvious way to any number of applied unitaries $U_1, U_2, U_3, \dots$. Notice that the wire in the diagram is directed, as indicated by the arrow at the top. The arrow designates the direction of matrix multiplication. For instance, a matrix M_α^β is represented by a box with one in-line and one out-line, where the in-line corresponds to the α index and the out-line corresponds to the β index.

Suppose that we want to consider the evolution of a density matrix ρ instead of a pure state $|\psi\rangle$. A diagrammatic representation analogous to the above is

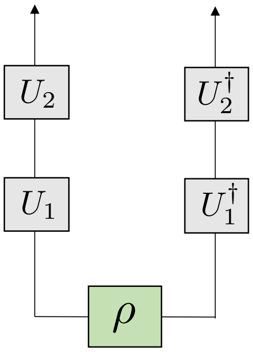

$$= \quad U_2 U_1 \, \rho \, U_1^\dagger U_2^\dagger \tag{194}$$

where ρ has two wires since it is a matrix (which by definition has two indices). We can likewise generalize the above to any sequence of applied unitaries.

It will be useful to simplify our diagrammatic notation slightly. Consider the quantum channel $\mathscr{U}$ which acts on a density matrix ρ by $\mathscr{U}[\rho] = U\rho U^\dagger$ for some unitary U. We the notation $\mathscr{U}_k[\rho] = U_k \rho U_k^\dagger$. Since these channels maps matrices to matrices, they have two in-wires and two out-wires. Thus we can depict $\mathscr{U}_2 \circ \mathscr{U}_1[\rho] = U_2 U_1 \rho U_1^\dagger U_2^\dagger$ by the

diagram

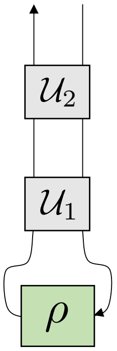

$$= \quad \mathcal{U}_2 \circ \mathcal{U}_1[\rho] \tag{195}$$

We make a further simplification by denoting pairs of wires (one on the left oriented upwards, and one on the right oriented downwards) by a single thick wire:

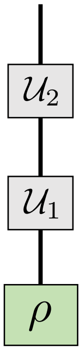

$$= \quad \mathcal{U}_2 \circ \mathcal{U}_1[\rho] \tag{196}$$

This generalizes to arbitrary superoperators $\mathscr{S}_1, \mathscr{S}_2, \ldots$ as

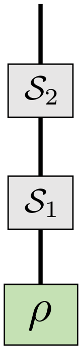

$$= \quad \mathcal{S}_2 \circ \mathcal{S}_1[\rho] \tag{197}$$

which is the primary diagrammatic notation used in the present paper.

There is an obvious generalization of the above "thick line" diagrams when we have multiple subsystems of our Hilbert space. For instance, suppose $\mathbf{H} \simeq \mathbf{A} \otimes \mathbf{B}$, and we have two quantum channels $\mathscr{C}_1, \mathscr{C}_2$ on $\mathbf{H}$. If we act these channels on

$\rho_{\mathbf{A}} \otimes \rho_{\mathbf{B}}$, then this is depicted diagrammatically as

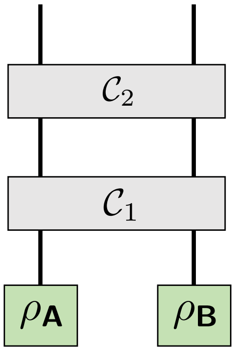

$$= \quad \mathcal{C}_2 \circ \mathcal{C}_1[\rho_{\mathbf{A}} \otimes \rho_{\mathbf{B}}] \tag{198}$$

where evidently each thick line corresponds to a subsystem. This of course works for N subsystems, giving us as many thick lines.

We will use an additional piece of diagrammatic notation, which is often useful. Again suppose $\mathbf{H} \simeq \mathbf{A} \otimes \mathbf{B}$. Say we have a collection of superoperators $\{\mathscr{S}_\alpha\}_\alpha$ acting on $\mathbf{A}$, and a collection of superoperators $\{\mathscr{S}'_\alpha\}_\alpha$ acting on $\mathbf{B}$. Further suppose that the α index set is the same in each case, so that it is sensible to construct the channel

$$\sum_\alpha \mathscr{S}_\alpha \otimes \mathscr{S}'_\alpha \tag{199}$$

acting on states $\rho_{\mathbf{AB}}$ on $\mathbf{A} \otimes \mathbf{B}$. We depict (199) acting on $\rho_{\mathbf{AB}}$ by the diagram

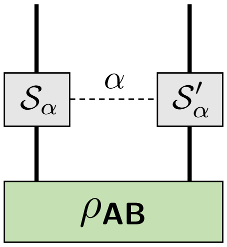

$$= \quad \sum_\alpha (\mathcal{S}_\alpha \otimes \mathcal{S}'_\alpha)[\rho_{\mathbf{AB}}] \tag{200}$$

where the dotted line with the α above denotes a sum over α.

Supplementary Note B: QUALM example: verification of quantum computation

Here we give another example of how to present a familiar experimental task in the QUALM framework. As this example is more advanced and requires some non-negligible background, we delayed it until now. An underlying assumption of quantum computing is that general quantum computations require exponential time to simulate on a classical computer. This has a very interesting implication, namely that given a quantum system claimed to perform a certain quantum computational task, it is not clear how to verify that the output is actually correct. Sometimes one can check the end result easily (e.g., in Shor's factoring algorithm, checking that the integer factors are correct can be achieved by multiplying those factors); however, for the output of a general quantum algorithm, this is not the case. The question is how to perform such a verification, despite the fact that simulating the system under investigation is assumed to take exponential time, and the correctness of the output cannot be directly checked (without having at hand a quantum computer known to work properly).

In the works of[3,4,16,37] a protocol to perform such a verification was provided, by an *almost* classical verifier. That is, the verifier has access to polynomial classical computing power, and also to a very small quantum computer composed of one or at most a few qubits which are fully controlled (i.e., whose behavior is fully understood).

Let us recall in more detail the modus operandi of such an *almost* classical verification protocol[4]. A 'verifier', who has the ability to perform quantum computation on only $\mathscr{O}(1)$ qubits (and otherwise can only perform classical computation),

is interacting with an agent called the 'prover' which may have a quantum computer. The verifier would like the prover to evaluate a chosen quantum circuit U on a chosen computational basis state $|\psi_0\rangle$, and provide him the correct value of a specific output bit b; but the verifier does not trust the prover, and so he wants to be sure that the prover is not cheating. The verifier may interact with the prover via an elaborate protocol of communication. We then require that a successful verification protocol satisfies the following criteria:

- If the prover does indeed have a quantum computer and is honest, then the output of the verification protocol is the correct output bit b with probability $\geq 1-\varepsilon$, for ε small.
- For any prover, the probability of the verification protocol outputting the *incorrect* bit $\bar{b}$ is $< \varepsilon$ (meaning that with probability $\geq 1-\varepsilon$ the output is either the correct bit b, or REJECT).

Now we formalize the above into a quantum task. The idea is to have a lab oracle $\mathsf{LO}_{\text{honestQC}}$ which formalizes the action of a quantum computer. We think of a quantum computer as a hybrid quantum-classical entity: it applies quantum gates on its quantum register depending on classical inputs which it is given, which classically describe the quantum gates it needs to apply.

To this end, we let $|\mathbf{N}| = n$; this will consist of the quantum register of the quantum computer. We let the lab subsystem be $\mathbf{L} \simeq \mathbf{L}_1 \otimes \mathbf{L}_2$ where $|\mathbf{L}_2| = \mathscr{O}(1)$ and $|\mathbf{L}_1| = \mathscr{O}(\text{poly}(n))$. $\mathbf{L}_2$ will correspond to the few quantum qubits which the verifier may control, and $\mathbf{L}_1$ will correspond to the register which serves as a classical communication channel between the verifier and the prover. We also set $|\mathbf{W}| = \mathscr{O}(\text{poly}(n))$ – this is the classical working register of the verifier.

We can now define a lab oracle $\mathsf{LO}_{\text{honestQC}} = (\mathscr{E}_{\mathbf{NL},\text{honest}}, |0\rangle\langle 0|^{\otimes n})$. This lab oracle will play the role of an honest prover with a quantum computer. The register $\mathbf{L}_1$ will store the instructions that the verifier will send to the prover about the quantum gates to be implemented; the honest prover lab oracle superoperator examines the $\mathbf{L}_1$ register and *correctly and honestly* modifies the set of qubits in $\mathbf{N} \otimes \mathbf{L}_2$ according to the instructions.

The corresponding quantum task is as follows. Let $\mathbf{S}_{\text{in}}$ be a $k = \mathscr{O}(\text{poly}(n))$ subsystem of $\mathbf{W}$. The input string in $\mathbf{S}_{\text{in}}$ encodes a classical description of a polynomial size quantum circuit U, as well as an input n qubit computational basis state $|\psi_0\rangle$ on which U is to act. Further, let $\mathbf{S}_{\text{out}}$ be a two qubit subset of $\mathbf{W}$; at the end of the protocol, this register will output one of three classical outputs: $0, 1$ and REJECT.

We restrict the admissible set of gates $\mathscr{G}$ so that only a few types of operations are allowed: first, we allow a universal quantum gate set on the "small quantum register" $\mathbf{L}_1$, where each of these gates is conditioned *classically* on one of the bits in $\mathbf{W}$. We also allow measurements in the computational basis on $\mathbf{L}_1$, whose classical output bit is written on a bit in $\mathbf{W}$ (this can be suitably expressed as a superoperator). Finally, we allow a universal *classical* gate set on $\mathbf{L}_2 \otimes \mathbf{W}$.

If $x_{|\psi_0\rangle,U}$ is a k-bit classical description of $|\psi_0\rangle$ and U, and $b = b(|\psi_0\rangle, U)$ is the desired correct output bit, then f is a probabilistic function (see Remark 2) defined by:

$$f : \mathscr{LO}(\mathbf{N},\mathbf{L}) \times \{0,1\}^k \longrightarrow \mathscr{D}(\{0,1,\mathsf{REJECT}\}) \tag{201}$$

where

$$f(\mathsf{LO}, x_{|\psi_0\rangle,U}) = \begin{cases} b(|\psi_0\rangle, U) & \text{with probability } \geq 1-\varepsilon \text{ if } \mathsf{LO} = \mathsf{LO}_{\text{honestQC}} \\ \bar{b} = 1 - b(|\psi_0\rangle, U) & \text{with probability } < \varepsilon \text{ for any } \mathsf{LO}\,. \end{cases} \tag{202}$$

Interestingly, this task can be achieved by a QUALM of polynomial QUALM complexity; this is the essence of the verification protocols of[3, 4, 16, 37]. It is a major open question whether this is possible if we require that all of $\mathbf{L}$ is classical.

Supplementary Note C: Review of unitary, orthogonal, and symplectic matrix integrals

Here we review various facts on Haar unitary, orthogonal, and symplectic matrix integrals, as well as prove some useful lemmas. Our review will be largely based on[24, 48, 73].

Supplementary Note C.1: Unitary matrix integrals

Here we review some facts about Haar unitary integrals, and the associated Weingarten functions. Consider the integral

$$\int_{U(D)} dU\, U_{i_1,j_1} U_{i_2,j_2} \cdots U_{i_k,j_k} \overline{U_{i'_1,j'_1} U_{i'_2,j'_2} \cdots U_{i'_k,j'_k}} = \sum_{\sigma,\tau \in S_k} \delta_{\sigma(I),I'}\, \delta_{\tau(J),J'}\, \mathrm{Wg}^U(\sigma\tau^{-1},D) \tag{203}$$

where $I = (i_1,...,i_k)$ and similarly for I', J, J'. Above, the bars mean complex conjugation, so that $\overline{U_{ij}} = U^\dagger_{ji}$. We have additionally used the multi-index notation

$$\delta_{\sigma(I),I'} := \delta_{i_{\sigma(1)},i'_1}\, \delta_{i_{\sigma(2)},i'_2} \cdots \delta_{i_{\sigma(k)},i'_k}\,. \tag{204}$$

To compress notation further, let $U^{\otimes k}_{IJ} := U_{i_1,j_1} U_{i_2,j_2} \cdots U_{i_k,j_k}$. Then we have, more compactly,

$$\int_{U(D)} dU\, U^{\otimes k}_{IJ} U^{\dagger \otimes k}_{KL} = \sum_{\sigma,\tau \in S_k} \delta_{\sigma(I),L}\, \delta_{\tau(J),K}\, \mathrm{Wg}^U(\sigma\tau^{-1},D)\,, \tag{205}$$

and accordingly

$$\sum_{I,J,K,L} \int_{U(D)} dU\, U^{\otimes k}_{IJ} A_{JK}\, U^{\dagger\otimes k}_{KL} B_{LI} = \sum_{I,J} \sum_{\sigma,\tau\in S_k} A_{J,\tau(J)}\, B_{\sigma(I),I}\, \mathrm{Wg}^U(\sigma\tau^{-1},D) \tag{206}$$

$$= \sum_{\sigma,\tau\in S_k} \mathrm{tr}(\tau^{-1}A)\, \mathrm{tr}(\sigma B)\, \mathrm{Wg}^U(\sigma\tau^{-1},D) \tag{207}$$

where $A, B \in \mathscr{B}(\mathsf{H}^{\otimes k})$. Note that $\mathrm{Wg}^U(\sigma,D)$ only depends on the conjugacy class of σ, i.e. $\mathrm{Wg}^U(\tau\sigma\tau^{-1},D) = \mathrm{Wg}^U(\sigma,D)$ for any $\tau \in S_k$. As such, $\mathrm{Wg}^U(\sigma,D) = \mathrm{Wg}^U(\sigma^{-1},D)$ and $\mathrm{Wg}^U(\sigma\tau^{-1},D) = \mathrm{Wg}^U(\tau^{-1}\sigma,D)$, for instance.

Two useful special cases of Supplementary Equation (205) are

$$\int_{U(D)} dU\, U_{i_1 j_2} U^\dagger_{j_2 i_2} = \frac{1}{D}\, \delta_{i_1,i_2} \delta_{j_1,j_2} \tag{208}$$

$$\int_{U(D)} dU\, U_{i_1 j_1} U_{i_2 j_2} U^\dagger_{j_3 i_3} U^\dagger_{j_4 i_4} = \frac{1}{D^2-1}\left(\delta_{i_1,i_3}\delta_{i_2,i_4}\delta_{j_1,j_3}\delta_{j_2,j_4} + \delta_{i_1,i_4}\delta_{i_2,i_3}\delta_{j_1,j_4}\delta_{j_2,j_3}\right) - \frac{1}{D(D^2-1)}\left(\delta_{i_1,i_3}\delta_{i_2,i_4}\delta_{j_1,j_4}\delta_{j_2,j_3} + \delta_{i_1,i_4}\delta_{i_2,i_3}\delta_{j_1,j_3}\delta_{j_2,j_4}\right)\,. \tag{209}$$

The Weingarten function Wg^U can be expressed as an inverse of a more simple matrix. Let

$$G^U(\sigma\tau^{-1},D) := \mathrm{tr}(\sigma\tau^{-1}) = D^{\#(\sigma\tau^{-1})} \tag{210}$$

where $\#(\sigma\tau^{-1})$ is the number of cycles of the permutation $\sigma\tau^{-1}$. We often also write

$$k - |\sigma\tau^{-1}| = \#(\sigma\tau^{-1})\,, \tag{211}$$

where $|\sigma\tau^{-1}|$ can be regarded as a 'distance' from $\sigma\tau^{-1}$ to the identity permutation. We have the identity

$$\sum_\tau \mathrm{Wg}^U(\sigma^{-1}\tau,D)\, G^U(\tau^{-1}\pi,D) = \delta_{\sigma,\pi} \tag{212}$$

and so G^U and Wg^U are inverses as $k! \times k!$ matrices. (Note that they are symmetric matrices.) Perhaps a more illuminating notation is $\mathrm{Wg}^U_D(\sigma,\tau) := \mathrm{Wg}^U(\sigma^{-1}\tau,D)$ and $G^U_D(\tau,\pi) := G^U_D(\tau^{-1}\pi,D)$ so that

$$\sum_\tau \mathrm{Wg}^U_D(\sigma,\tau)\, G^U_D(\tau,\pi) = \delta_{\sigma,\pi}\,. \tag{213}$$

We also recall the useful bound:

Theorem 3.2 of[24]. For any $\sigma \in S_k$ and $D > \sqrt{6}k^{7/4}$,

$$\frac{1}{1-\frac{k-1}{D^2}} \leq \frac{(-1)^{|\sigma|}D^{k+|\sigma|}\mathrm{Wg}^U(\sigma,D)}{\prod_i \frac{(2\ell_i-2)!}{(\ell_i-1)!\ell_i!}} \leq \frac{1}{1-\frac{6k^{7/2}}{D^2}} \tag{214}$$

where the left-hand side inequality is valid for any $D \geq k$. Note that $\sigma \in S_k$ has cycle type $(\ell_1, \ell_2, ...)$.

Next, we present two Lemmas, which we have made use of in the text:

Lemma 5. $|\mathrm{Wg}^U(\mathbb{1},D) - D^{-k}| \leq O(k^{7/2}D^{-(k+2)})$.

Proof. Follows directly from Supplementary Equation (214). □

Lemma 6. $\sum_{\tau \in S_k} |\mathrm{Wg}^U(\tau,D)| = \frac{(D-k)!}{D!}$.

Proof. We have $|\mathrm{Wg}^U(\tau,D)| = (-1)^{|\tau|}\mathrm{Wg}^U(\tau,D)$, and so

$$\sum_{\tau \in S_k} |\mathrm{Wg}^U(\tau,D)| = \sum_{\tau \in S_k} (-1)^{|\tau|}\mathrm{Wg}^U(\tau,D)\,. \tag{215}$$

Consider the matrix

$$F_{\sigma\tau} = |\mathrm{Wg}^U_D(\sigma,\tau)| = \mathrm{Wg}^U(\sigma^{-1}\tau,D)(-1)^{|\sigma^{-1}\tau|} = \mathrm{Wg}^U(\sigma^{-1}\tau,D)(-1)^{|\sigma|}(-1)^{|\tau|}\,. \tag{216}$$

Letting $P_{\sigma\tau} = \delta_{\sigma\tau}(-1)^{|\sigma|}$, we have

$$F = P\mathrm{Wg}^U_D P\,. \tag{217}$$

Let $\vec{1}$ be the vector of all ones. Since $F_{\sigma\tau}$ is only a function of $\sigma^{-1}\tau$, $\vec{1}$ is always an eigenvector of F:

$$F \cdot \vec{1} = \left(\sum_\tau F_\tau\right)\vec{1}\,. \tag{218}$$

The eigenvalue $(\sum_\tau F_\tau)$ is exactly the quantity we want to compute. Note that $\vec{1}$ is likewise an eigenvector of $F^{-1} = P(\mathrm{Wg}^U_D)^{-1}P = PG^U P$ with eigenvalue $(\sum_\tau F_\tau)^{-1}$. It follows that

$$\left(\sum_\tau F_\tau\right)^{-1} = \sum_\tau (-1)^{|\tau|}G^U(\tau,D) = D^k \sum_\tau (-D)^{-|\tau|} = \sum_{j=1}^{k} s(k,j)\,D^j = \frac{D!}{(D-k)!} \tag{219}$$

where $s(k,j)$ are the Stirling numbers of the first kind, which count the number of permutations of length k with j cycles, times a factor of $(-1)^{k-j}$. Thus we obtain

$$\sum_\tau |\mathrm{Wg}^U(\tau,D)| = \frac{(D-k)!}{D!} \tag{220}$$

as desired. □

Supplementary Note C.2: Orthogonal matrix integrals

Before delving into the details of orthogonal matrix integrals, it is first important to understand pair partitions on $\{1,2,...,2k\}$, namely partitions into k sets of size 2. The total number of such partitions is $\frac{(2k)!}{2^k k!} = (2k-1)!!$. There are two useful ways to think about this: the first is the set of pair partitions $P_2(2k)$. A pair partition $\mathfrak{m} \in P_2(2k)$ can be expressed as

$$\mathfrak{m} = \{\mathfrak{m}(1),\mathfrak{m}(2)\}\{\mathfrak{m}(3),\mathfrak{m}(4)\}\cdots\{\mathfrak{m}(2k-1),\mathfrak{m}(2k)\} \tag{221}$$

where we pick the ordering convention $\mathfrak{m}(2i-1) < \mathfrak{m}(2i)$ for $1 \le i \le k$ and $\mathfrak{m}(1) < \mathfrak{m}(3) < \cdots < \mathfrak{m}(2k-1)$. Alternatively, we can think of a pair partition as a member of the group M_{2k} which we treat as a subgroup of S_{2k} as follows. We say that $\sigma \in M_{2k}$ if $\sigma(2i-1) < \sigma(2i)$ for $1 \le i \le k$ and $\sigma(1) < \sigma(3) < \cdots < \sigma(2k-1)$. There is a natural group action of M_{2k} on $P_2(2k)$ in the obvious way:

$$\sigma \cdot \mathfrak{m} = \{\sigma(\mathfrak{m}(1)), \sigma(\mathfrak{m}(2))\}\{\sigma(\mathfrak{m}(3)), \sigma(\mathfrak{m}(4))\} \cdots \{\sigma(\mathfrak{m}(2k-1)), \sigma(\mathfrak{m}(2k))\}. \tag{222}$$

We can map between $P_2(2k)$ and M_{2k} in a canonical manner, namely $\mathfrak{m} \mapsto \sigma_{\mathfrak{m}}$ where $\sigma_{\mathfrak{m}}(i) = \mathfrak{m}(i)$.

Now we explain what cycles and cycle types mean in the context of pairings. These are often called the "coset type" of a pairing. We begin by explaining coset types in terms of $P_2(2k)$, and then afterwards give a different (albeit equivalent) construction of coset types in terms of M_{2k}. In the $P_2(2k)$ context, let $\mathfrak{m} \in P_2(2k)$ be a pairing. We define the function

$$f_{\mathfrak{m}}(i) = \begin{cases} \mathfrak{m}(2j) & \text{if } i = \mathfrak{m}(2j-1) \\ \mathfrak{m}(2j-1) & \text{if } i = \mathfrak{m}(2j) \end{cases}. \tag{223}$$

In words, $f_{\mathfrak{m}}(i)$ maps i to the integer it is paired with under $\mathfrak{m}$. Let $\mathfrak{e}$ be the identity pairing $\{1,2\}\{3,4\}\cdots\{2k-1,2k\}$. Now we construct a sequence starting with 1:

$$(1, f_{\mathfrak{m}}(1), f_{\mathfrak{e}} \circ f_{\mathfrak{m}}(1), f_{\mathfrak{m}} \circ f_{\mathfrak{e}} \circ f_{\mathfrak{m}}(1), f_{\mathfrak{e}} \circ f_{\mathfrak{m}} \circ f_{\mathfrak{e}} \circ f_{\mathfrak{m}}(1), ..., 1) \tag{224}$$

where the sequence terminates the first time we reach 1 again. If we treat the above $(\cdots)$ notation as defined cyclically, then we can omit the two copies of 1. Let us do this. We call this new cyclic object

$$B_1 = (1, f_{\mathfrak{m}}(1), f_{\mathfrak{e}} \circ f_{\mathfrak{m}}(1), f_{\mathfrak{m}} \circ f_{\mathfrak{e}} \circ f_{\mathfrak{m}}(1), f_{\mathfrak{e}} \circ f_{\mathfrak{m}} \circ f_{\mathfrak{e}} \circ f_{\mathfrak{m}}(1), ...) \tag{225}$$

with length b_1. If B_1 contains all of $\{1,2,...,2k\}$, then we are done. Otherwise, we find the smallest j in $\{1,2,...,2k\}$ that is not contained in B_1, and construct

$$B_2 = (j, f_{\mathfrak{m}}(j), f_{\mathfrak{e}} \circ f_{\mathfrak{m}}(j), f_{\mathfrak{m}} \circ f_{\mathfrak{e}} \circ f_{\mathfrak{m}}(j), f_{\mathfrak{e}} \circ f_{\mathfrak{m}} \circ f_{\mathfrak{e}} \circ f_{\mathfrak{m}}(j), ...) \tag{226}$$

which again terminates so that it is cyclic with the smallest possible period. If B_1 and B_2 do not jointly contain all $\{1,2,...,2k\}$, then we proceed by constructing a B_3, and so on. At the end of this procedure, we have a partition of $\{1,2,...,2k\}$ into $B_1 B_2 \cdots$ such that $b_1, b_2, ...$ are all even. Then the cycle type or coset type of $\mathfrak{m}$ in M_{2k} is

$$(\mu_1, \mu_2, ...) = (b_1/2, b_2/2, ...). \tag{227}$$

which we typically (re)order from largest to smallest.

In terms of the M_{2k}, we can consider the following algorithm: Let $\sigma \in M_{2k}$, and let $C_1 C_2 \cdots$ be its cycle decomposition (including cycles of length one) as an element of S_{2k}. The cycles have lengths $\ell_1, \ell_2, ...$ respectively. To construct the cycle type of σ as an element of M_{2k}, we consider all pairs of integers $2i-1, 2i$. If some such pair has the property that $2i-1$ and $2i$ belong to distinct cycles, then we "combine" those two cycles (simply by considering a set containing those two cycles). We do this for all pairs $2i-1, 2i$ when necessary (some pairs will already belong to the same cycle) and end with M_{2k}-cycles $\widetilde{C}_1 \widetilde{C}_2 \cdots$ where $\widetilde{C}_i = \{C_{i,1}, C_{i,2}, ...\}$ and by construction $\widetilde{C}_i \cap \widetilde{C}_j = \emptyset$ for $i \neq j$. The lengths of these M_{2k}-cycles are $\widetilde{\ell}_1, \widetilde{\ell}_2, ...$ where $\widetilde{\ell}_i := \ell_{i,1} + \ell_{i,2} + \cdots$. Note that all the $\widetilde{\ell}_i$'s are by construction even, and so we say that the M_{2k}-cycle type of σ is

$$(\mu_1, \mu_2, ...) = (\widetilde{\ell}_1/2, \widetilde{\ell}_2/2, ...) \tag{228}$$

which similar to above, we typically (re)order from largest to smallest. Said more simply, we take the cycle type of $\sigma \in S_{2k}$ and "connect" cycles so that all pairs $2i-1, 2i$ lie within the same "connected" cycle. These connected cycles necessarily have even length, and so we can divide these lengths by two. It can be checked that $\mathfrak{m}$ and its counterpart $\sigma_{\mathfrak{m}}$ have the same cycle types when we process each with its appropriate algorithm.

With our knowledge of pair partitions at hand, we can now delve into the Weingarten calculus for orthogonal matrix integrals. Let $O_{IJ} = O_{i_1,j_1} O_{i_2,j_2} \cdots O_{i_{2k},j_{2k}}$ where $I = (i_1, i_2, ..., i_{2k})$ and similarly for J. Note that in contrast to our notation before, I and J are multi-indices with length $2k$ (instead of k). We will adjust this back to k shortly. At present, we have

$$\int_{O(D)} dO\, O_{IJ} = \sum_{\mathfrak{m},\mathfrak{n} \in P_2(2k)} \Delta_{\mathfrak{m}}(I)\, \Delta_{\mathfrak{n}}(J)\, \mathrm{Wg}_D^O(\mathfrak{m},\mathfrak{n}) \tag{229}$$

where

$$[\Delta_{\mathfrak{m}}]_{i_1 i_3 \ldots i_{2k-1}}^{i_2 i_4 \ldots i_{2k}} = \prod_{s=1}^{k} \delta_{i_{\mathfrak{m}(2s-1)}, i_{\mathfrak{m}(2s)}} . \tag{230}$$

Since there is a canonical map from $P_2(2k)$ to M_{2k} taking $\mathfrak{m} \mapsto \sigma_{\mathfrak{m}}$, we can also write

$$\mathrm{Wg}_D^O(\mathfrak{m},\mathfrak{n}) = \mathrm{Wg}_D^O(\sigma_{\mathfrak{m}}, \sigma_{\mathfrak{n}}) = \mathrm{Wg}^O(\sigma_{\mathfrak{m}}^{-1}\sigma_{\mathfrak{n}}, D) \tag{231}$$

similar to the unitary case from before.

For our purposes, it will be useful to reorganize Supplementary Equation (229) slightly. Let $I = (i_1, ..., i_k)$, $J = (j_1, ..., j_k)$, $I' = (i'_1, ..., i'_k)$ and $J' = (j'_1, ..., j'_k)$. Note that now, each of I, J, I', J' is a multi-index of length k. We denote by II' the combined multi-index $II' = (i_1, i'_1 ..., i_k, i'_k)$ and similarly for JJ'. Then we have

$$\int_{O(D)} dO\, O_{IJ}\, O^T_{J'I'} = \sum_{\mathfrak{m},\mathfrak{n} \in P_2(2k)} \Delta_{\mathfrak{m}}(II')\, \Delta_{\mathfrak{n}}(JJ')\, \mathrm{Wg}_D^O(\mathfrak{m},\mathfrak{n}) \tag{232}$$

and accordingly

$$\sum_{I,J,I',J'} \int_{O(D)} dO\, O_{IJ}\, A_{JJ'}\, O^T_{J'I'}\, B_{I'I} = \sum_{\mathfrak{m},\mathfrak{n} \in P_2(2k)} \mathrm{tr}(\Delta_{\mathfrak{n}} A)\, \mathrm{tr}(\Delta_{\mathfrak{m}} B)\, \mathrm{Wg}_D^O(\mathfrak{m},\mathfrak{n}) . \tag{233}$$

Note that $\mathrm{tr}(\Delta_{\mathfrak{n}} A)$ computes the pairwise contraction the $2k$ of indices of $A_{JJ'}$ corresponding to $\mathfrak{n}$, and $\mathrm{tr}(\Delta_{\mathfrak{m}} B)$ is defined similarly.

Using Supplementary Equation (232), the first two (nontrivial) moments of the orthogonal ensemble are

$$\int_{O(D)} dO\, O_{i_1 j_2} O^T_{j_2 i_2} = \frac{1}{D} \delta_{i_1,i_2} \delta_{j_1,j_2} \tag{234}$$

$$\begin{aligned} &\int_{O(D)} dO\, O_{i_1 j_1} O_{i_2 j_2} O^T_{j_3 i_3} O^T_{j_4 i_4} \qquad (235) \\ &= \frac{D+1}{D(D-1)(D+2)} \left(\delta_{i_1,i_2}\delta_{i_3,i_4}\delta_{j_1,j_2}\delta_{j_3,j_4} + \delta_{i_1,i_3}\delta_{i_2,i_4}\delta_{j_1,j_3}\delta_{j_2,j_4} + \delta_{i_1,i_4}\delta_{i_2,i_3}\delta_{j_1,j_4}\delta_{j_2,j_3} \right) \\ &\quad - \frac{1}{D(D-1)(D+2)} \left(\delta_{i_1,i_3}\delta_{i_2,i_4}\delta_{j_1,j_4}\delta_{j_2,j_3} + \delta_{i_1,i_2}\delta_{i_3,i_4}\delta_{j_1,j_4}\delta_{j_2,j_3} + \delta_{i_1,i_4}\delta_{i_2,i_3}\delta_{j_1,j_3}\delta_{j_2,j_4} \right. \\ &\quad \left. + \delta_{i_1,i_2}\delta_{i_3,i_4}\delta_{j_1,j_3}\delta_{j_2,j_4} + \delta_{i_1,i_4}\delta_{i_2,i_3}\delta_{j_1,j_2}\delta_{j_3,j_4} + \delta_{i_1,i_3}\delta_{i_2,i_4}\delta_{j_1,j_2}\delta_{j_3,j_4} \right) . \end{aligned}$$

Similar to the unitary case, we can express Wg^O as the inverse of a simpler matrix. Let

$$G_D^O(\sigma_{\mathfrak{m}}, \sigma_{\mathfrak{n}}) := D^{\#_O(\sigma_{\mathfrak{m}}^{-1}\sigma_{\mathfrak{n}})} \tag{236}$$

where $\#_O(\sigma_{\mathfrak{m}}^{-1}\sigma_{\mathfrak{n}})$ is the number of M_{2k}-cycles of $\sigma_{\mathfrak{m}}^{-1}\sigma_{\mathfrak{n}}$. Note that G_D^O is a $(2k-1)!! \times (2k-1)!!$ matrix. We often also write

$$k - |\sigma_{\mathfrak{m}}^{-1}\sigma_{\mathfrak{n}}|_O = \#_O(\sigma_{\mathfrak{m}}^{-1}\sigma_{\mathfrak{n}}) , \tag{237}$$

where $|\sigma_{\mathfrak{m}}^{-1}\sigma_{\mathfrak{n}}|_O$ can be regarded as a 'distance' from $\sigma_{\mathfrak{m}}^{-1}\sigma_{\mathfrak{n}}$ to the identity pairing. We have the identity

$$\sum_{\mathfrak{n}\in P_2(2k)} \mathrm{Wg}_D^O(\sigma_{\mathfrak{m}},\sigma_{\mathfrak{n}})\, G_D^O(\sigma_{\mathfrak{n}},\sigma_{\mathfrak{p}}) = \delta_{\mathfrak{m},\mathfrak{p}}\,, \tag{238}$$

and so evidently Wg^O and G^O are inverses.

Similar to the unitary case, we have the following useful bound:

Theorem 4.11 of[24]. For any $\sigma_{\mathfrak{m}} \in M_{2k}$ and $D > 12k^{7/2}$,

$$\frac{1-\frac{24k^{7/2}}{D}}{1-\frac{144k^7}{D^2}} \le \frac{(-1)^{|\sigma_{\mathfrak{m}}|_O} D^{k+|\sigma_{\mathfrak{m}}|_O}\mathrm{Wg}^O(\sigma_{\mathfrak{m}},D)}{\prod_i \frac{(2\mu_i-2)!}{(\mu_i-1)!\mu_i!}} \le \frac{1}{1-\frac{144k^7}{D^2}} \tag{239}$$

where $\sigma_{\mathfrak{m}}$ has M_{2k}-cycle type $(\mu_1,\mu_2,\ldots)$.

This bound is slightly weaker than Supplementary Equation (214) in the unitary case, but it will suffice for our purposes.

We will now present two Lemmas analogous to the unitary case:

Lemma 7. $|\mathrm{Wg}^O(\sigma_{\mathfrak{e}},D) - D^{-k}| \le O(k^7 D^{-(k+2)})$.

Proof. Follows directly from Supplementary Equation (239). □

Lemma 8. $\sum_{\mathfrak{m}\in P_2(2k)} |\mathrm{Wg}^O(\sigma_{\mathfrak{m}},D)| = \frac{(D-2k)!!}{D!!}$.

Proof. We have $|\mathrm{Wg}^O(\tau_{\mathfrak{m}},D)| = (-1)^{|\tau_{\mathfrak{m}}|_O}\mathrm{Wg}^O(\tau_{\mathfrak{m}},D)$, and so

$$\sum_{\mathfrak{m}\in P_2(2k)} |\mathrm{Wg}^O(\tau_{\mathfrak{m}},D)| = \sum_{\mathfrak{m}\in P_2(2k)} (-1)^{|\tau_{\mathfrak{m}}|_O}\mathrm{Wg}^O(\tau_{\mathfrak{m}},D)\,. \tag{240}$$

Let us consider the matrix

$$F_{\mathfrak{m}\mathfrak{n}} = |\mathrm{Wg}_D^O(\mathfrak{m},\mathfrak{n})| = \mathrm{Wg}^O(\sigma_{\mathfrak{m}}^{-1}\sigma_{\mathfrak{n}},D)(-1)^{|\sigma_{\mathfrak{m}}^{-1}\sigma_{\mathfrak{n}}|_O} = \mathrm{Wg}^O(\sigma_{\mathfrak{m}}^{-1}\sigma_{\mathfrak{n}},D)(-1)^{|\sigma_{\mathfrak{m}}|_O}(-1)^{|\sigma_{\mathfrak{n}}|_O}\,. \tag{241}$$

Defining $P_{\mathfrak{m}\mathfrak{n}} = \delta_{\mathfrak{m}\mathfrak{n}}(-1)^{|\sigma_{\mathfrak{m}}|_O}$, we automatically have the relation

$$F = P\mathrm{Wg}_D^O P\,. \tag{242}$$

As before, we let $\vec{1}$ be the vector of all ones. Noting that $F_{\mathfrak{m}\mathfrak{n}}$ is only a function of $\sigma_{\mathfrak{m}}^{-1}\sigma_{\mathfrak{n}}$, the vector $\vec{1}$ is always an eigenvector of F:

$$F\cdot\vec{1} = \left(\sum_{\mathfrak{m}} F_{\sigma_{\mathfrak{m}}}\right)\vec{1}\,. \tag{243}$$

We would like to compute the eigenvalue $(\sum_{\mathfrak{m}} F_{\sigma_{\mathfrak{m}}})$. Since $\vec{1}$ is also an eigenvector of $F^{-1} = P(\mathrm{Wg}_D^O)^{-1}P = PG^OP$ with eigenvalue $(\sum_{\mathfrak{m}} F_{\sigma_{\mathfrak{m}}})^{-1}$, we have

$$\left(\sum_{\mathfrak{m}} F_{\sigma_{\mathfrak{m}}}\right)^{-1} = \sum_{\mathfrak{m}} (-1)^{|\sigma_{\mathfrak{m}}|_O} G^O(\sigma_{\mathfrak{m}},D) = \sum_{j=1}^{k} \widetilde{s}(k,j)\,D^j = \frac{D!!}{(D-2k)!!} \tag{244}$$

where $\widetilde{s}(k,j)$ counts the number of $2k$-pairings with j M_{2k}-cycles, times a factor of $(-1)^{k-j}$. We note that $\widetilde{s}(k,j) = \frac{2^k}{2^j}\,s(k,j)$. Finally, we find

$$\sum_{\mathfrak{m}\in P_2(2k)} |\mathrm{Wg}^O(\sigma_{\mathfrak{m}},D)| = \frac{(D-2k)!!}{D!!} \tag{245}$$

which is the claimed result. □

Supplementary Note C.3: Symplectic matrix integrals

Finally, we treat the symplectic case. This is rather similar to the orthogonal case, with one additional ingredient: the $D \times D$ matrix

$$\mathbf{J} = \begin{bmatrix} \mathbf{0}_{D/2} & \mathbb{1}_{D/2} \\ -\mathbb{1}_{D/2} & \mathbf{0}_{D/2} \end{bmatrix}, \tag{246}$$

where we note that $\mathbf{J}^T = -\mathbf{J}$ and we take D to be even. Here, $\mathbf{0}_{D/2}$ is the $D/2 \times D/2$ matrix of all zeros.

To avoid extensive duplication of the previous sections, we cut to the bare essentials. As before, let $I = (i_1, ..., i_k)$, $J = (j_1, ..., j_k)$, $I' = (i'_k, ..., i'_k)$ and $J' = (j'_1, ..., j'_k)$. Then we have

$$\int_{\mathrm{Sp}(D/2)} dS\, S_{IJ}\, S^T_{J'I'} = \sum_{\mathfrak{m},\mathfrak{n} \in P_2(2k)} \Delta'_{\mathfrak{m}}(II')\, \Delta'_{\mathfrak{n}}(JJ')\, \mathrm{Wg}^{\mathrm{Sp}}_{D/2}(\mathfrak{m},\mathfrak{n}) \tag{247}$$

where

$$[\Delta'_{\mathfrak{m}}]^{i_2 i_4 \dots i_{2k}}_{i_1 i_3 \dots i_{2k-1}} = \prod_{s=1}^{k} \mathbf{J}_{i_{\mathfrak{m}(2s-1)}, i_{\mathfrak{m}(2s)}}\,. \tag{248}$$

Letting $\mathbf{J}_{MN} = \mathbf{J}_{m_1,n_1} \mathbf{J}_{m_2,n_2} \cdots \mathbf{J}_{m_k,n_k}$, we have

$$\begin{aligned} \sum_{I,J,K,L,M,N} \int_{\mathrm{Sp}(D/2)} dS\, S_{IJ}\, A_{JK}\, \mathbf{J}_{KL}\, S^T_{LM}\, \mathbf{J}^T_{MN}\, B_{NI} \\ = \sum_{\mathfrak{m},\mathfrak{n} \in P_2(2k)} \mathrm{tr}(\Delta'_{\mathfrak{n}} A \mathbf{J}^{\otimes k})\, \mathrm{tr}(\Delta'_{\mathfrak{m}} \mathbf{J}^{T\otimes k}\, B)\, \mathrm{Wg}^{\mathrm{Sp}}_{D/2}(\mathfrak{m},\mathfrak{n})\,. \end{aligned} \tag{249}$$

Here $\mathrm{tr}(\Delta'_{\mathfrak{n}} A \mathbf{J}^{\otimes k})$ computes the pairwise contraction the $2k$ of indices of $A\mathbf{J}^{\otimes k}$ corresponding to $\mathfrak{n}$, with an additional $\mathbf{J}^{\otimes k}$ sandwiched in the middle. The quantity $\mathrm{tr}(\Delta'_{\mathfrak{m}} \mathbf{J}^{T\otimes k} B)$ is defined similarly.

Using Supplementary Equation (247) to compute the first two (nontrivial) moments of the symplectic ensemble, we find

$$\int_{\mathrm{Sp}(D/2)} dS\, S_{i_1 j_1} S^\dagger_{j_2 i_2} = \frac{1}{D} \delta_{i_1,i_2} \delta_{j_1,j_2} \tag{250}$$

$$\begin{aligned} &\int_{\mathrm{Sp}(D/2)} dS\, S_{i_1 j_1} S_{i_2 j_2} S^\dagger_{j_3 i_3} S^\dagger_{j_4 i_4} \\ &= \frac{D-1}{D(D+1)(D-2)} \left(\mathbf{J}_{i_1,i_2} \mathbf{J}_{i_3,i_4} \mathbf{J}_{j_1,j_2} \mathbf{J}_{j_3,j_4} + \delta_{i_1,i_3} \delta_{i_2,i_4} \delta_{j_1,j_3} \delta_{j_2,j_4} + \delta_{i_1,i_4} \delta_{i_2,i_3} \delta_{j_1,j_4} \delta_{j_2,j_3} \right) \\ &\quad - \frac{1}{D(D+1)(D-2)} \left(\delta_{i_1,i_3} \delta_{i_2,i_4} \delta_{j_1,j_4} \delta_{j_2,j_3} - \mathbf{J}_{i_1,i_2} \mathbf{J}_{i_3,i_4} \delta_{j_1,j_4} \delta_{j_2,j_3} + \delta_{i_1,i_4} \delta_{i_2,i_3} \delta_{j_1,j_3} \delta_{j_2,j_4} \right. \\ &\quad \left. + \mathbf{J}_{i_1,i_2} \mathbf{J}_{i_3,i_4} \delta_{j_1,j_3} \delta_{j_2,j_4} - \delta_{i_1,i_4} \delta_{i_2,i_3} \mathbf{J}_{j_1,j_2} \mathbf{J}_{j_3,j_4} + \delta_{i_1,i_3} \delta_{i_2,i_4} \mathbf{J}_{j_1,j_2} \mathbf{J}_{j_3,j_4} \right) \end{aligned} \tag{251}$$

where the expression for (251) comes from[28].

Similar to the unitary and orthogonal cases, we have the following useful bound:

Theorem 4.10 of[24]. For any $\sigma_{\mathfrak{m}} \in M_{2k}$ and $D > 6k^{7/2}$, we have

$$\frac{1}{1 - \frac{k-1}{(D/2)^2}} \le \frac{D^{k+|\sigma_{\mathfrak{m}}|_{\mathrm{Sp}}} |\mathrm{Wg}^{\mathrm{Sp}}(\sigma_{\mathfrak{m}}, D/2)|}{\prod_i \frac{(2\mu_i - 2)!}{(\mu_i - 1)!\mu_i!}} \le \frac{1}{1 - \frac{6k^{7/2}}{(D/2)^2}} \tag{252}$$

where $\sigma_{\mathfrak{m}}$ has M_{2k}-cycle type $(\mu_1, \mu_2, \dots)$ and $|\sigma_{\mathfrak{m}}|_{\mathrm{Sp}} = |\sigma_{\mathfrak{m}}|_O$.

We also have

$$\mathrm{Wg}^{\mathrm{Sp}}(\sigma_{\mathfrak{m}},D/2)=(-1)^{k}\varepsilon(\sigma_{\mathfrak{m}})\mathrm{Wg}^{O}(\sigma_{\mathfrak{m}},-D) \tag{253}$$

where $\varepsilon(\sigma_{\mathfrak{m}})$ is the signature of $\sigma_{\mathfrak{m}}$ as an element of S_{2k}.

We will now present two Lemmas analogous to the unitary and orthogonal cases:

Lemma 9. $|\mathrm{Wg}^{\mathrm{Sp}}(\sigma_{\mathfrak{e}},D/2)-D^{-k}|\leq\mathscr{O}(k^{7/2}D^{-(k+2)})$

Proof. This follows directly from Supplementary Equations (252) and (253). □

Lemma 10. $\sum_{\mathfrak{m}\in P_2(2k)}|\mathrm{Wg}^{\mathrm{Sp}}(\sigma_{\mathfrak{m}},D/2)|=\prod_{j=0}^{k-1}\frac{1}{D+2j}$.

Proof. We have $|\mathrm{Wg}^{\mathrm{Sp}}(\tau_{\mathfrak{m}},D/2)|=|\mathrm{Wg}^{O}(\tau_{\mathfrak{m}},-D)|$. Since for large enough D, $\mathrm{Wg}^{O}(\tau_{\mathfrak{m}},D/2)\sim C_{\mathfrak{m}}(-1)^{|\tau_{\mathfrak{m}}|_O}D^{-k-|\tau_{\mathfrak{m}}|_O}$ for $C_{\mathfrak{m}}>0$, it follows that $(-1)^{|\tau_{\mathfrak{m}}|_O}\mathrm{Wg}^{O}(\tau_{\mathfrak{m}},D/2)>0$ and so $|\mathrm{Wg}^{O}(\tau_{\mathfrak{m}},D/2)|=(-1)^{|\tau_{\mathfrak{m}}|_O}\mathrm{Wg}^{O}(\tau_{\mathfrak{m}},D/2)$. Similarly, $\mathrm{Wg}^{O}(\tau_{\mathfrak{m}},-D)\sim C_{\mathfrak{m}}(-1)^{|\tau_{\mathfrak{m}}|_O}(-D)^{-k-|\tau_{\mathfrak{m}}|_O}$ and so we find $(-1)^{|\tau_{\mathfrak{m}}|_O}\mathrm{Wg}^{O}(\tau_{\mathfrak{m}},-D)\sim C_{\mathfrak{m}}(-D)^{-k-|\tau_{\mathfrak{m}}|_O}$. To make both sides positive, we can multiply by $(-1)^{-k-|\tau_{\mathfrak{m}}|_O}$, and get

$$|\mathrm{Wg}^{\mathrm{Sp}}(\tau_{\mathfrak{m}},D/2)|=|\mathrm{Wg}^{O}(\tau_{\mathfrak{m}},-D)|=(-1)^{k}\,\mathrm{Wg}^{O}(\tau_{\mathfrak{m}},-D)\,. \tag{254}$$

Now consider

$$G^{O}_{-D}(\sigma_{\mathfrak{m}},\sigma_{\mathfrak{n}})=(-D)^{\#_O(\sigma_{\mathfrak{m}}^{-1}\sigma_{\mathfrak{n}})} \tag{255}$$

Similar to before, we have

$$\sum_{\mathfrak{n}\in P_2(2k)}\mathrm{Wg}^{O}_{-D}(\sigma_{\mathfrak{m}},\sigma_{\mathfrak{n}})\,G^{O}_{-D}(\sigma_{\mathfrak{n}},\sigma_{\mathfrak{p}})=\delta_{\mathfrak{m},\mathfrak{p}}\,. \tag{256}$$

Then similar to our previous proofs,

$$\mathrm{Wg}^{O}_{-D}\cdot\vec{1}=\left(\sum_{\mathfrak{m}\in P_2(2k)}\mathrm{Wg}^{O}(\tau_{\mathfrak{m}},-D)\right)\vec{1} \tag{257}$$

and thus

$$\left(\sum_{\mathfrak{m}}\mathrm{Wg}^{O}(\tau_{\mathfrak{m}},-D)\right)^{-1}=\sum_{\mathfrak{m}}G^{O}(\tau_{\mathfrak{m}},-D)=\sum_{\mathfrak{m}}(-D)^{\#_O(\tau_{\mathfrak{m}})}=\sum_{j=1}^{k}\widetilde{s}(k,j)\,(-D)^{j}\,. \tag{258}$$

This is clearly equivalent to

$$\prod_{j=0}^{k-1}(-D-2j)=(-1)^{k}\prod_{j=0}^{k-1}(D+2j)\,. \tag{259}$$

Finally, we have

$$\sum_{\mathfrak{m}\in P_2(2k)}|\mathrm{Wg}^{\mathrm{Sp}}(\tau_{\mathfrak{m}},D/2)|=(-1)^{k}\sum_{\mathfrak{m}\in P_2(2k)}\mathrm{Wg}^{O}(\tau_{\mathfrak{m}},-D)=\prod_{j=0}^{k-1}\frac{1}{D+2j} \tag{260}$$

as claimed. □

References

1. Aaronson, S. (2019). Shadow tomography of quantum states. *SIAM Journal on Computing*, 49(5), STOC18-368.

2. Aharonov, D., & Ben-Or, M. (2008). Fault-tolerant quantum computation with constant error rate. *SIAM Journal on Computing*.

3. Aharonov, D., Ben-Or, M., & Eban, E. (2010). *Proceedings of Innovations in Computer Science*.

4. Aharonov, D., Ben-Or, M., Eban, E., & Mahadev, U. (2017). Interactive proofs for quantum computations. *arXiv:1704.04487*.

5. Aharonov, D., Kitaev, A., & Nisan, N. (1998, May). Quantum circuits with mixed states. *In Proceedings of the Thirtieth Annual ACM Symposium on Theory of Computing* (pp. 20-30).

6. Araújo, M., Feix, A., Costa, F., & Brukner, Č. (2014). Quantum circuits cannot control unknown operations. *New Journal of Physics*, 16(9), 093026.

7. Arora, S., & Barak, B. (2009). Computational Complexity: A Modern Approach. *Cambridge University Press*.

8. Arrad, G., Vinkler, Y., Aharonov, D., & Retzker, A. (2014). Increasing sensing resolution with error correction. *Physical Review Letters*, 112(15), 150801.

9. Arunachalam, S., Grilo, A. B., & Sundaram, A. (2019). Quantum hardness of learning shallow classical circuits. *arXiv:1903.02840*.

10. Atia, Y., & Aharonov, D. (2017). Fast-forwarding of Hamiltonians and exponentially precise measurements. *Nature Communications*, 8(1), 1-9.

11. Bacon, D., Childs, A. M., & van Dam, W. (2005). Optimal measurements for the dihedral hidden subgroup problem. *quant-ph/0501044*.

12. Bar-Yossef, Z., Jayram, T. S., & Kerenidis, I. (2008). Exponential separation of quantum and classical one-way communication complexity. *SIAM Journal on Computing*, 38(1), 366-384.

13. Barenco, A., Bennett, C. H., Cleve, R., DiVincenzo, D. P., Margolus, N., Shor, P., ... & Weinfurter, H. (1995). Elementary gates for quantum computation. *Physical Review A*, 52(5), 3457.

14. Barz, S., Fitzsimons, J. F., Kashefi, E., & Walther, P. (2013). Experimental verification of quantum computation. *Nature Physics*, 9(11), 727-731.

15. Bernstein, E., & Vazirani, U. (1997). Quantum complexity theory. *SIAM Journal on Computing*, 26(5), 1411-1473.

16. Broadbent, A., Fitzsimons, J., & Kashefi, E. (2009, October). Universal blind quantum computation. In 2009 *50th Annual IEEE Symposium on Foundations of Computer Science* (pp. 517-526). IEEE.

17. Bubeck, S., Chen, S., & Li, J. (2020, November). Entanglement is necessary for optimal quantum property testing. In *2020 IEEE 61st Annual Symposium on Foundations of Computer Science (FOCS)* (pp. 692-703). IEEE.

18. Buhrman, H., Cleve, R., Watrous, J., & De Wolf, R. (2001). Quantum fingerprinting. *Physical Review Letters*, 87(16), 167902.

19. Chia, N. H., Chung, K. M., & Lai, C. Y. (2020, June). On the need for large quantum depth. In *Proceedings of the 52nd Annual ACM SIGACT Symposium on Theory of Computing* (pp. 902-915).

20. Childs, A. M., Harrow, A. W., & Wocjan, P. (2007, February). Weak Fourier-Schur sampling, the hidden subgroup problem, and the quantum collision problem. In *Annual Symposium on Theoretical Aspects of Computer Science* (pp. 598-609). Springer, Berlin, Heidelberg.

21. Chiribella, G., D'Ariano, G. M., & Perinotti, P. (2008). Quantum circuit architecture. *Physical Review Letters*, 101(6), 060401.

22. Chitambar, E., Leung, D., Mančinska, L., Ozols, M., & Winter, A. (2014). Everything you always wanted to know about LOCC (but were afraid to ask). *Communications in Mathematical Physics*, 328(1), 303-326.

23. Chung, K. M., & Lin, H. H. (2018). Sample Efficient Algorithms for Learning Quantum Channels in PAC Model and the Approximate State Discrimination Problem. *arXiv:1810.10938*.

24. Collins, B., & Matsumoto, S. (2017). Weingarten calculus via orthogonality relations: new applications. *arXiv:1701.04493*.

25. Cotler, J., Choi, S., Lukin, A., Gharibyan, H., Grover, T., Tai, M. E., ... & Greiner, M. (2019). Quantum Virtual Cooling. *Physical Review X*, 9(3), 031013.

26. Cotler, J., Han, X., Qi, X. L., & Yang, Z. (2019). Quantum Causal Influence. *Journal of High Energy Physics*, 2019(7), 42.

27. Cotler, J., Jian, C. M., Qi, X. L., & Wilczek, F. (2018). Superdensity operators for spacetime quantum mechanics. *Journal of High Energy Physics*, 2018(9), 93.

28. Cotler, J., & Hunter-Jones, N. (2019). Spectral decoupling in many-body quantum chaos. *arXiv:1911.02026*.

29. Cotler, J., & Wilczek, F. (2020). Quantum overlapping tomography. *Physical Review Letters*, 124(10), 100401.

30. Coudron, M., & Menda, S. (2020). Computations with greater quantum depth are strictly more powerful (relative to an oracle). In *Proceedings of the 52nd Annual ACM SIGACT Symposium on Theory of Computing* (pp. 889-901).

31. D'Ariano, G. M., Paris, M. G., & Sacchi, M. F. (2003). Quantum tomography. *Advances in Imaging and Electron Physics*, 128, 206-309.

32. Dong, D., & Petersen, I. R. (2010). Quantum control theory and applications: a survey. *IET Control Theory & Applications*, 4(12), 2651-2671.

33. Dong, Q., Nakayama, S., Soeda, A., & Murao, M. (2019). Controlled quantum operations and combs, and their applications to universal controllization of divisible unitary operations. *arXiv:1911.01645*.

34. Dür, W., Skotiniotis, M., Froewis, F., & Kraus, B. (2014). Improved quantum metrology using quantum error correction. *Physical Review Letters*, 112(8), 080801.

35. Ettinger, M., Høyer, P., & Knill, E. (2004). The quantum query complexity of the hidden subgroup problem is polynomial. *Information Processing Letters*, 91(1), 43-48.

36. Evans, T. J., Harper, R., & Flammia, S. T. (2019). Scalable Bayesian Hamiltonian learning. *arXiv:1912.07636*.

37. Fitzsimons, J. F., & Kashefi, E. (2017). Unconditionally verifiable blind quantum computation. *Physical Review A*, 96(1), 012303.

38. Fizeau, M. H. (1851). On the hypotheses relating to the luminous æther, and an experiment which appears to demonstrate that the motion of bodies alters the velociety with which light propagates itself in their interior. *The London, Edinburgh, and Dublin Philosophical Magazine and Journal of Science*, 2(14), 568-573.

39. Flammia, S. T., Gross, D., Liu, Y. K., & Eisert, J. (2012). Quantum tomography via compressed sensing: error bounds, sample complexity and efficient estimators. *New Journal of Physics*, 14(9), 095022.

40. Gavinsky, D., Kempe, J., Kerenidis, I., Raz, R., & de Wolf, R. (2007, June). Exponential separations for one-way quantum communication complexity, with applications to cryptography. In *Proceedings of the Thirty-ninth Annual ACM Symposium on Theory of Computing* (pp. 516-525).

41. Gavorová, Z., Seidel, M., & Touati, Y. (2020). Topological obstructions to implementing controlled unknown unitaries. *arXiv:2011.10031*.

42. Gharibian, S., Huang, Y., Landau, Z., & Shin, S. W. (2014). Quantum Hamiltonian Complexity. *arXiv:1401.3916.*

43. Gharibian, S., Piddock, S., & Yirka, J. (2019). Oracle complexity classes and local measurements on physical Hamiltonians. *arXiv:1909.05981*.

44. Gheorghiu, A., Kapourniotis, T., & Kashefi, E. (2019). Verification of quantum computation: An overview of existing approaches. Theory of Computing Systems, 63(4), 715-808.

45. Girish, U., Raz, R., & Tal, A. (2019). Quantum versus Randomized Communication Complexity, with Efficient Players. *arXiv:1911.02218*.

46. Gilyén, A., Su, Y., Low, G. H., & Wiebe, N. (2019, June). Quantum singular value transformation and beyond: exponential improvements for quantum matrix arithmetics. In *Proceedings of the 51st Annual ACM SIGACT Symposium on Theory of Computing* (pp. 193-204).

47. Gross, D., Liu, Y. K., Flammia, S. T., Becker, S., & Eisert, J. (2010). Quantum state tomography via compressed sensing. *Physical Review Letters*, 105(15), 150401.

48. Gu, Y. (2013). *Moments of random matrices and Weingarten functions* (Doctoral dissertation).

49. Gutoski, G., & Watrous, J. (2007, June). Toward a general theory of quantum games. In *Proceedings of the Thirty-ninth annual ACM symposium on Theory of Computing* (pp. 565-574).

50. Haah, J., Harrow, A. W., Ji, Z., Wu, X., & Yu, N. (2017). Sample-optimal tomography of quantum states. *IEEE Transactions on Information Theory*, 63(9), 5628-5641.

51. Haake, F. (2013). *Quantum Signatures of Chaos* (Vol. 54). Springer Science & Business Media.

52. Harrow, A. W., Hassidim, A., Leung, D. W., & Watrous, J. (2010). Adaptive versus nonadaptive strategies for quantum channel discrimination. *Physical Review A*, 81(3), 032339.

53. Harrow, A. W., & Low, R. A. (2009). Random quantum circuits are approximate 2-designs. *Communications in Mathematical Physics*, 291(1), 257-302.

54. Harrow, A.W. & Saeed, M. Approximate unitary t-designs by short random quantum circuits using nearest neighbor and long-range gates. Plenary talk at *Quantum Information Processing* (QIP 2019).

55. Hayden, P., & Preskill, J. (2007). Black holes as mirrors: quantum information in random subsystems. *Journal of High Energy Physics*, 2007(09), 120.

56. Huang, H. Y., Kueng, R., & Preskill, J. (2020). Predicting many properties of a quantum system from very few measurements. *arXiv:2002.08953*.

57. Huang, H. Y., Kueng, R., & Preskill, J. (2021). Information-theoretic bounds on quantum advantage in machine learning. *arXiv:2101.02464*.

58. Huggins, W.J., McArdle, S., O'Brien, T.E., Lee, J., Rubin, N.C., Boixo, S., Whaley, K.B., Babbush, R., & McClean, J.R. Virtual Distillation for Quantum Error Mitigation. *arXiv:2011.07064*.

59. Ji, Z., Liu, Y. K., & Song, F. (2018, August). Pseudorandom quantum states. In *Annual International Cryptology Conference* (pp. 126-152). Springer, Cham.

60. Jordan, S. P., Lee, K. S., & Preskill, J. (2012). Quantum algorithms for quantum field theories. *Science*, 336(6085), 1130-1133.

61. Kessler, E. M., Lovchinsky, I., Sushkov, A. O., & Lukin, M. D. (2014). Quantum error correction for metrology. *Physical Review Letters*, 112(15), 150802.

62. Kitaev, A. Y. (1995). Quantum measurements and the Abelian stabilizer problem. *quant-ph/9511026*.

63. Kitaev, A. Y. (2003). Fault-tolerant quantum computation by anyons. *Annals of Physics*, 303(1), 2-30.

64. Kitaev, A., & Watrous, J. (2000, May). Parallelization, amplification, and exponential time simulation of quantum interactive proof systems. In *Proceedings of the Thirty-second Annual ACM symposium on Theory of Computing* (pp. 608-617).

65. Knill, E., Laflamme, R., & Zurek, W. H. (1998). Resilient quantum computation. *Science*, 279(5349), 342-345.

66. Koczar, B. Exponential Error Suppression for Near-Term Quantum Devices. *arXiv:2011.05942*.

67. Kok, P., Braunstein, S. L., & Dowling, J. P. (2004). Quantum lithography, entanglement and Heisenberg-limited parameter estimation. *Journal of Optics B: Quantum and Semiclassical Optics*, 6(8), S811.

68. Kueng, R., Rauhut, H., & Terstiege, U. (2017). Low rank matrix recovery from rank one measurements. *Applied and Computational Harmonic Analysis*, 42(1), 88-116.

69. Lashkari, N., Stanford, D., Hastings, M., Osborne, T., & Hayden, P. (2013). Towards the fast scrambling conjecture. *Journal of High Energy Physics*, 2013(4), 22.

70. Ledoux, M. (2001). *The concentration of measure phenomenon* (No. 89). American Mathematical Society.

71. Li, Z., Zou, L., & Hsieh, T. H. (2020). Hamiltonian Tomography via Quantum Quench. *Physical Review Letters*, 124(16), 160502.

72. Low, R. A. (2009). Large deviation bounds for *k*-designs. *Proceedings of the Royal Society A: Mathematical, Physical and Engineering Sciences*, 465(2111), 3289-3308.

73. Matsumoto, S. (2013). Weingarten calculus for matrix ensembles associated with compact symmetric spaces. *arXiv:1301.5401*.

74. Millikan, R. A. (1910). XXII. A new modification of the cloud method of determining the elementary electrical charge and the most probable value of that charge. *The London, Edinburgh, and Dublin Philosophical Magazine and Journal of Science*, 19(110), 209-228.

75. Miyazaki, J., Soeda, A., & Murao, M. (2019). Complex conjugation supermap of unitary quantum maps and its universal implementation protocol. *Physical Review Research*, 1(1), 013007.

76. Nielsen, M. A., & Chuang, I. (2002). *Quantum Computation and Quantum Information*. Cambridge University Press.

77. O'Donnell, R., & Wright, J. (2016, June). Efficient quantum tomography. In *Proceedings of the forty-eighth annual ACM symposium on Theory of Computing* (pp. 899-912).

78. Ozeri, R. (2013). Heisenberg limited metrology using quantum error-correction codes. *arXiv:1310.3432*.

79. Papadimitriou, C. (1994). *Computatational Complexity*. Addison-Wesley.

80. Pollock, F. A., Rodríguez-Rosario, C., Frauenheim, T., Paternostro, M., & Modi, K. (2018). Non-Markovian quantum processes: Complete framework and efficient characterization. *Physical Review A*, 97(1), 012127.

81. Preskill, J. (2018). Quantum Computing in the NISQ era and beyond. *Quantum*, 2, 79.

82. Quintino, M. T., Dong, Q., Shimbo, A., Soeda, A., & Murao, M. (2019). Probabilistic exact universal quantum circuits for transforming unitary operations. *Physical Review A*, 100(6), 062339.

83. Quintino, M. T., Dong, Q., Shimbo, A., Soeda, A., & Murao, M. (2019). Reversing unknown quantum transformations: Universal quantum circuit for inverting general unitary operations. *Physical Review Letters*, 123(21), 210502.

84. Radhakrishnan, J., Rötteler, M., & Sen, P. (2009). Random measurement bases, quantum state distinction and applications to the hidden subgroup problem. *Algorithmica*, 55(3), 490-516.

85. Raz, R. (1999, May). Exponential separation of quantum and classical communication complexity. In *Proceedings of the Thirty-First Annual ACM symposium on Theory of Computing* (pp. 358-367).

86. Regev, O., & Klartag, B. A. (2011, June). Quantum one-way communication can be exponentially stronger than classical communication. In *Proceedings of the Forty-third annual ACM Symposium on Theory of Computing* (pp. 31-40).

87. Reichardt, B. W., Unger, F., & Vazirani, U. (2013). Classical command of quantum systems. *Nature*, 496(7446), 456-460.

88. Riofrío, C. A., Gross, D., Flammia, S. T., Monz, T., Nigg, D., Blatt, R., & Eisert, J. (2017). Experimental quantum compressed sensing for a seven-qubit system. *Nature Communications*, 8(1), 1-8.

89. Schmitt, S., Gefen, T., Stürner, F. M., Unden, T., Wolff, G., Müller, C., ... & Meijer, J. (2017). Submillihertz magnetic spectroscopy performed with a nanoscale quantum sensor. *Science*, 356(6340), 832-837.

90. Sheridan, L., Maslov, D., & Mosca, M. (2009). Approximating fractional time quantum evolution. *Journal of Physics A: Mathematical and Theoretical*, 42(18), 185302.

91. Simon, D. R. (1997). On the power of quantum computation. *SIAM Journal on Computing*, 26(5), 1474-1483.

92. Thompson, J., Gu, M., Modi, K., & Vedral, V. (2013). Quantum computing with black-box subroutines. *arXiv:1310.2927*.

93. de Wolf, R. (2002). Quantum communication and complexity. *Theoretical Computer Science*, 287(1), 337-353.

94. Yao, A. C. C. (1993, November). Quantum circuit complexity. In *Proceedings of 1993 IEEE 34th Annual Foundations of Computer Science* (pp. 352-361). IEEE.

95. Yu, N. (2020). Sample efficient tomography via Pauli Measurements. *arXiv:2009.04610*.

96. Zhou, S., Zhang, M., Preskill, J., & Jiang, L. (2018). Achieving the Heisenberg limit in quantum metrology using quantum error correction. *Nature Communications*, 9(1), 1-11.
